# Supplementary figures and images for: Patient-specific midbrain organoids with CRISPR correction recapitulate neuronopathic Gaucher disease phenotypes and enable evaluation of novel therapies
Source: eLife. 2026 Jun 23;15:RP109518. doi: 10.7554/eLife.109518 (PMC13290227; doi:10.7554/eLife.109518)

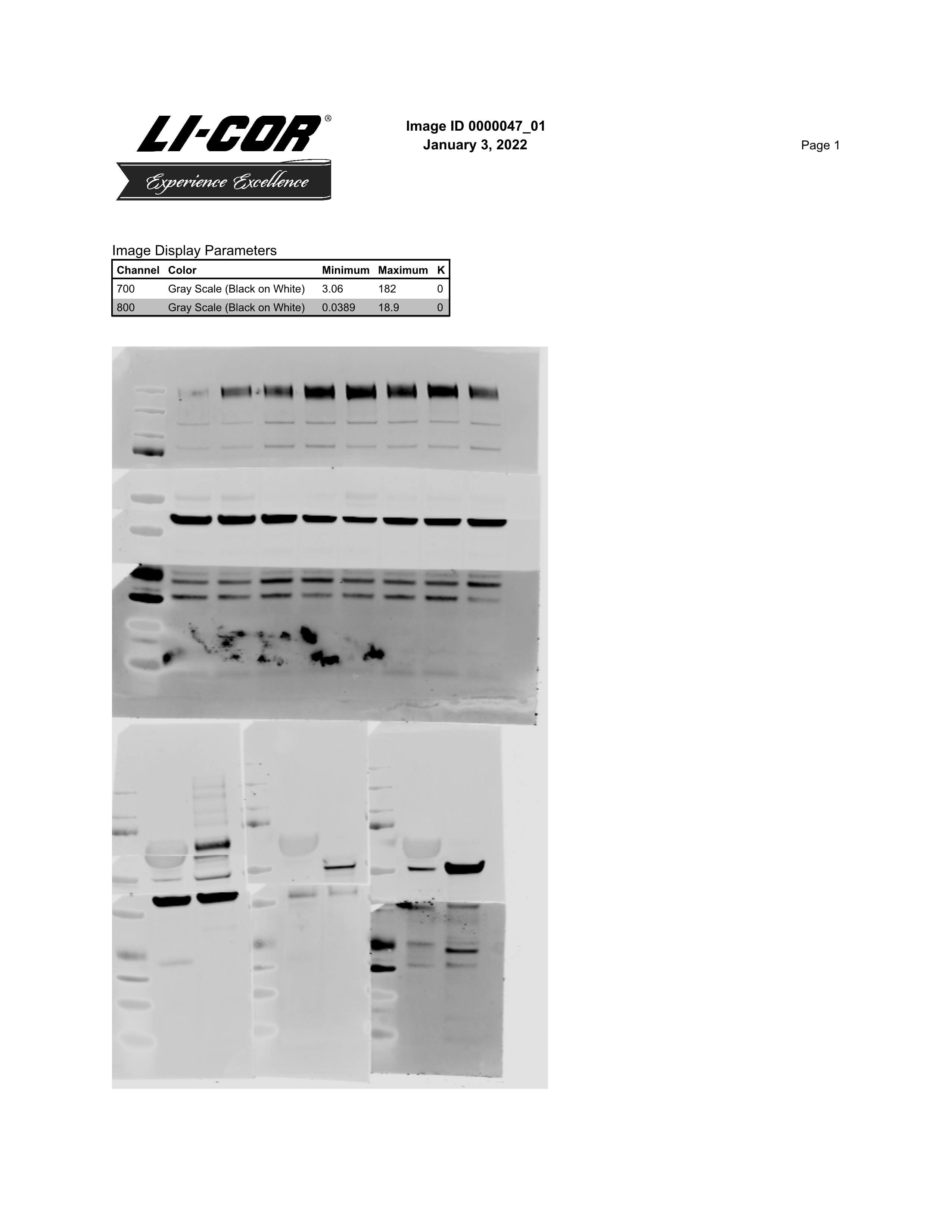

Supplement: Figure 1—source data 1. [file elife-109518-fig1-data1.zip › Figure 1-source data 1/Gel 1 and P1-3 4_1_Figure 1E.tif]

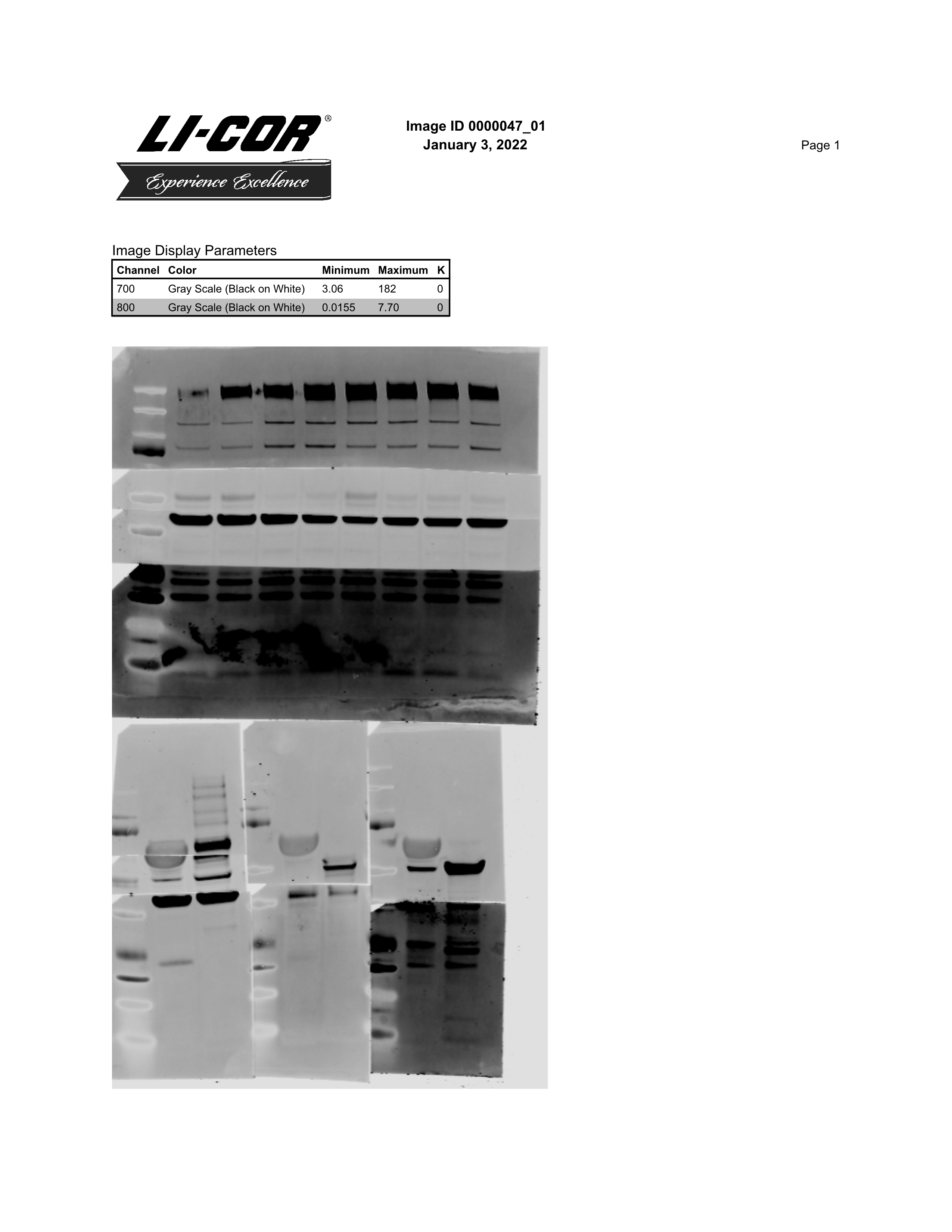

Supplement: Figure 1—source data 1. [file elife-109518-fig1-data1.zip › Figure 1-source data 1/Gel 1 and P1-3 5_1_Figure 1E.tif]

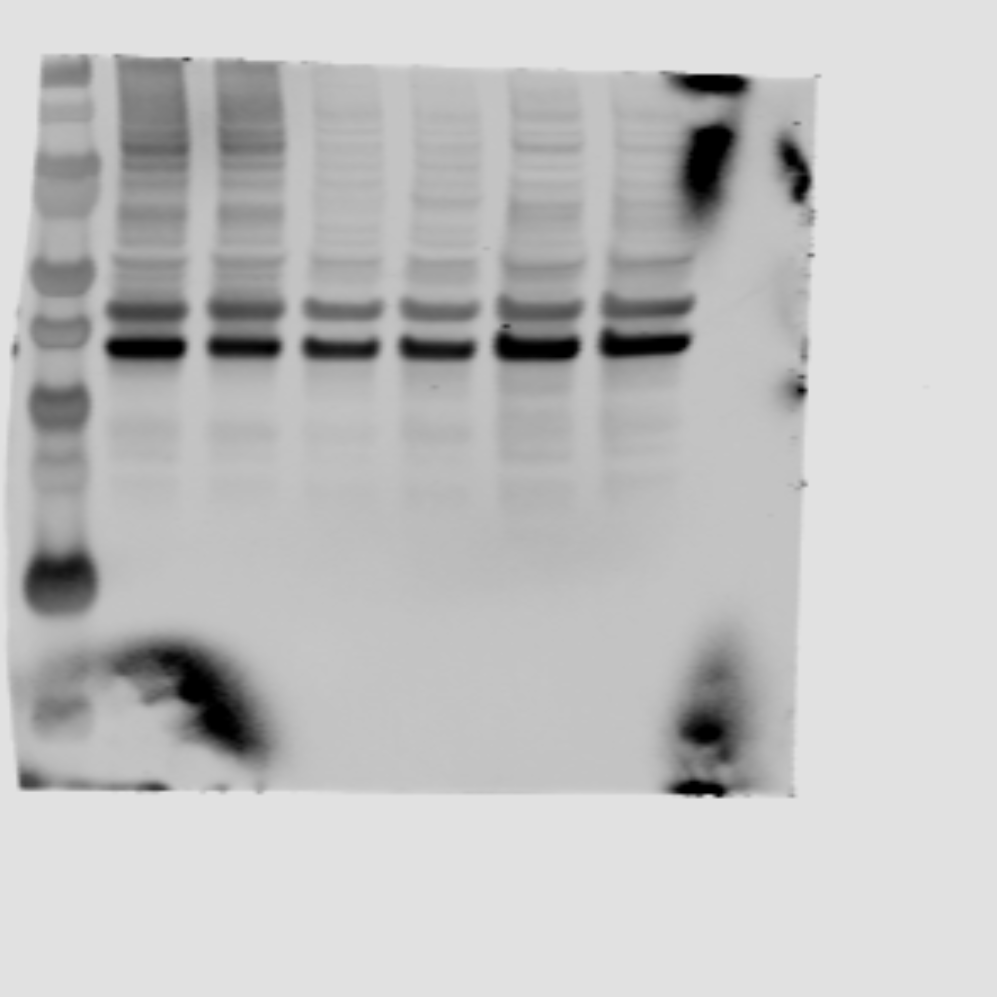

Supplement: Figure 2—source data 1. [file elife-109518-fig2-data1.zip › Figure 2-source data 1/GAPDH MLO.tif]

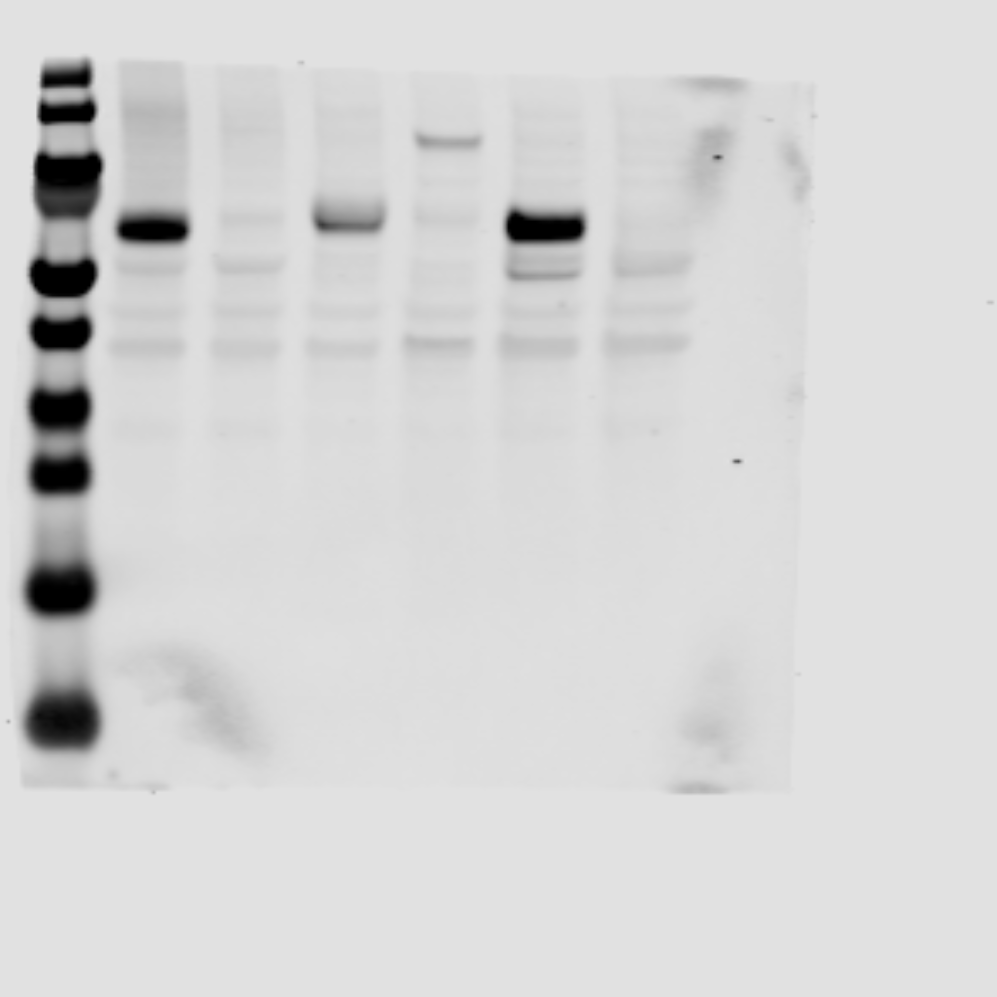

Supplement: Figure 2—source data 1. [file elife-109518-fig2-data1.zip › Figure 2-source data 1/hGCase MLO d.tif]

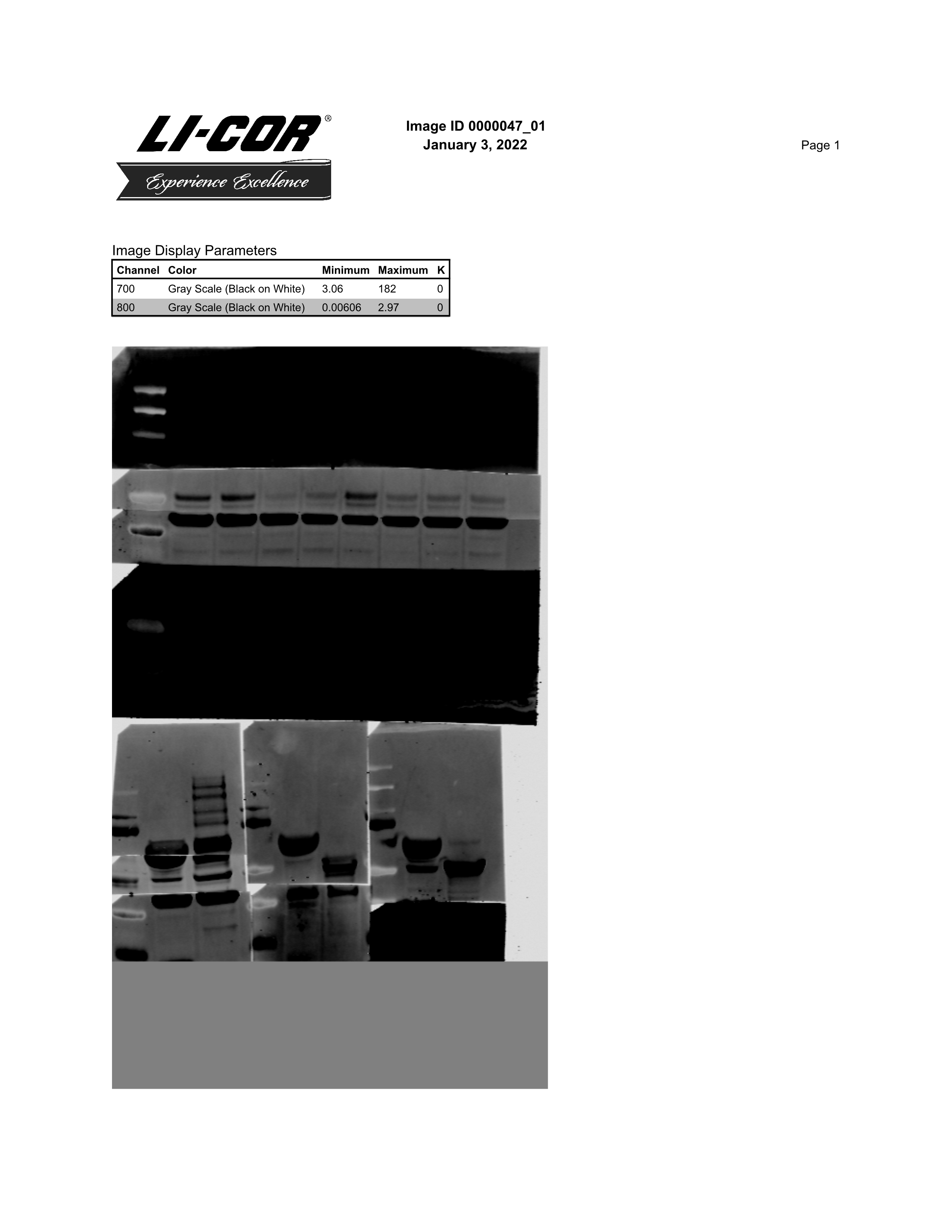

Supplement: Figure 3—source data 1. [file elife-109518-fig3-data1.zip › Figure 3-source data 1/Gel 1 and P1-3 3_Fig3G_FOXA2_1.tif]

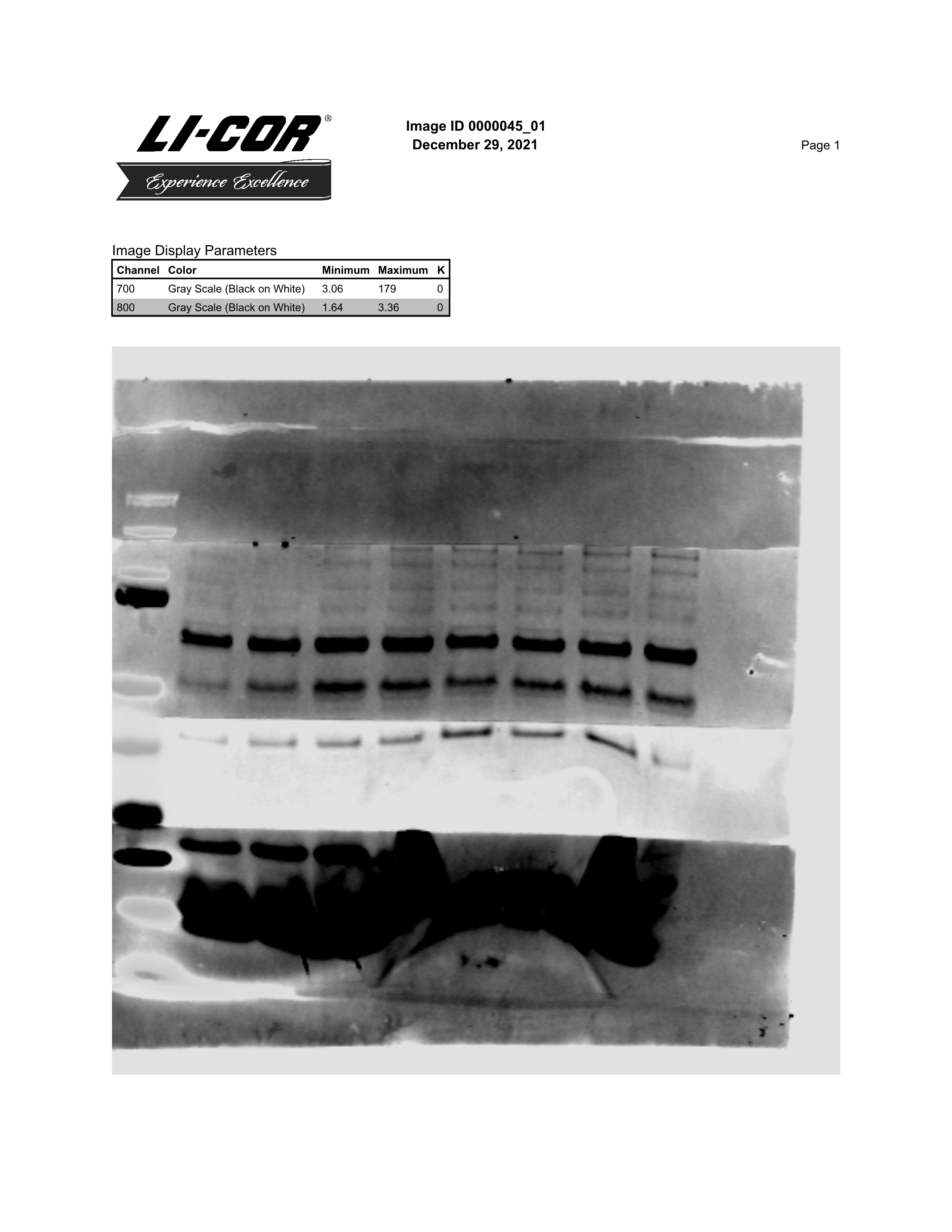

Supplement: Figure 3—source data 1. [file elife-109518-fig3-data1.zip › Figure 3-source data 1/Gel 1 M1-4 2_Fig3G_MAP2_1.tif]

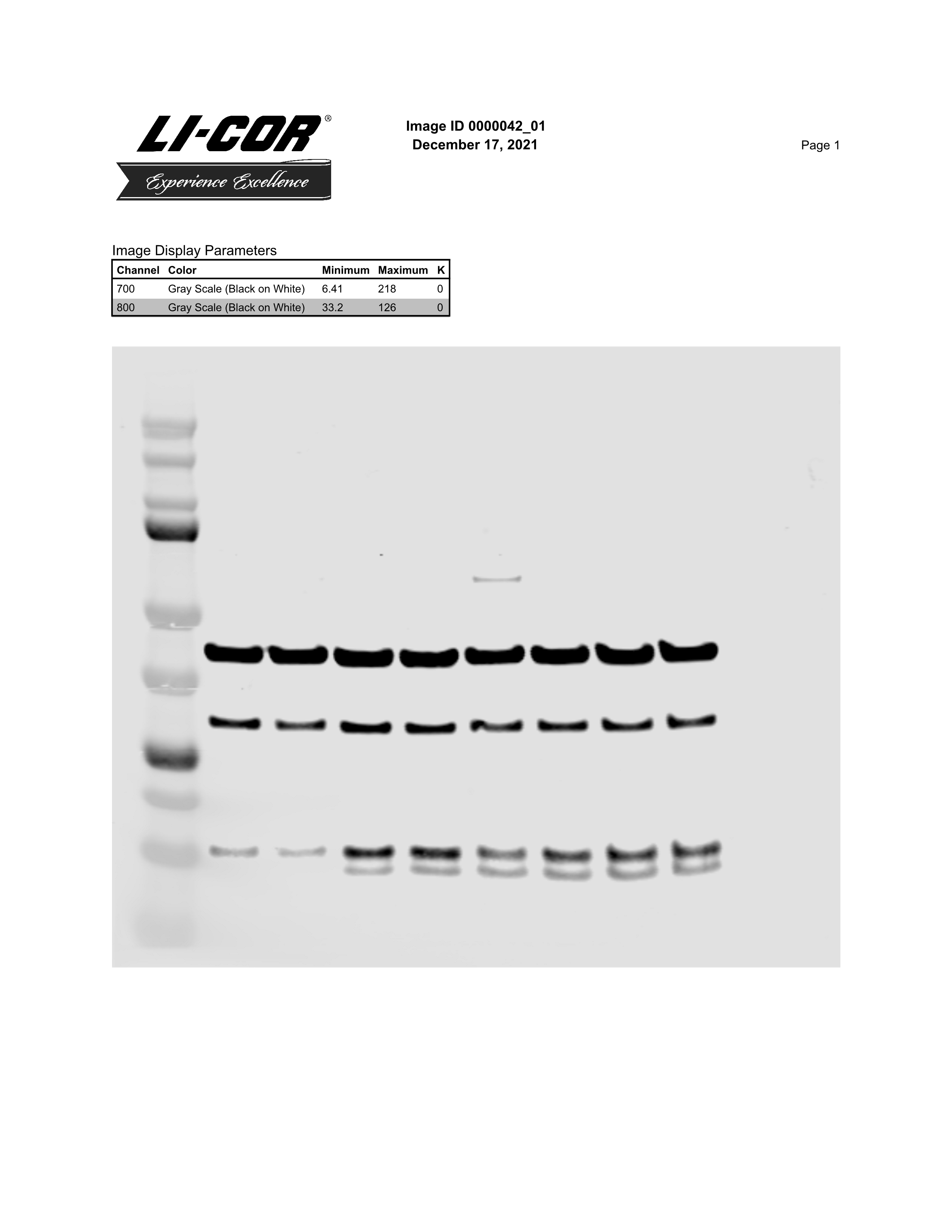

Supplement: Figure 3—source data 1. [file elife-109518-fig3-data1.zip › Figure 3-source data 1/Gel 1 M1-5 3_Fig3G_actin_1.tif]

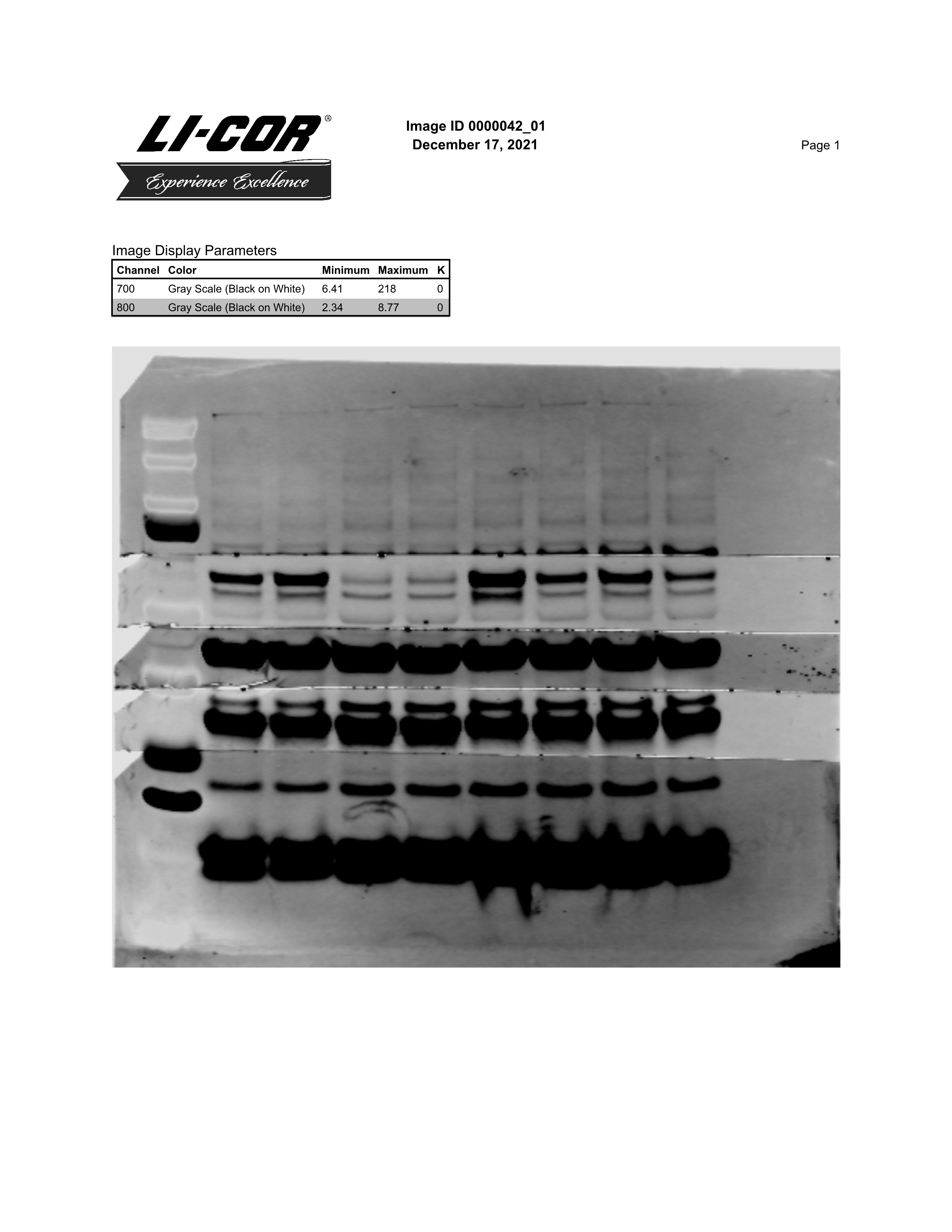

Supplement: Figure 3—source data 1. [file elife-109518-fig3-data1.zip › Figure 3-source data 1/Gel 1 M1-5 4_Fig3G_TH_1.tif]

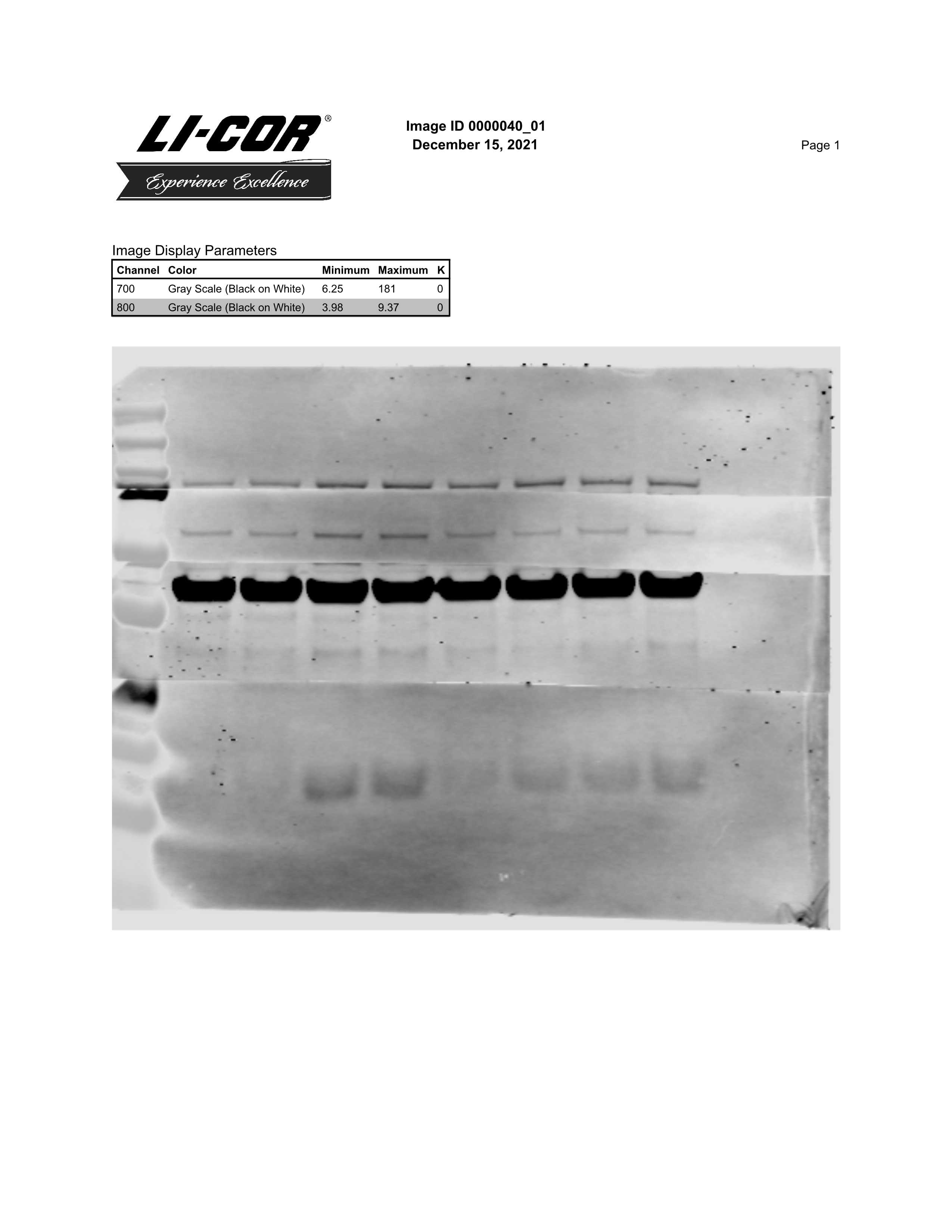

Supplement: Figure 4—source data 1. [file elife-109518-fig4-data1.zip › Figure 4-source data 1/Gel 1 M1-4 1_Figure 4O_1.tif]

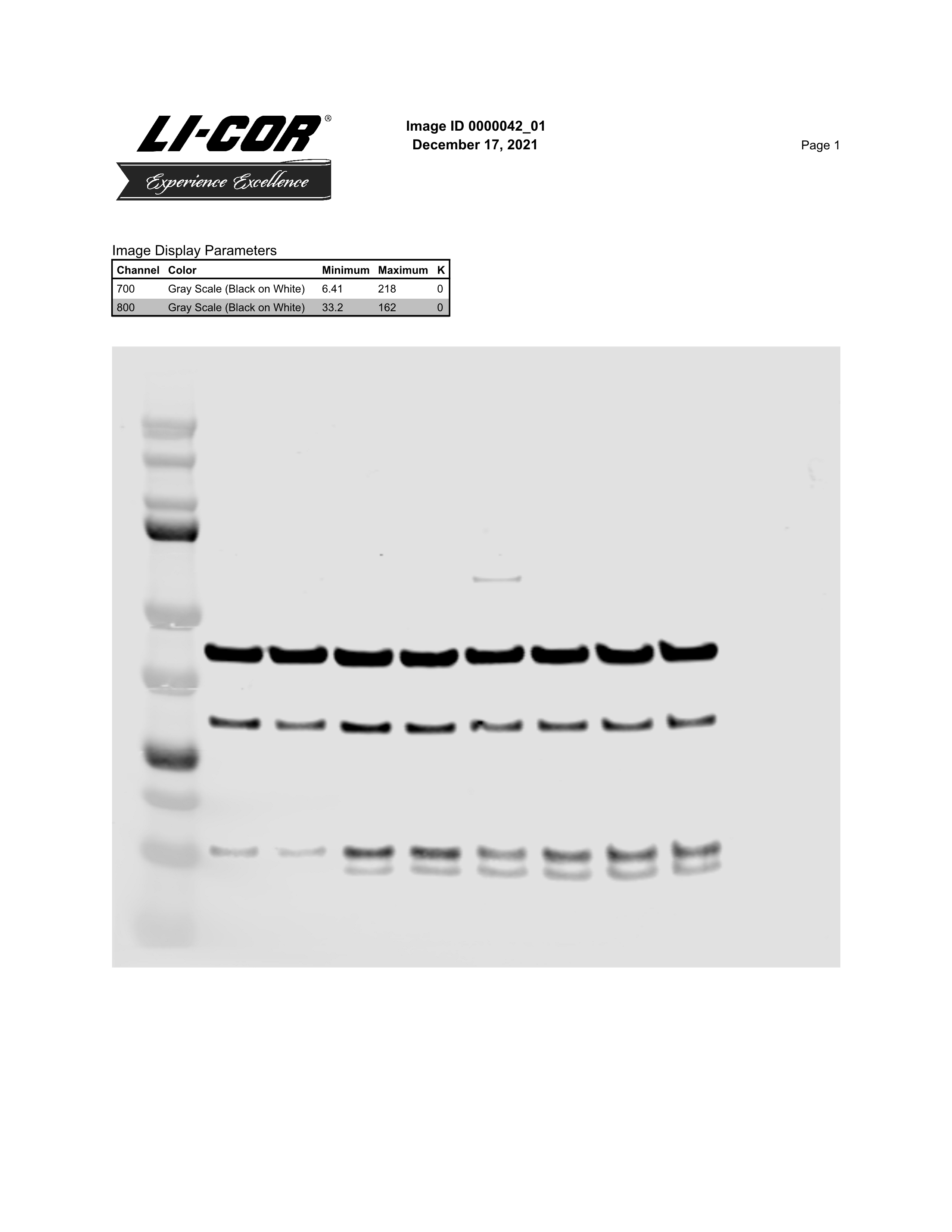

Supplement: Figure 4—source data 1. [file elife-109518-fig4-data1.zip › Figure 4-source data 1/Gel 1 M1-5 1_Figure 4B_1.tif]

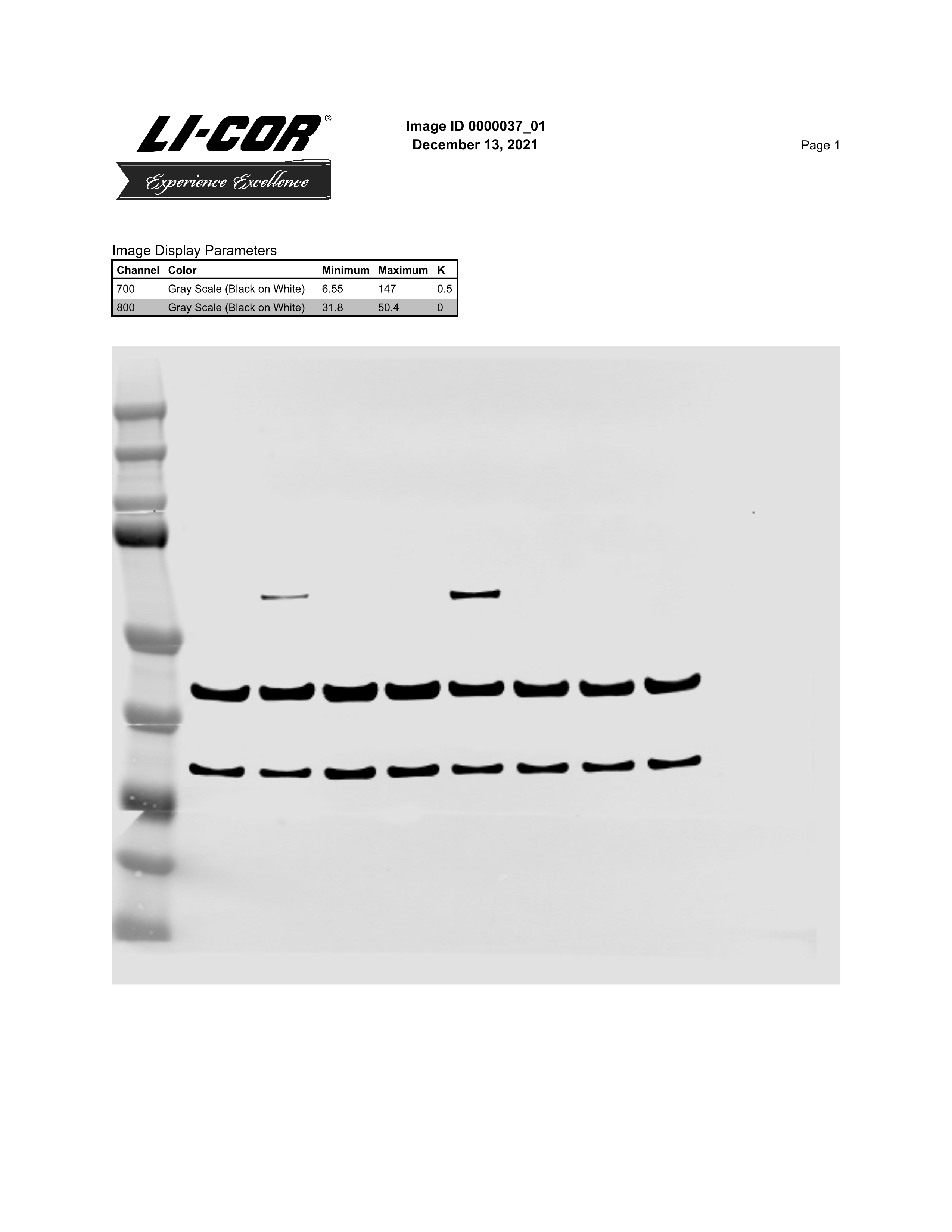

Supplement: Figure 4—source data 1. [file elife-109518-fig4-data1.zip › Figure 4-source data 1/Gel 1 M1-5 2_Figure 4O_1.tif]

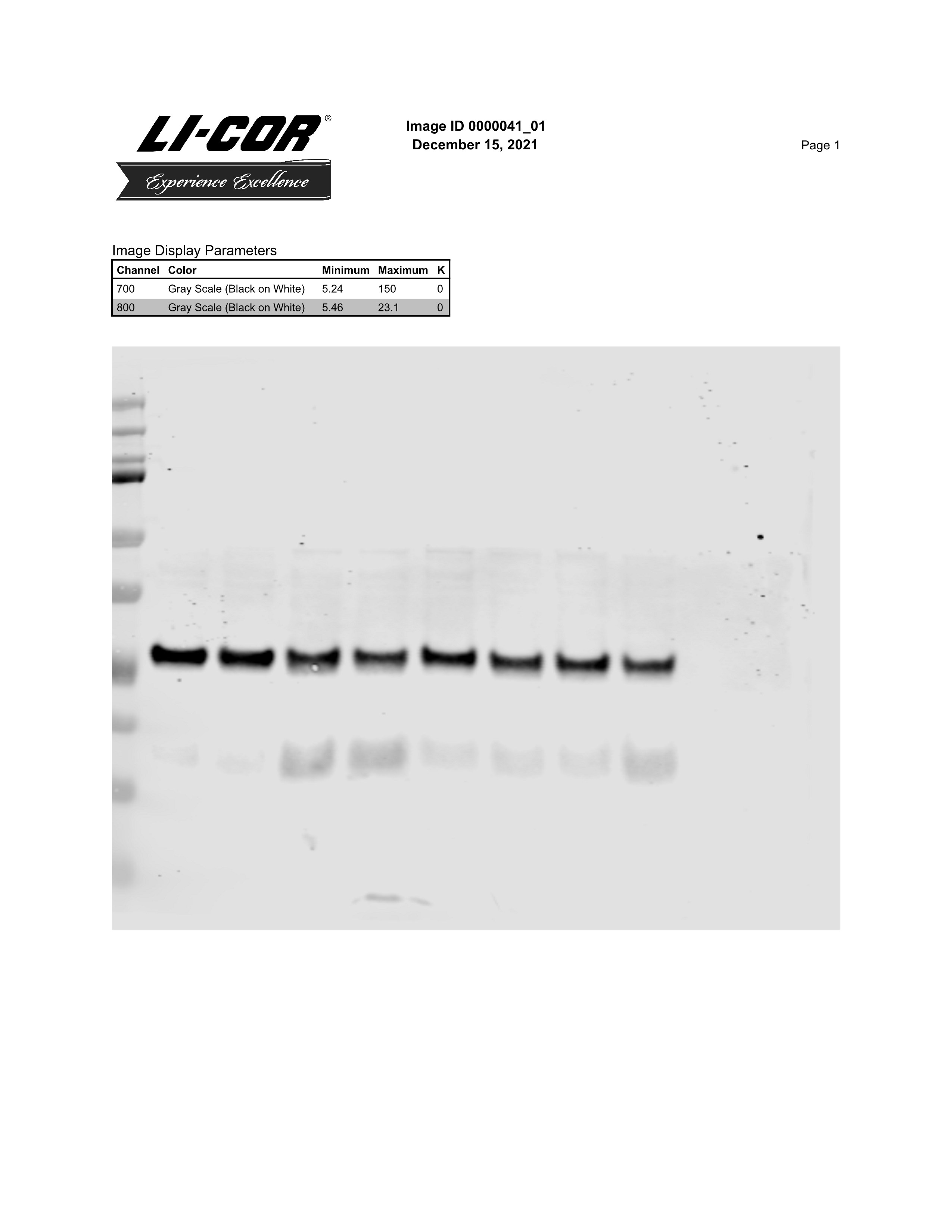

Supplement: Figure 4—source data 1. [file elife-109518-fig4-data1.zip › Figure 4-source data 1/Gel 2 M5-8 1_Figure 4K_1.tif]

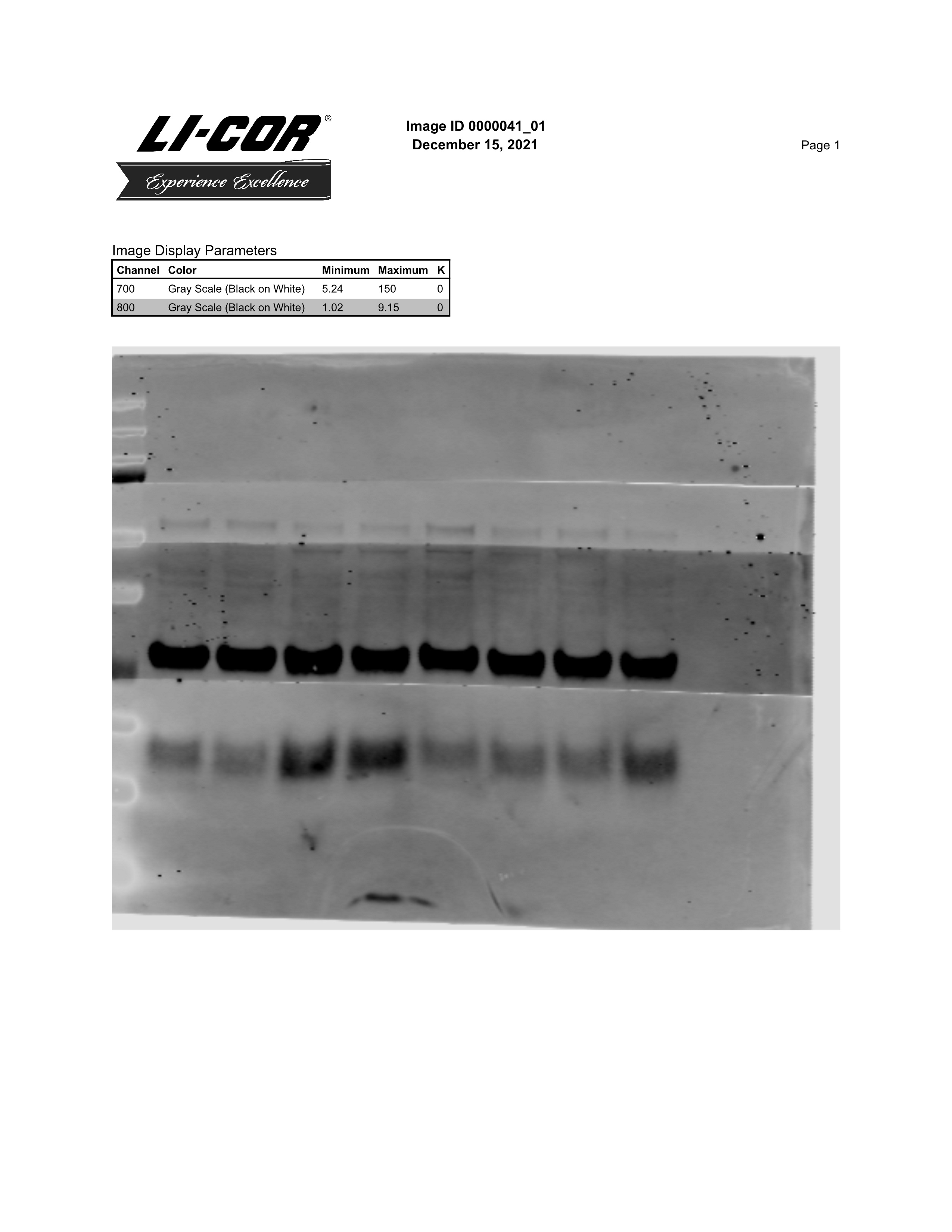

Supplement: Figure 4—source data 1. [file elife-109518-fig4-data1.zip › Figure 4-source data 1/Gel 2 M5-8 3_Figure 4O_1.tif]

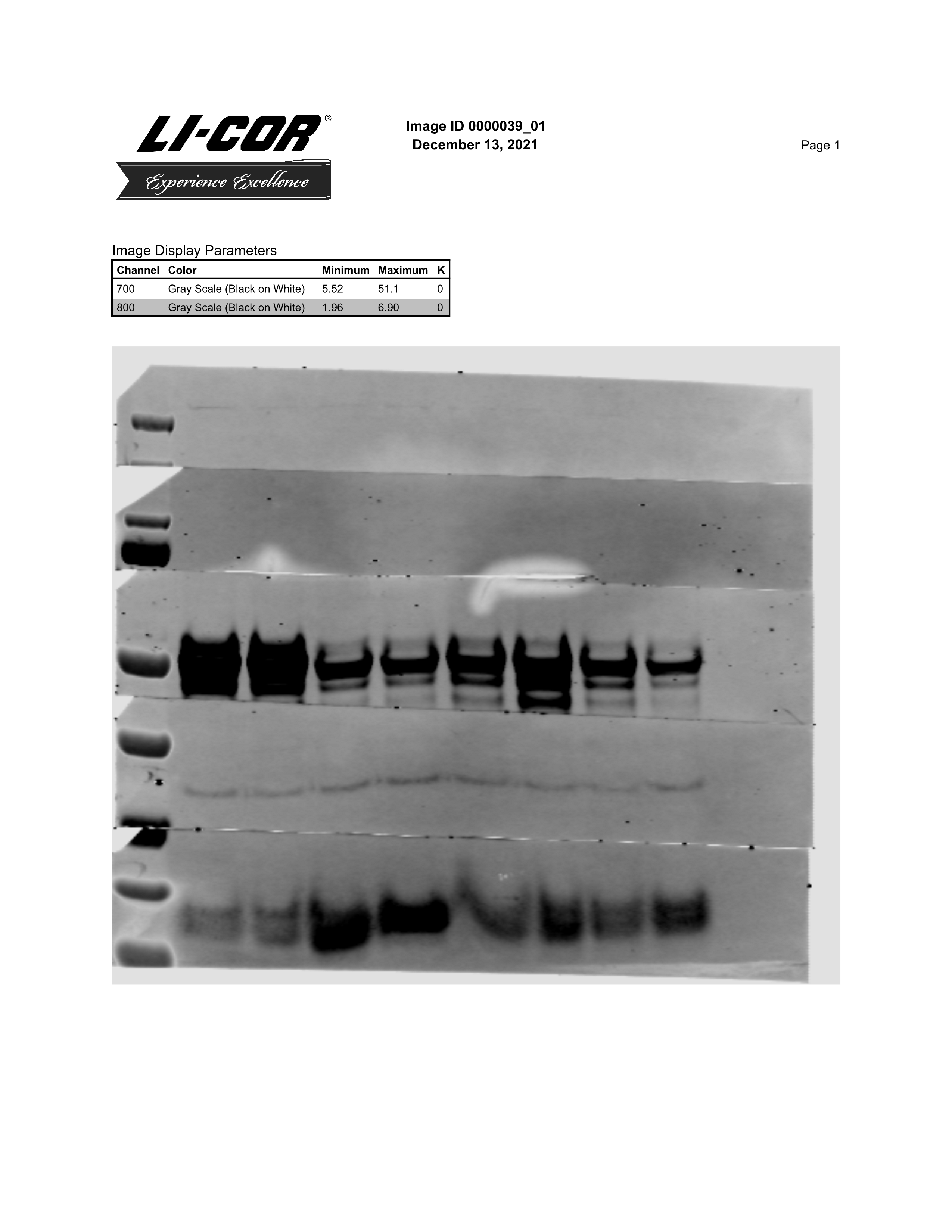

Supplement: Figure 4—source data 1. [file elife-109518-fig4-data1.zip › Figure 4-source data 1/Gel 2 M6-10 1_Figure 4O_1.tif]

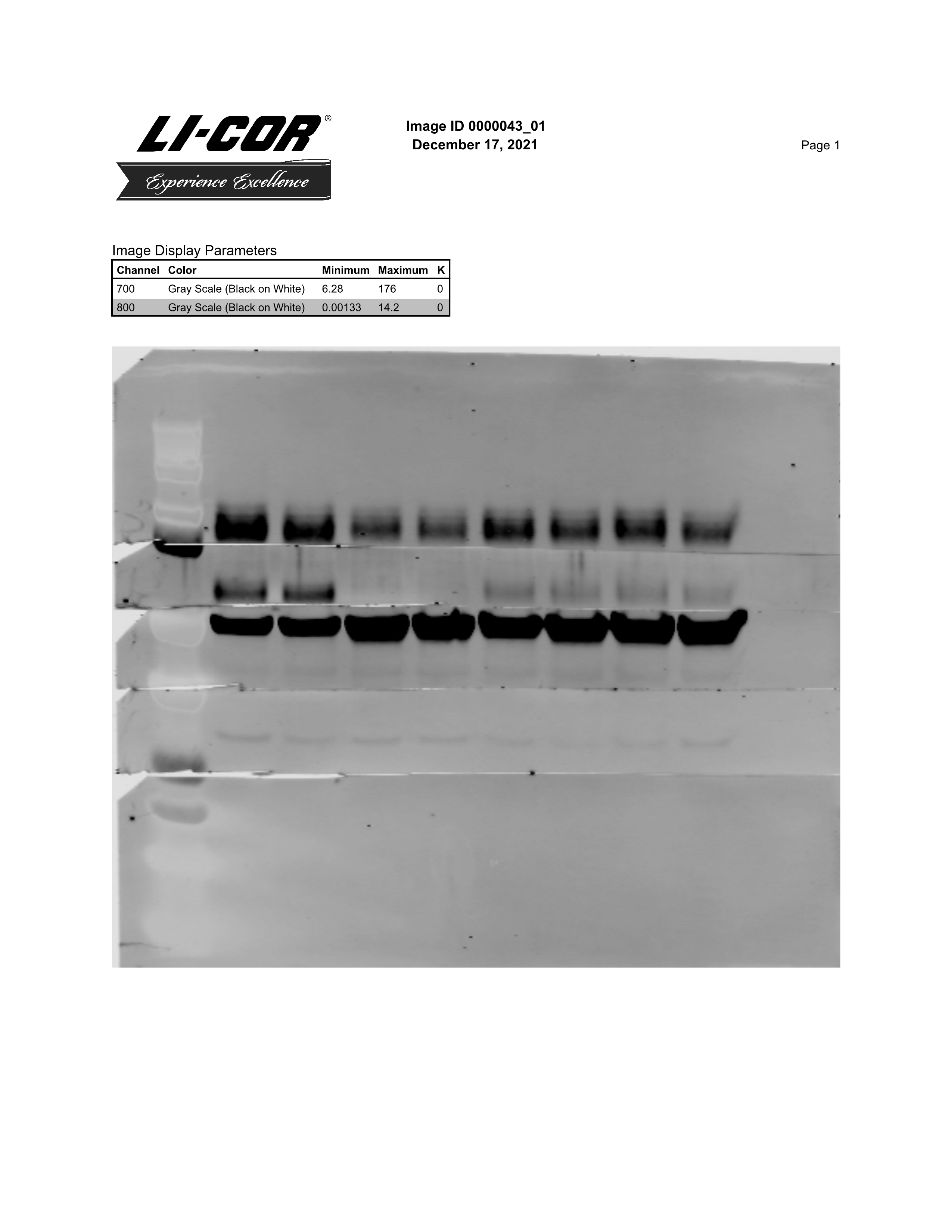

Supplement: Figure 4—source data 1. [file elife-109518-fig4-data1.zip › Figure 4-source data 1/Gel 2 M6-10 5_Figure 4B 4K_1.tif]

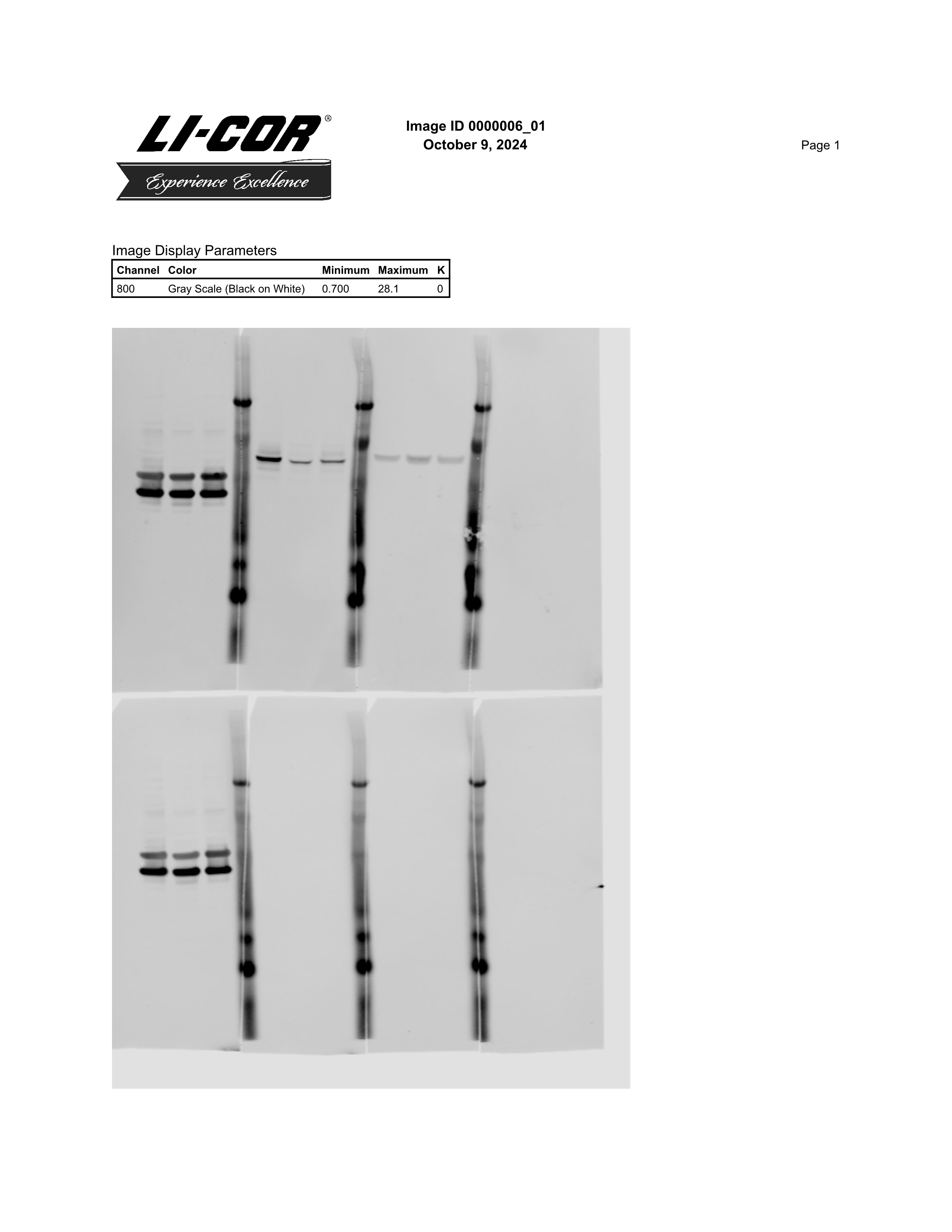

Supplement: Figure 4—source data 1. [file elife-109518-fig4-data1.zip › Figure 4-source data 1/Gel1&2 green1_Figure 4H 4K_1.tif]

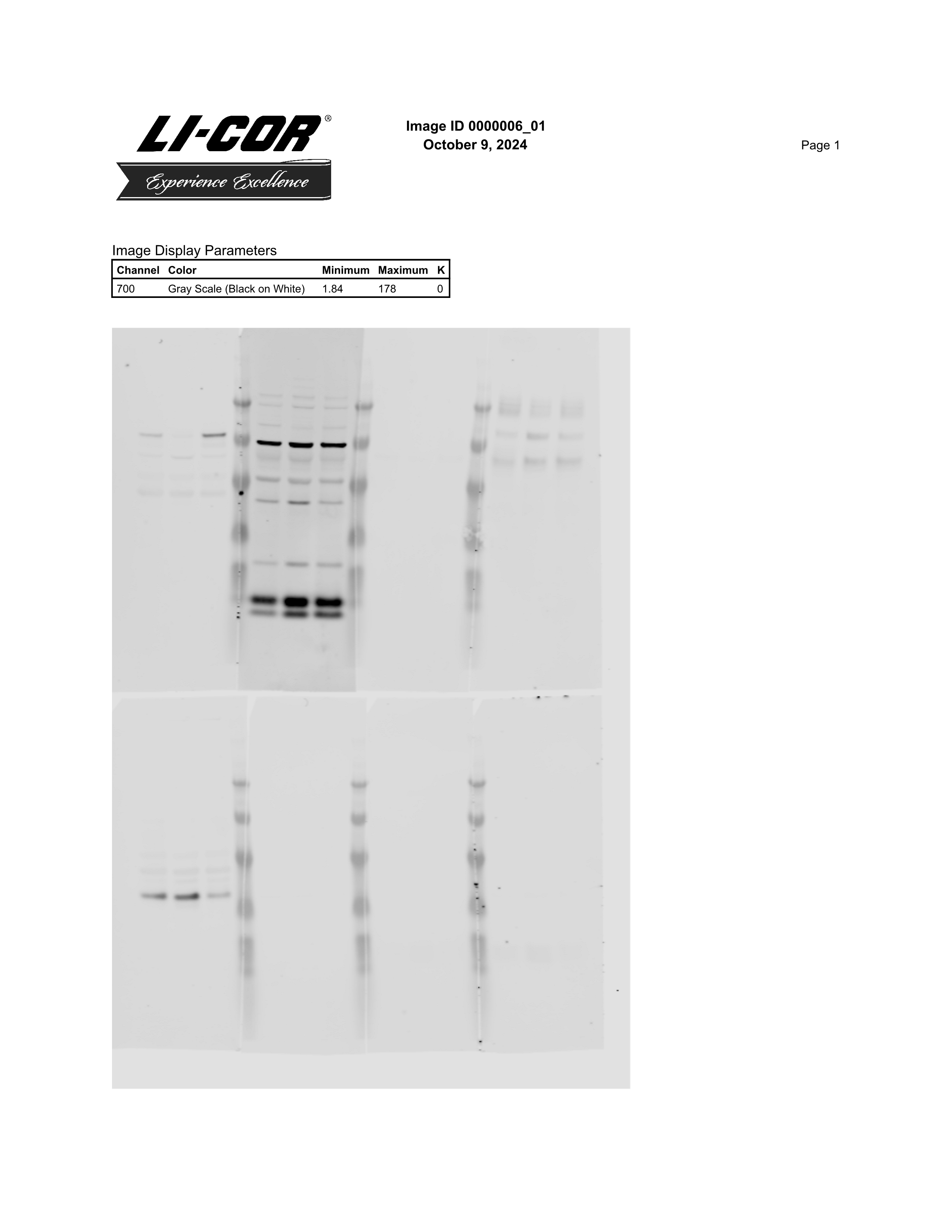

Supplement: Figure 4—source data 1. [file elife-109518-fig4-data1.zip › Figure 4-source data 1/Gel1&2 red3_Figure 4M_1.tif]

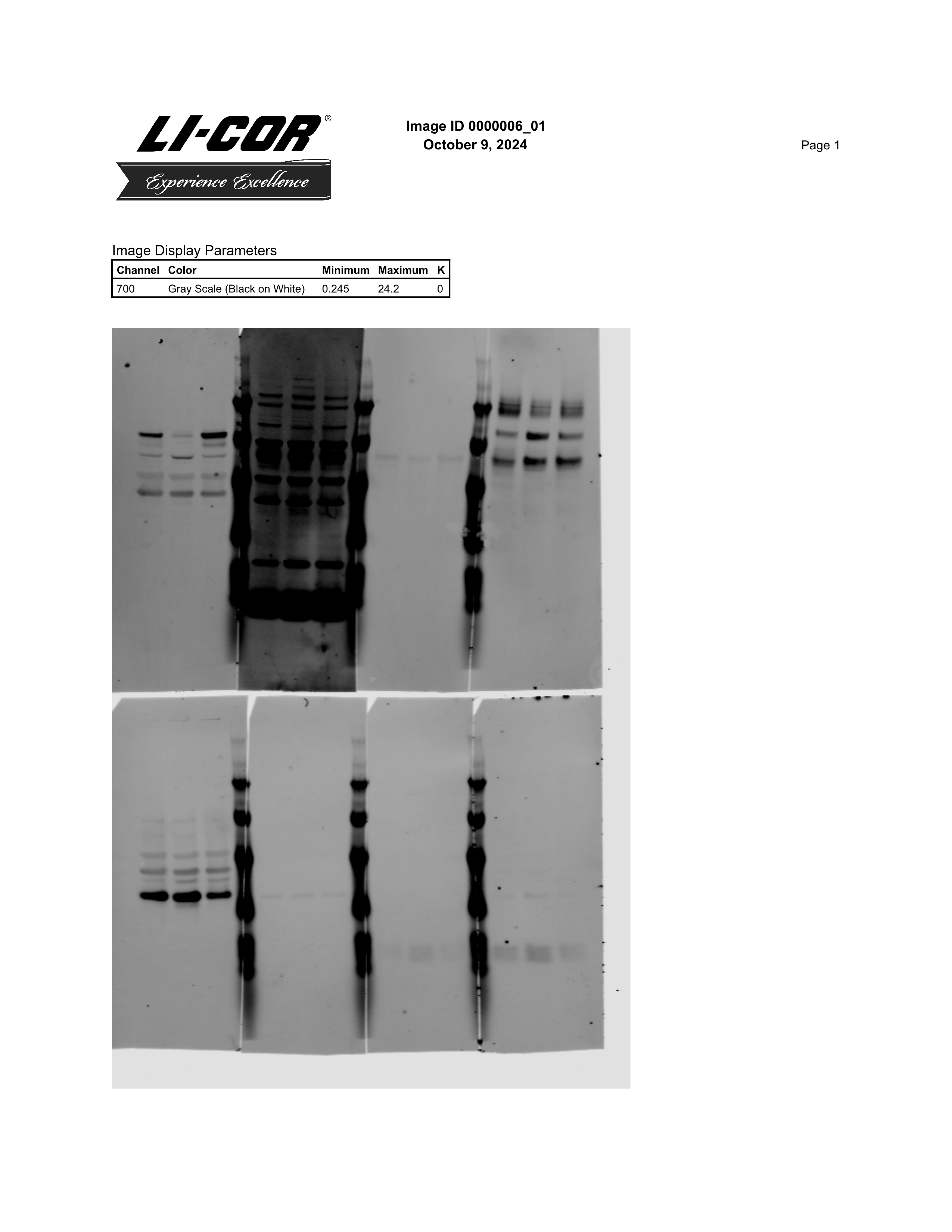

Supplement: Figure 4—source data 1. [file elife-109518-fig4-data1.zip › Figure 4-source data 1/Gel1&2 red6_Figure 4H_1.tif]

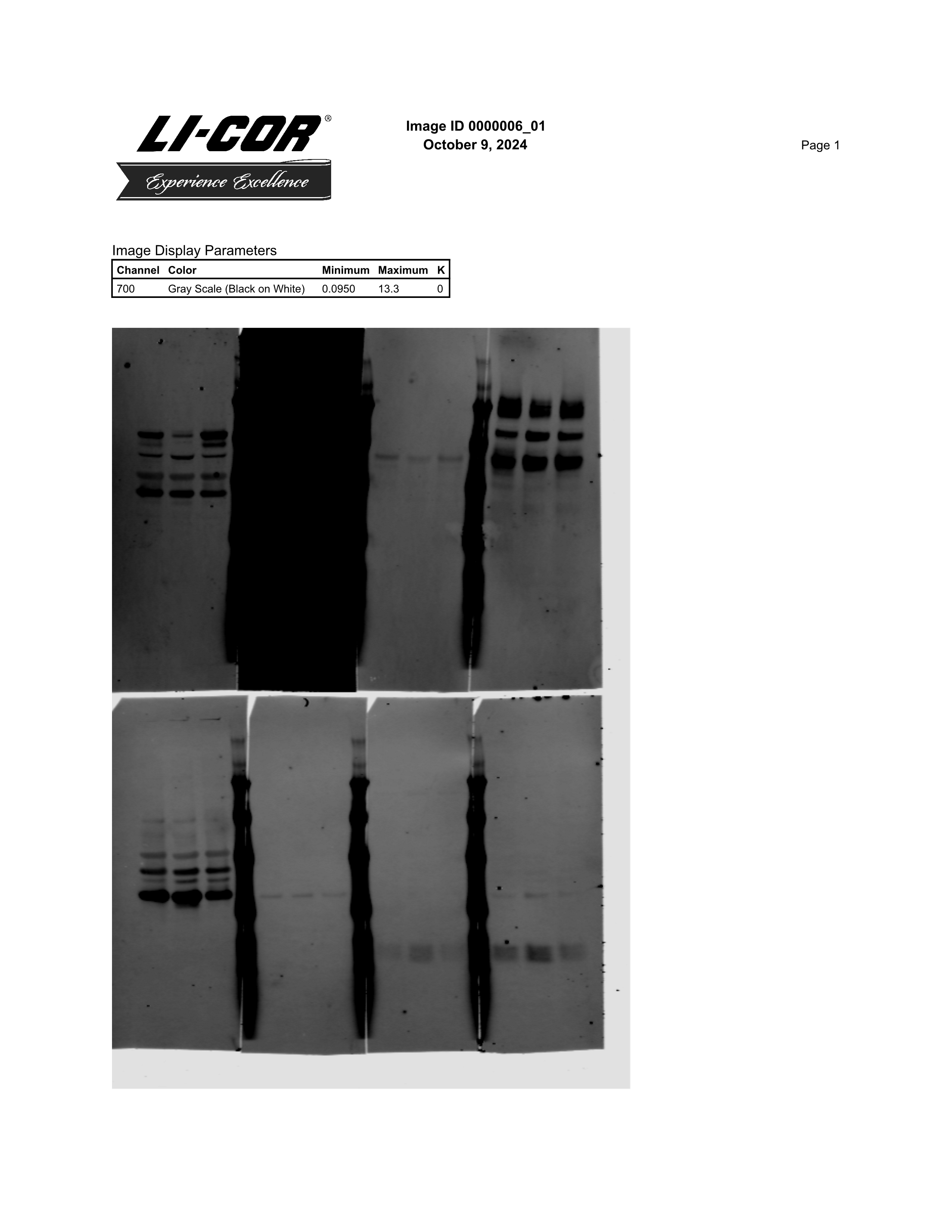

Supplement: Figure 4—source data 1. [file elife-109518-fig4-data1.zip › Figure 4-source data 1/Gel1&2 red8_Figure 4H_1.tif]

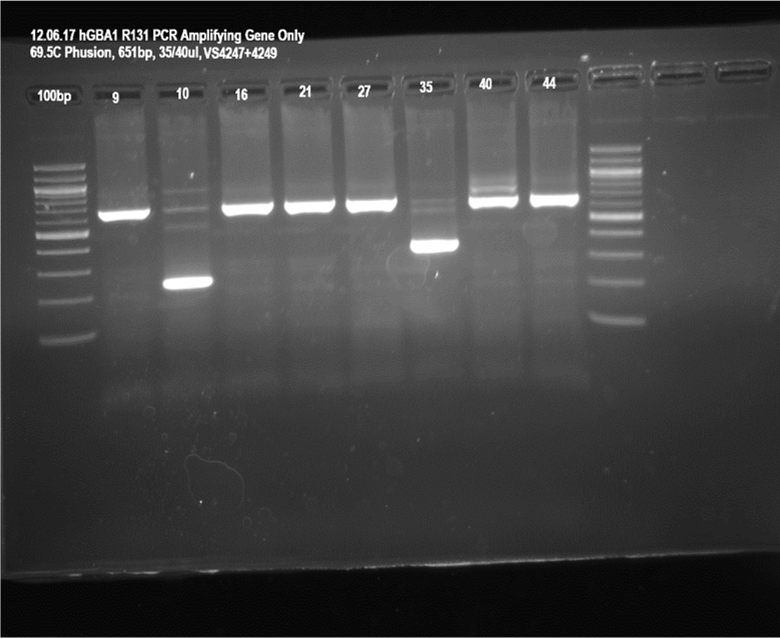

Supplement: Figure 4—figure supplement 1—source data 1. [file elife-109518-fig4-figsupp1-data1.zip › Figure 4-figure supplement 1 - source data 1/12.06.17 hGBA1 R131 PCR Amplifying Gene only (top panel).tif]

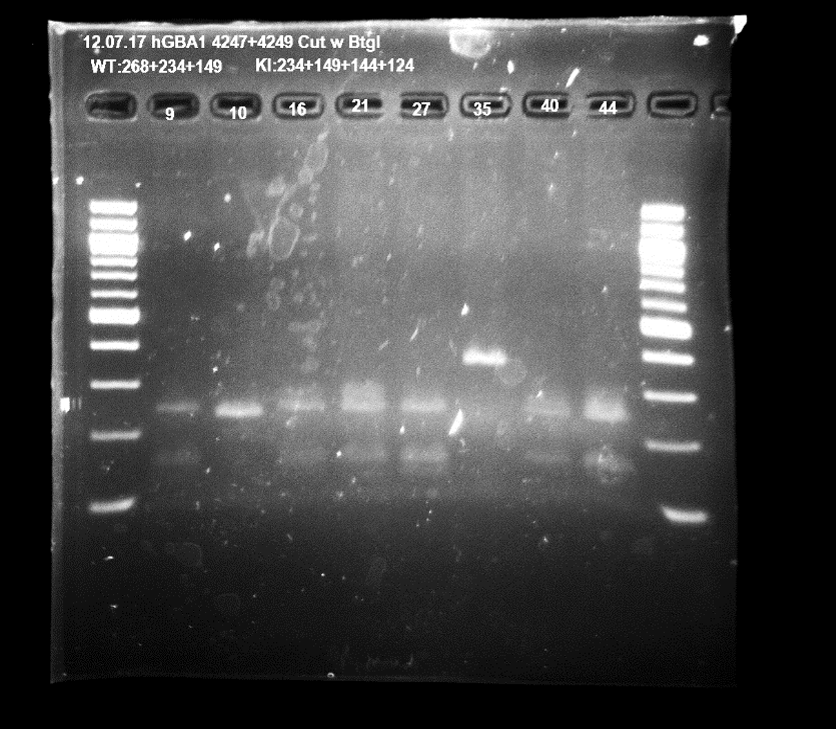

Supplement: Figure 4—figure supplement 1—source data 1. [file elife-109518-fig4-figsupp1-data1.zip › Figure 4-figure supplement 1 - source data 1/12.07.17 hGBA1 4247+4249 Cut w Btgl (bottom panel).tif]

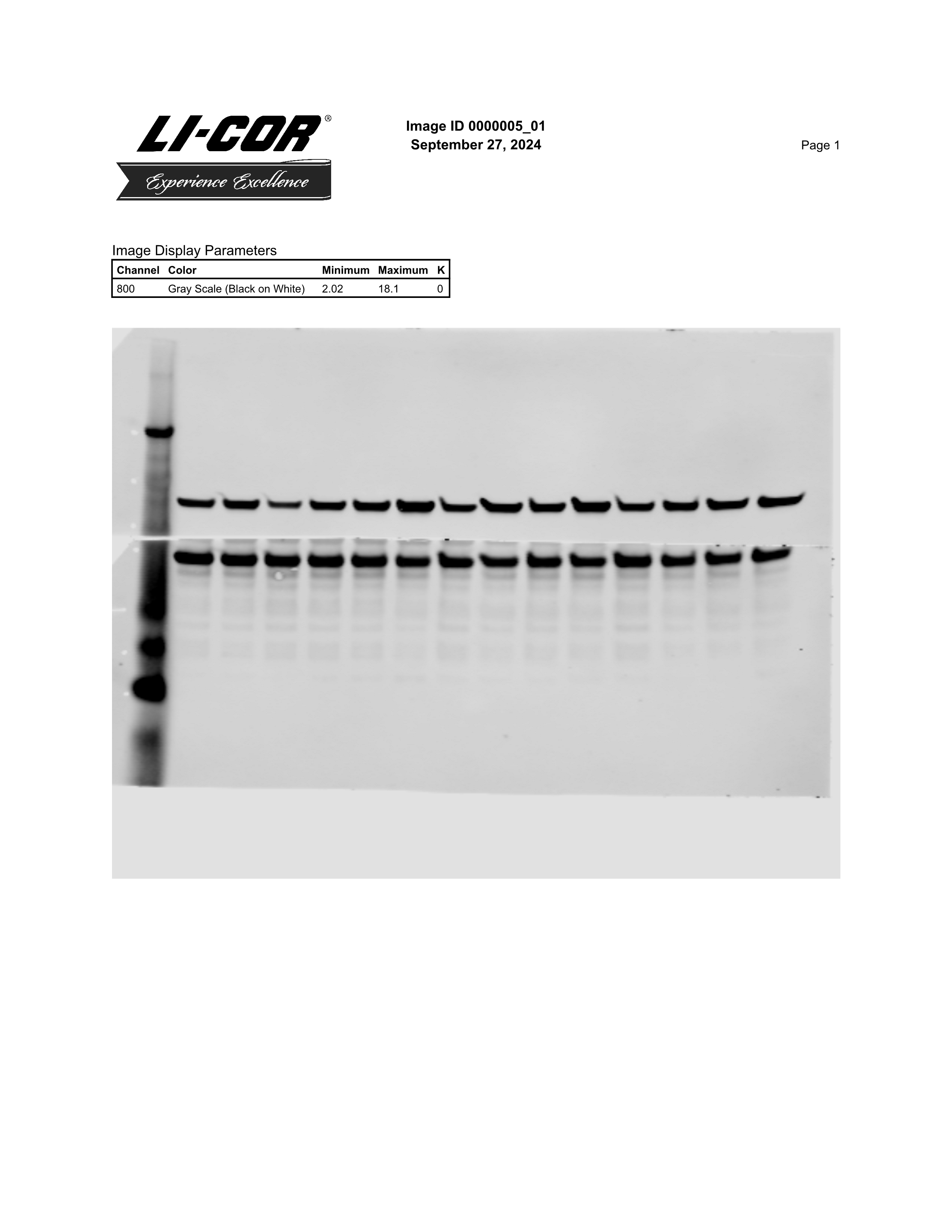

Supplement: Figure 5—source data 1. [file elife-109518-fig5-data1.zip › Figure 5-source data 1/Gel2 TH_ GAPDH 1_Figure 5J_1.tif]

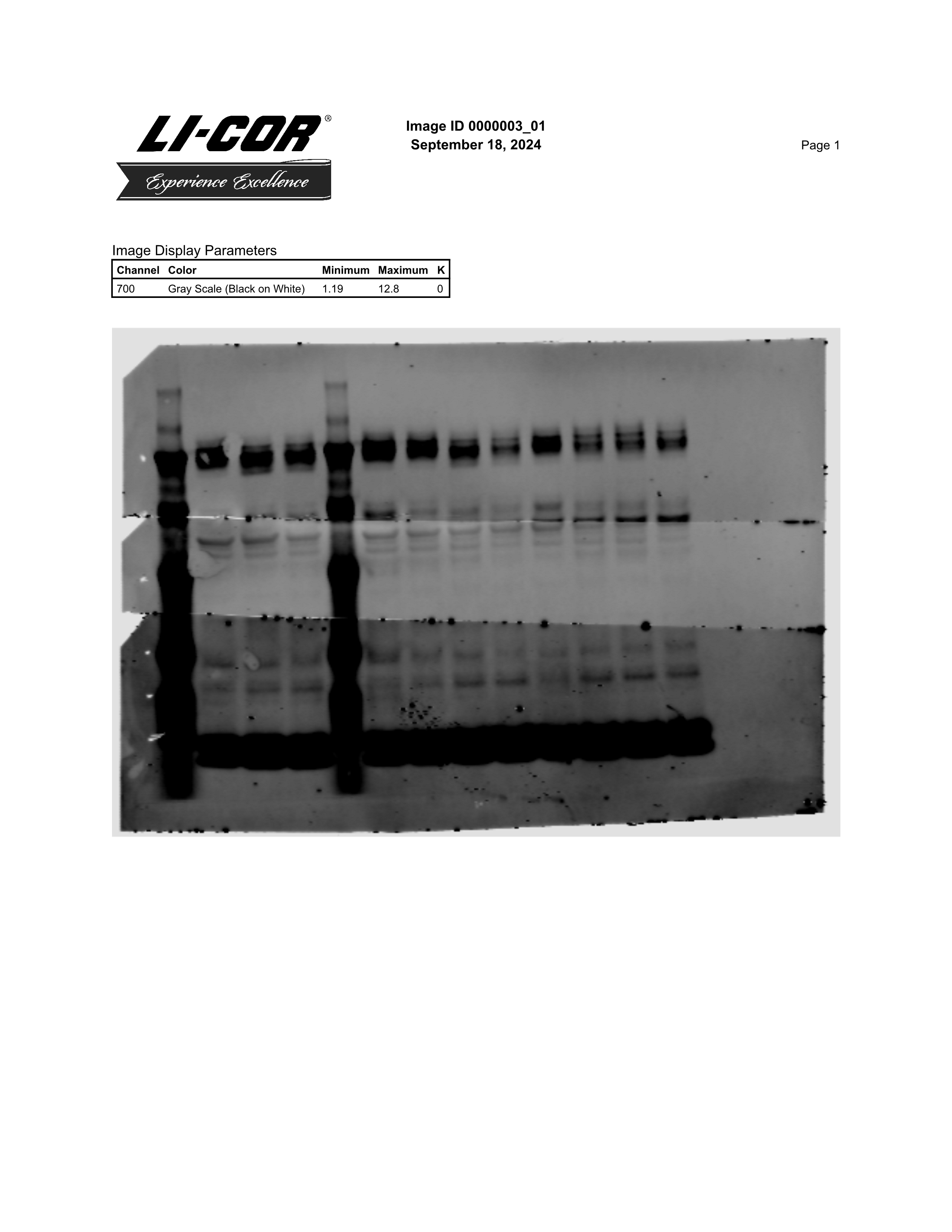

Supplement: Figure 5—source data 1. [file elife-109518-fig5-data1.zip › Figure 5-source data 1/Gel2 3 (Red)_Figure 5J_1.tif]

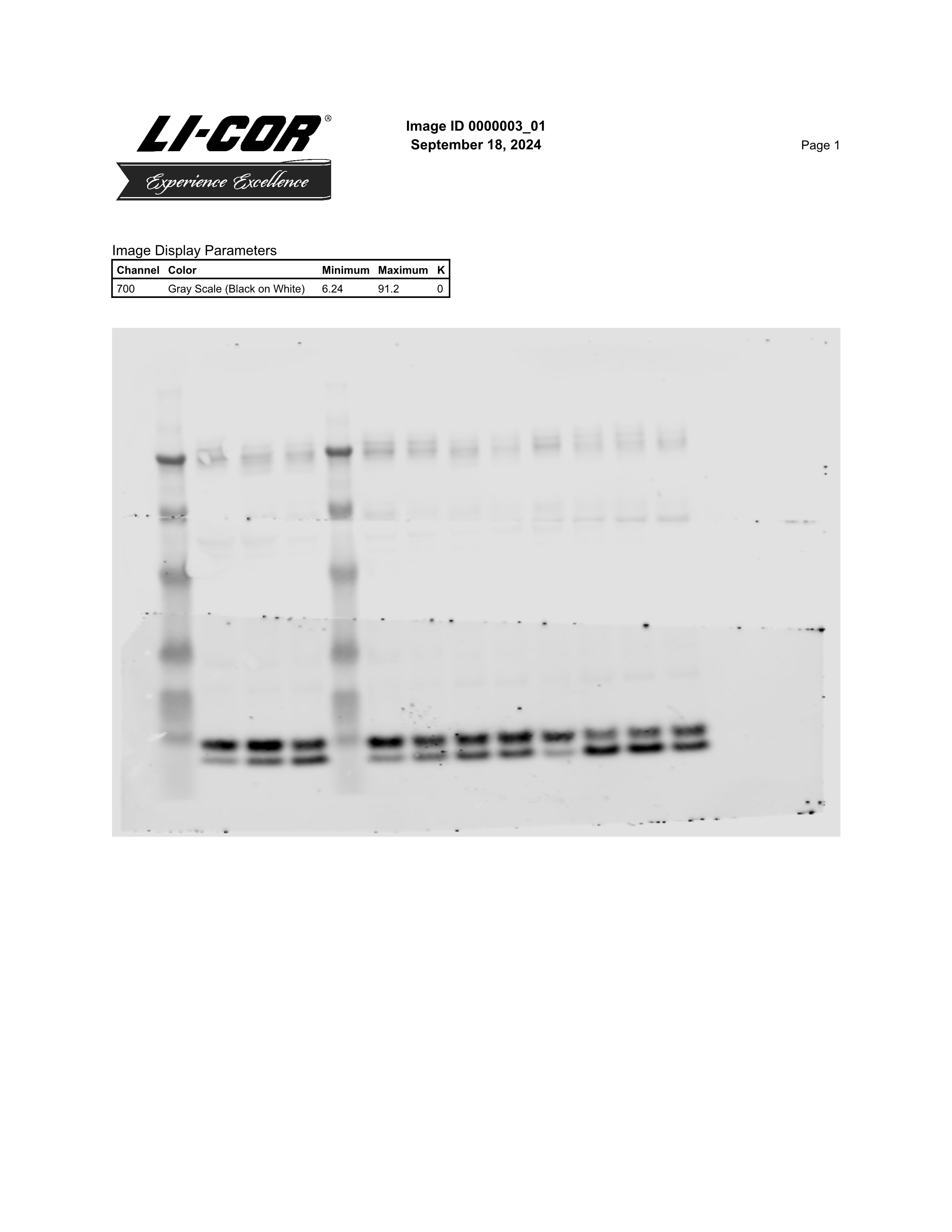

Supplement: Figure 5—source data 1. [file elife-109518-fig5-data1.zip › Figure 5-source data 1/Gel2 4 (Red)_Figure 5J_1.tif]

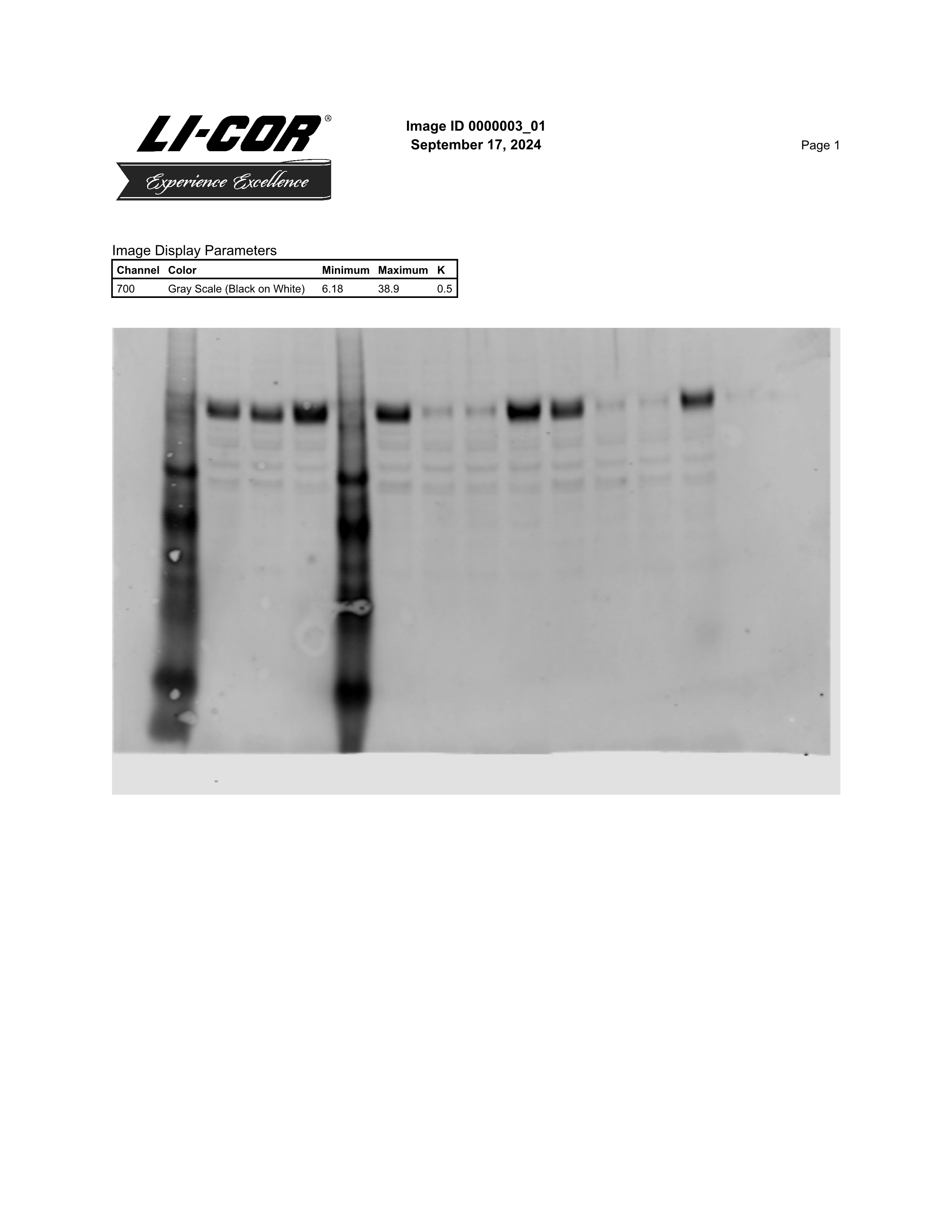

Supplement: Figure 5—source data 1. [file elife-109518-fig5-data1.zip › Figure 5-source data 1/Gel2 hGCase (R) + GAPDH (G) 2_Figure 5E and 5F_1.tif]

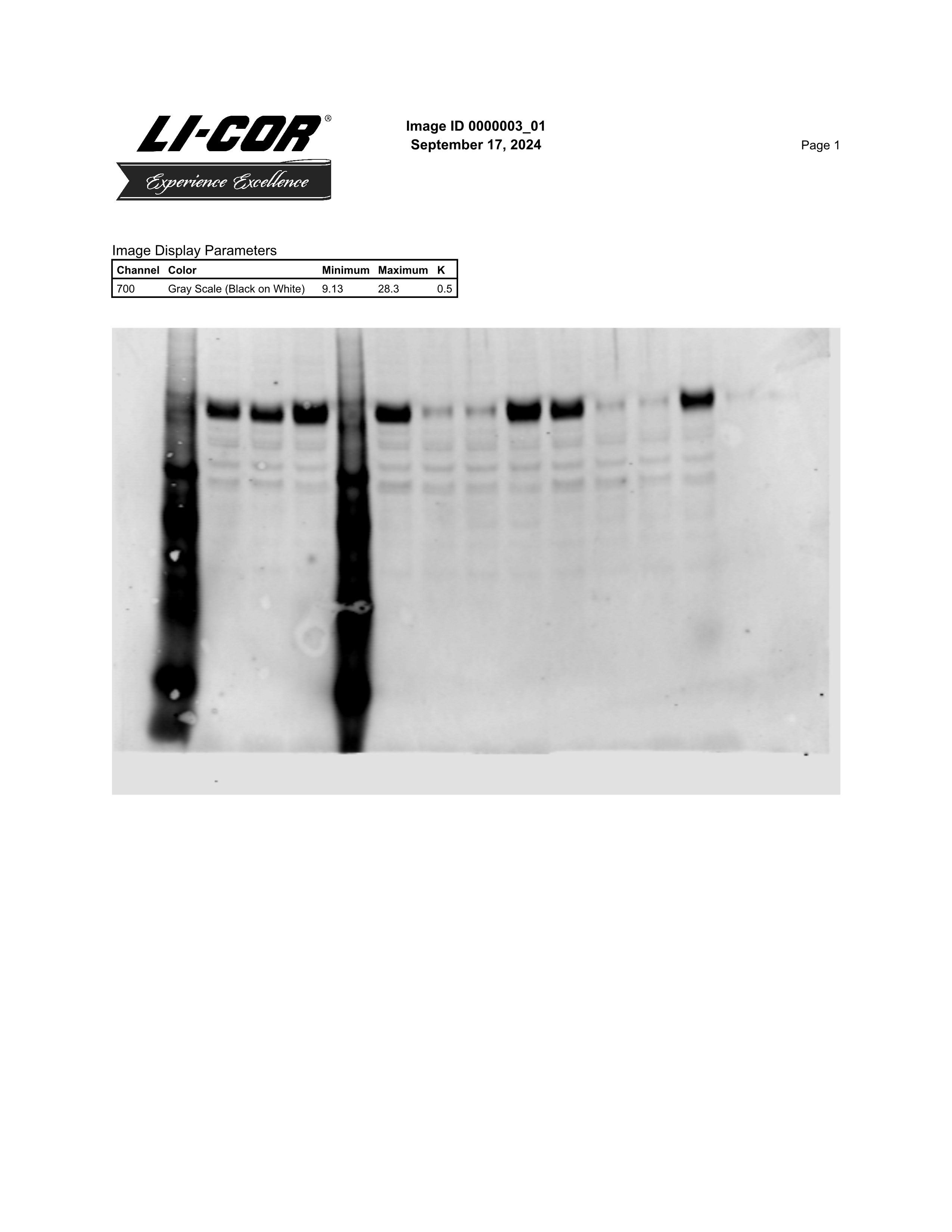

Supplement: Figure 5—source data 1. [file elife-109518-fig5-data1.zip › Figure 5-source data 1/Gel2 hGCase (R) + GAPDH (G) 4_Figure 5G_1.tif]

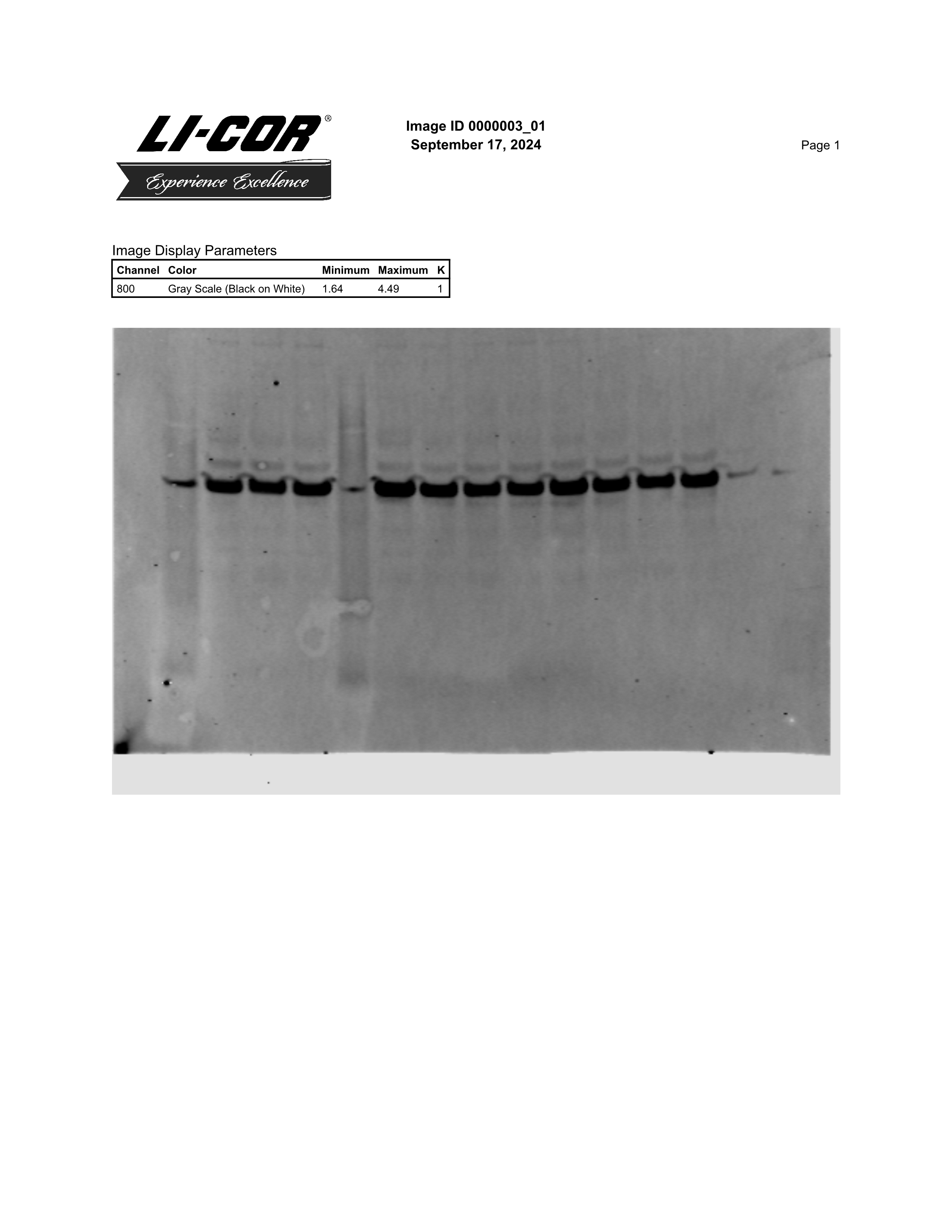

Supplement: Figure 5—source data 1. [file elife-109518-fig5-data1.zip › Figure 5-source data 1/Gel2 hGCase (R) + GAPDH (G) 5_Figure 5E, 5F and 5G_1.tif]

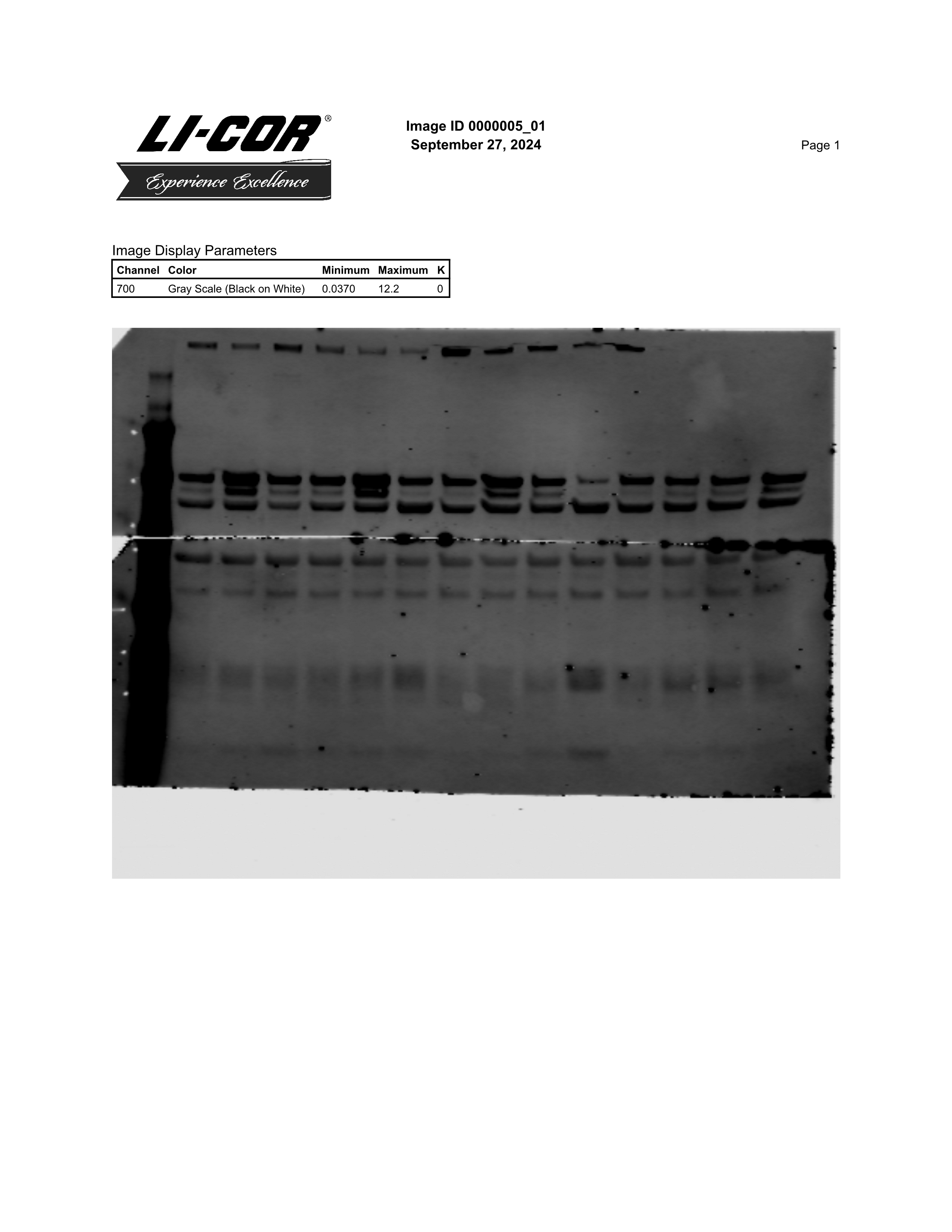

Supplement: Figure 5—source data 1. [file elife-109518-fig5-data1.zip › Figure 5-source data 1/Gel2 TH+p-4EBP2_Figure 5J_1.tif]

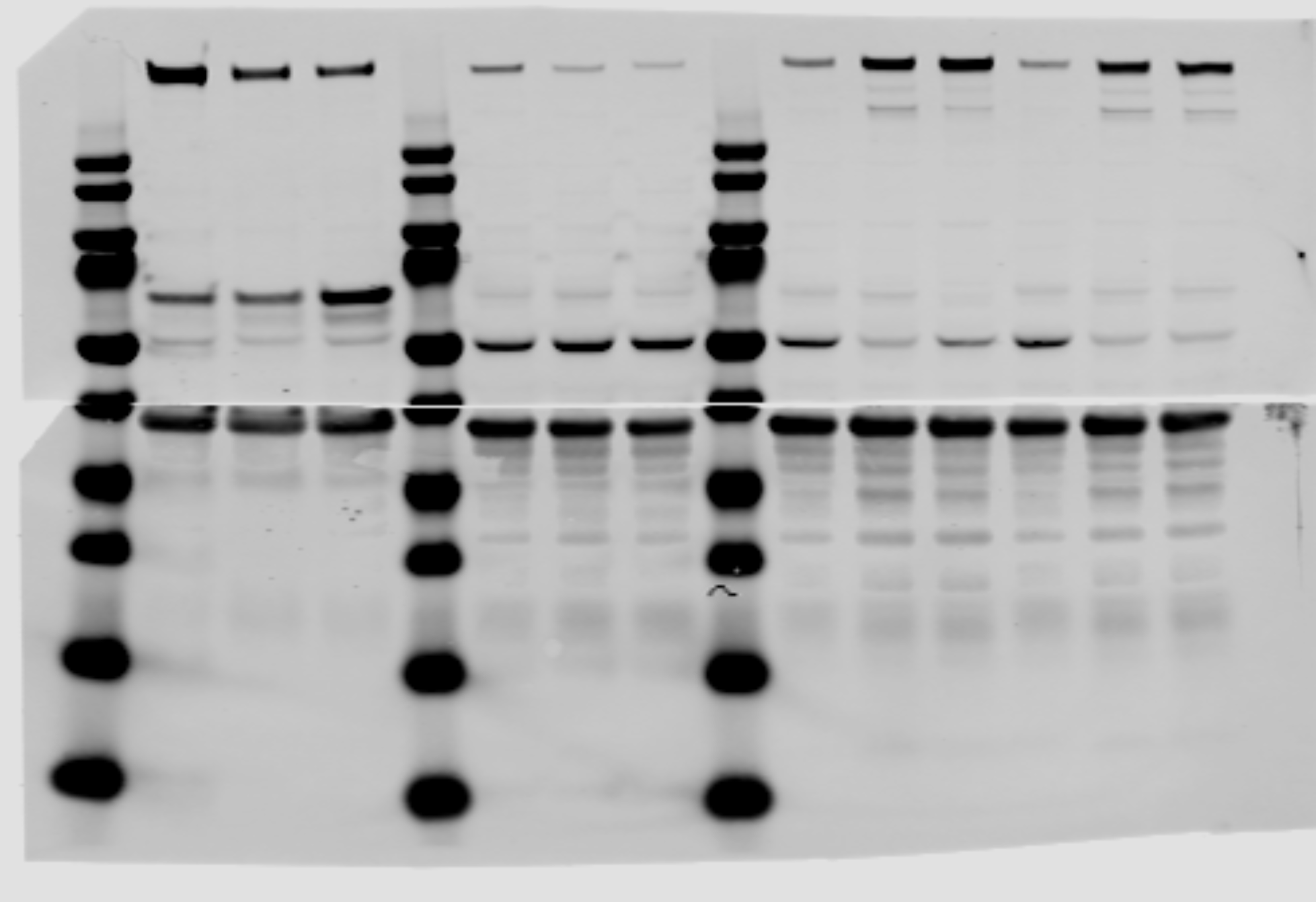

Supplement: Figure 5—figure supplement 1—source data 1. [file elife-109518-fig5-figsupp1-data1.zip › Figure 5-figure supplement 1-source data 1/20250516 Gel 2 TH and GAPDH_5E_28WK.tif]

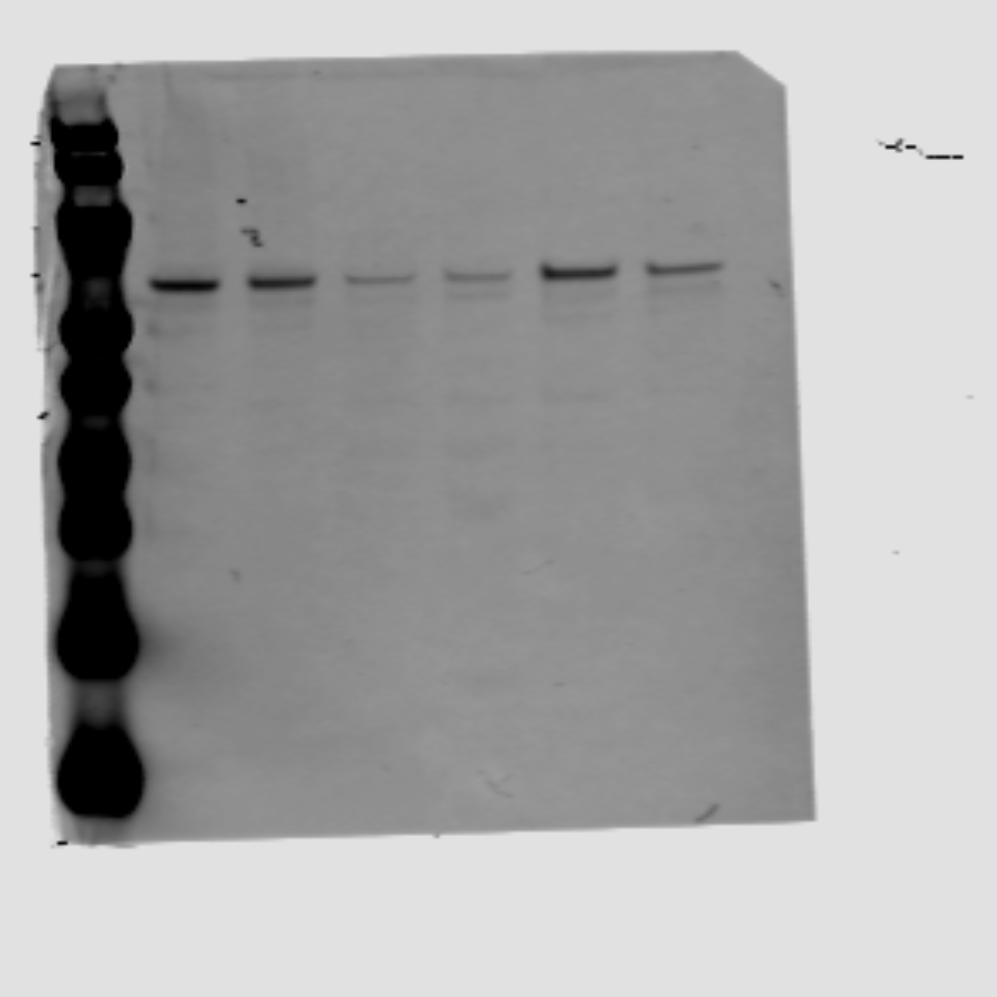

Supplement: Figure 5—figure supplement 1—source data 1. [file elife-109518-fig5-figsupp1-data1.zip › Figure 5-figure supplement 1-source data 1/TH MLO_5E_16WK.tif]

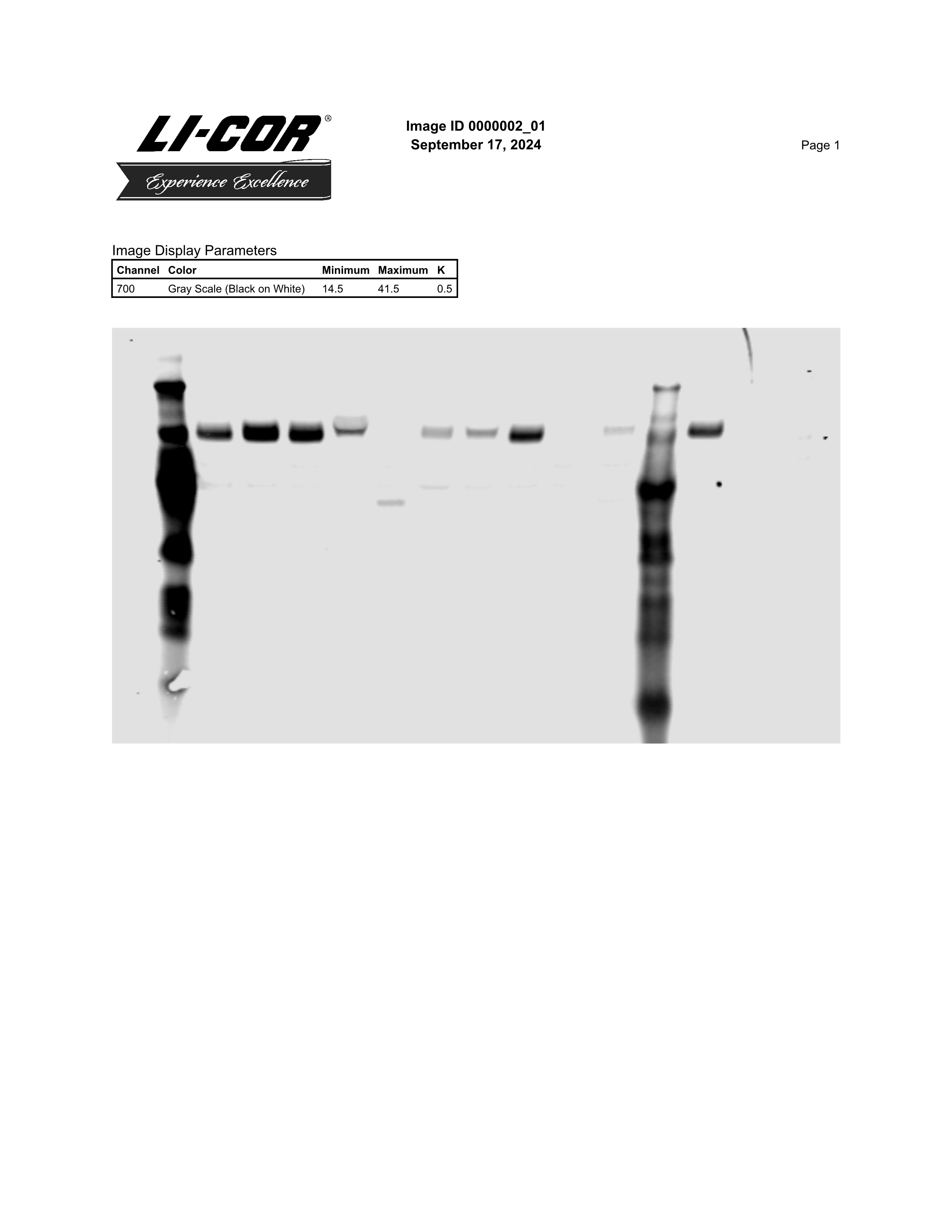

Supplement: Figure 6—source data 1. [file elife-109518-fig6-data1.zip › Figure 6-source data 1/Gel1 hGCase (R) + GAPDH (G) 2-2_1.tif]

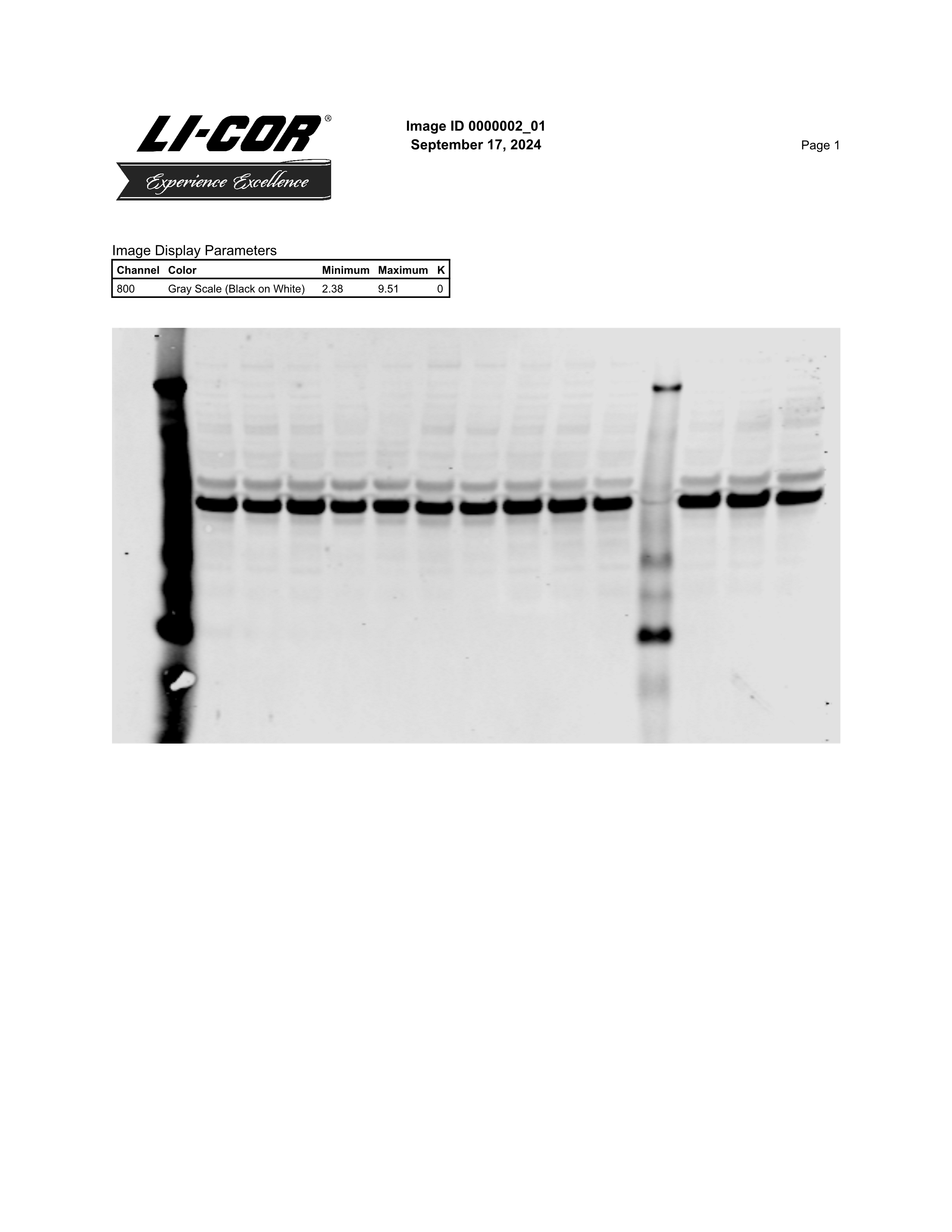

Supplement: Figure 6—source data 1. [file elife-109518-fig6-data1.zip › Figure 6-source data 1/Gel1 hGCase (R) + GAPDH (G) 5-2_1.tif]

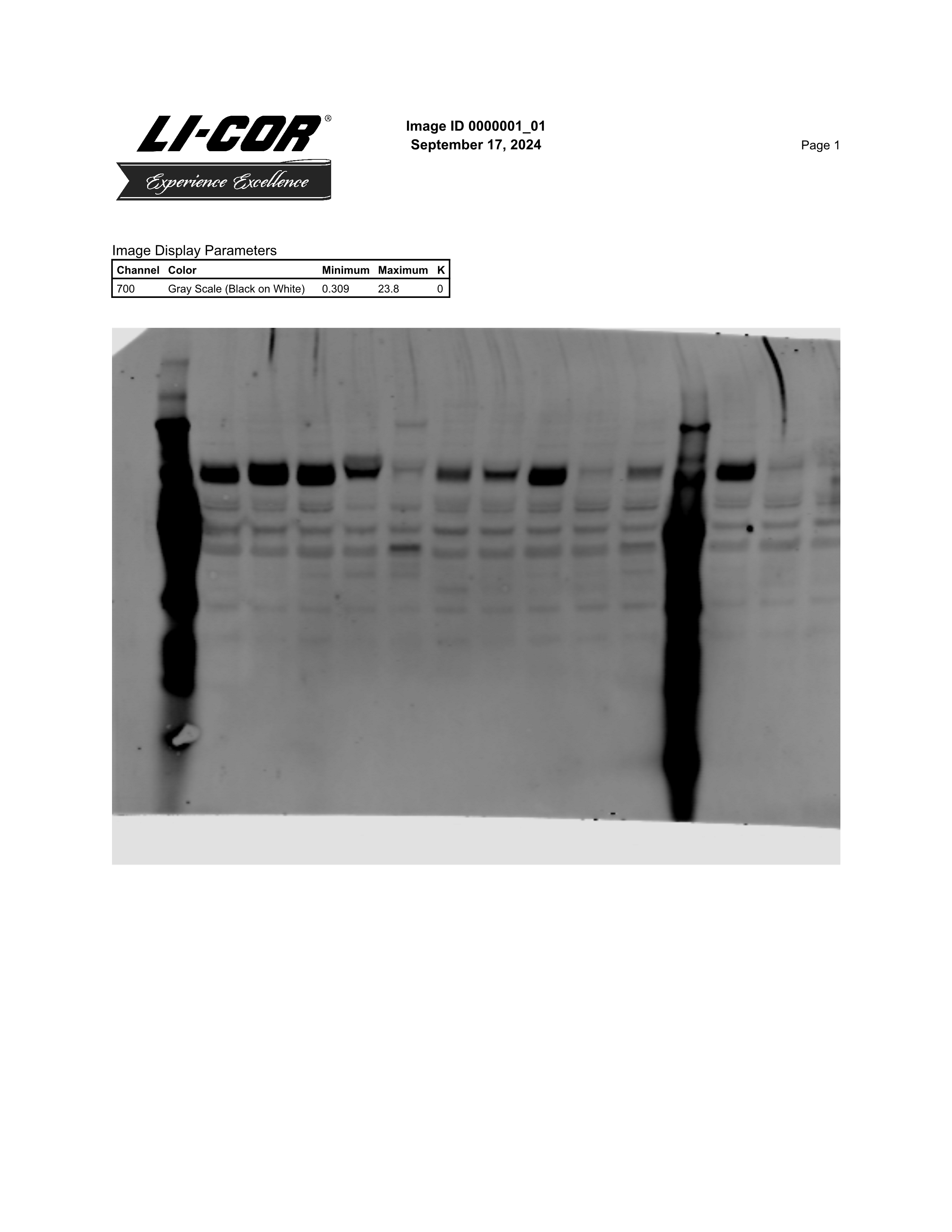

Supplement: Figure 6—source data 1. [file elife-109518-fig6-data1.zip › Figure 6-source data 1/Gel1 hGCase (R) + GAPDH (G) 5_1.tif]

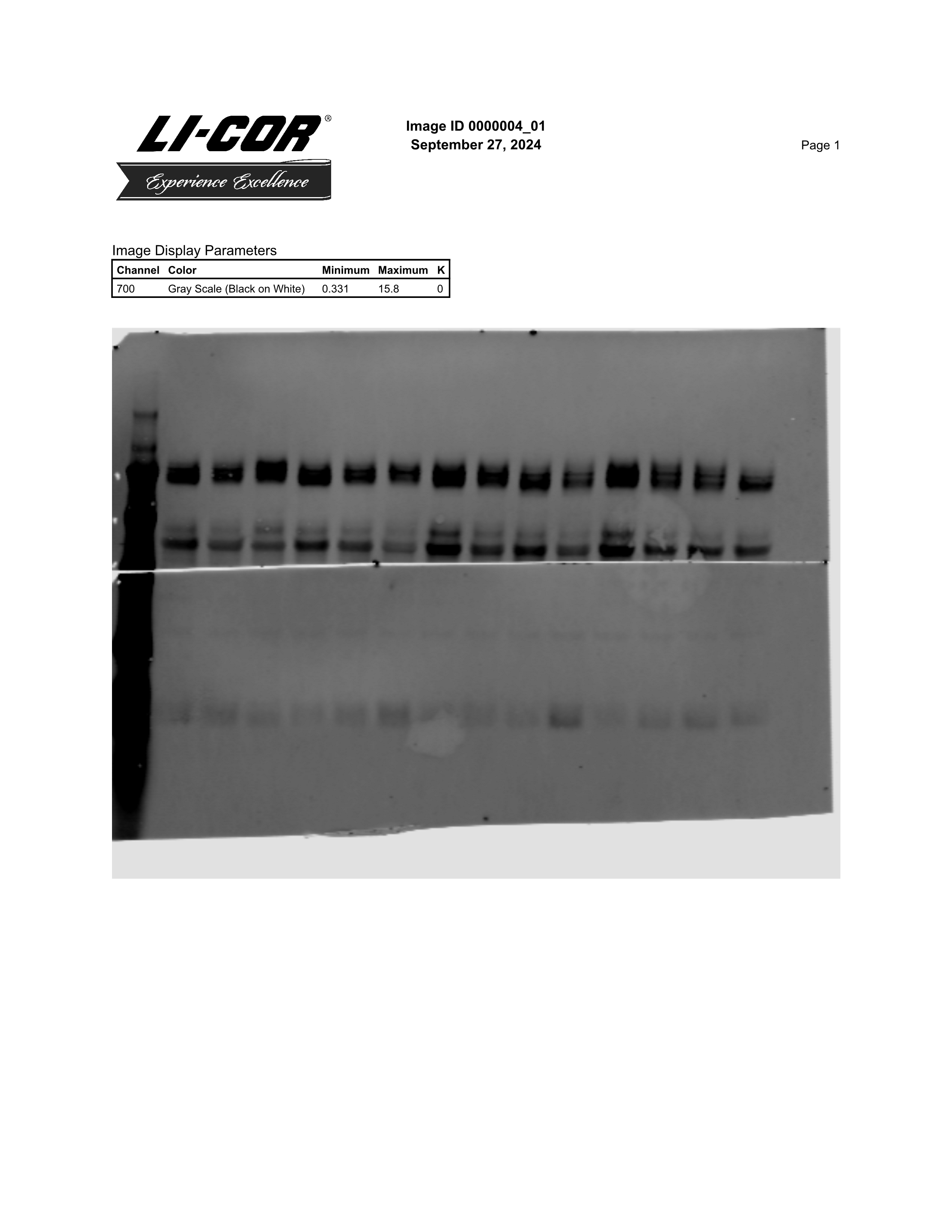

Supplement: Figure 6—source data 1. [file elife-109518-fig6-data1.zip › Figure 6-source data 1/Gel1 LAMP1 (R) + GFAP (G) 3_1.tif]

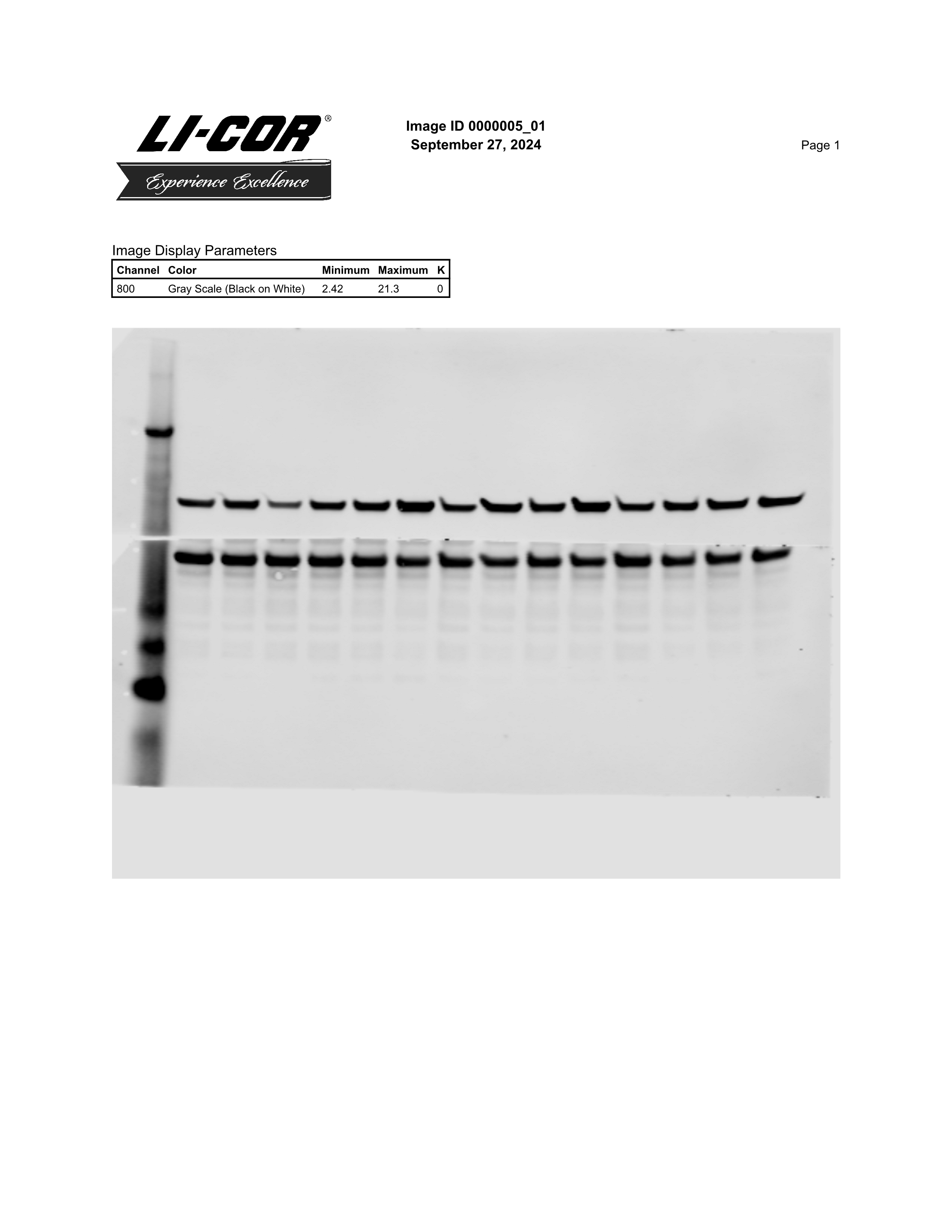

Supplement: Figure 6—source data 1. [file elife-109518-fig6-data1.zip › Figure 6-source data 1/Gel2 4_TH_1.tif]

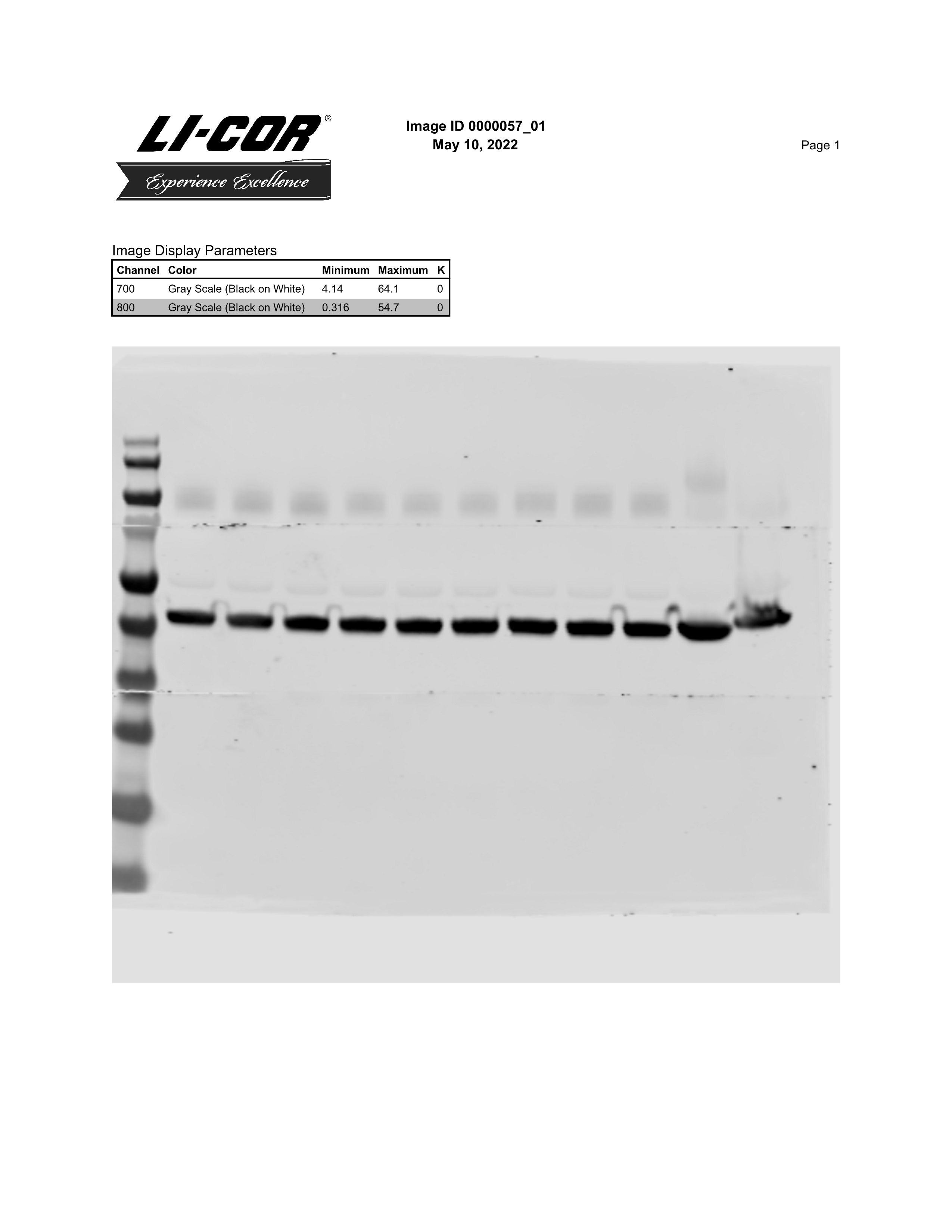

Supplement: Figure 7—source data 1. [file elife-109518-fig7-data1.zip › Figure 7-source data 1/Gel 1-2_Actin_1.tif]

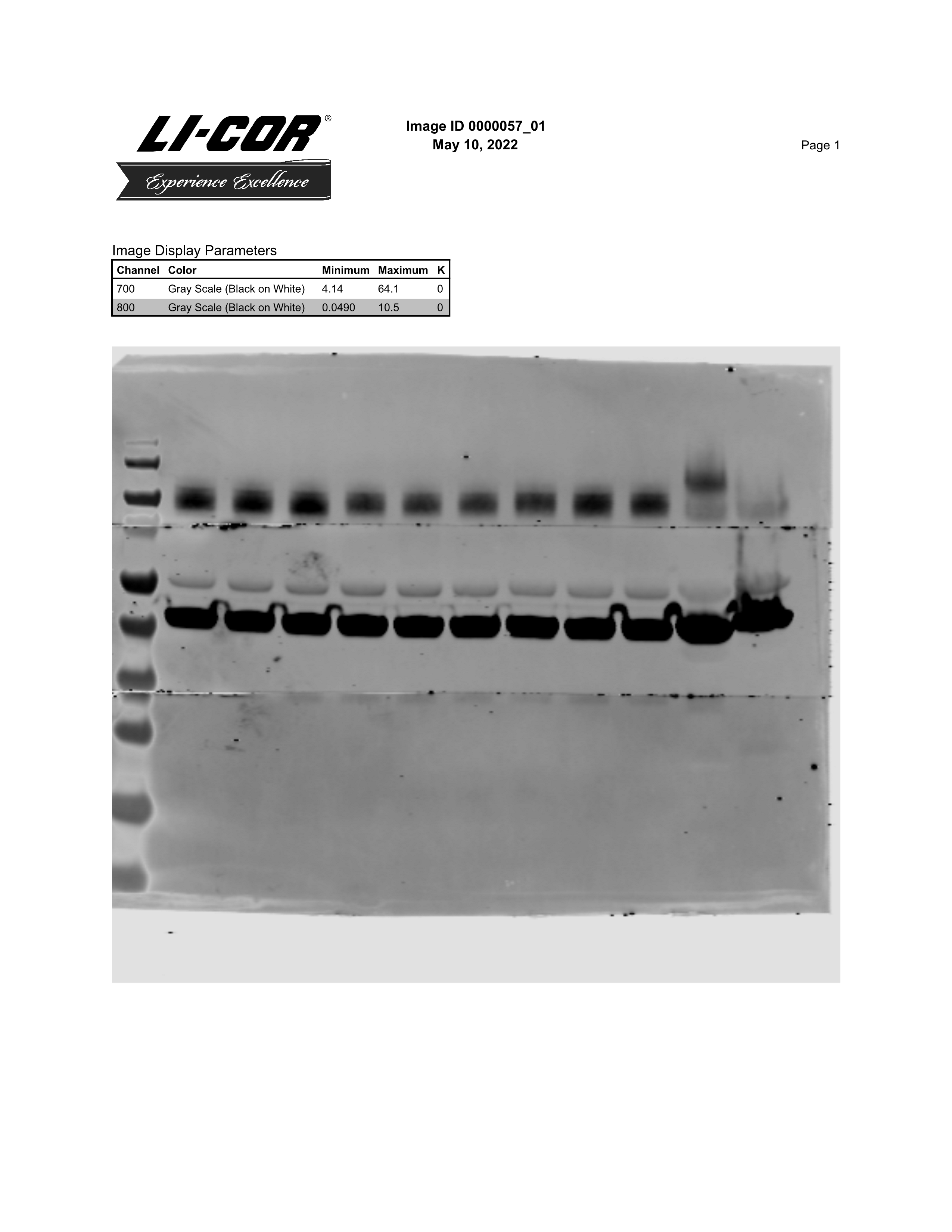

Supplement: Figure 7—source data 1. [file elife-109518-fig7-data1.zip › Figure 7-source data 1/Gel 1-4_LAMP1_1.tif]

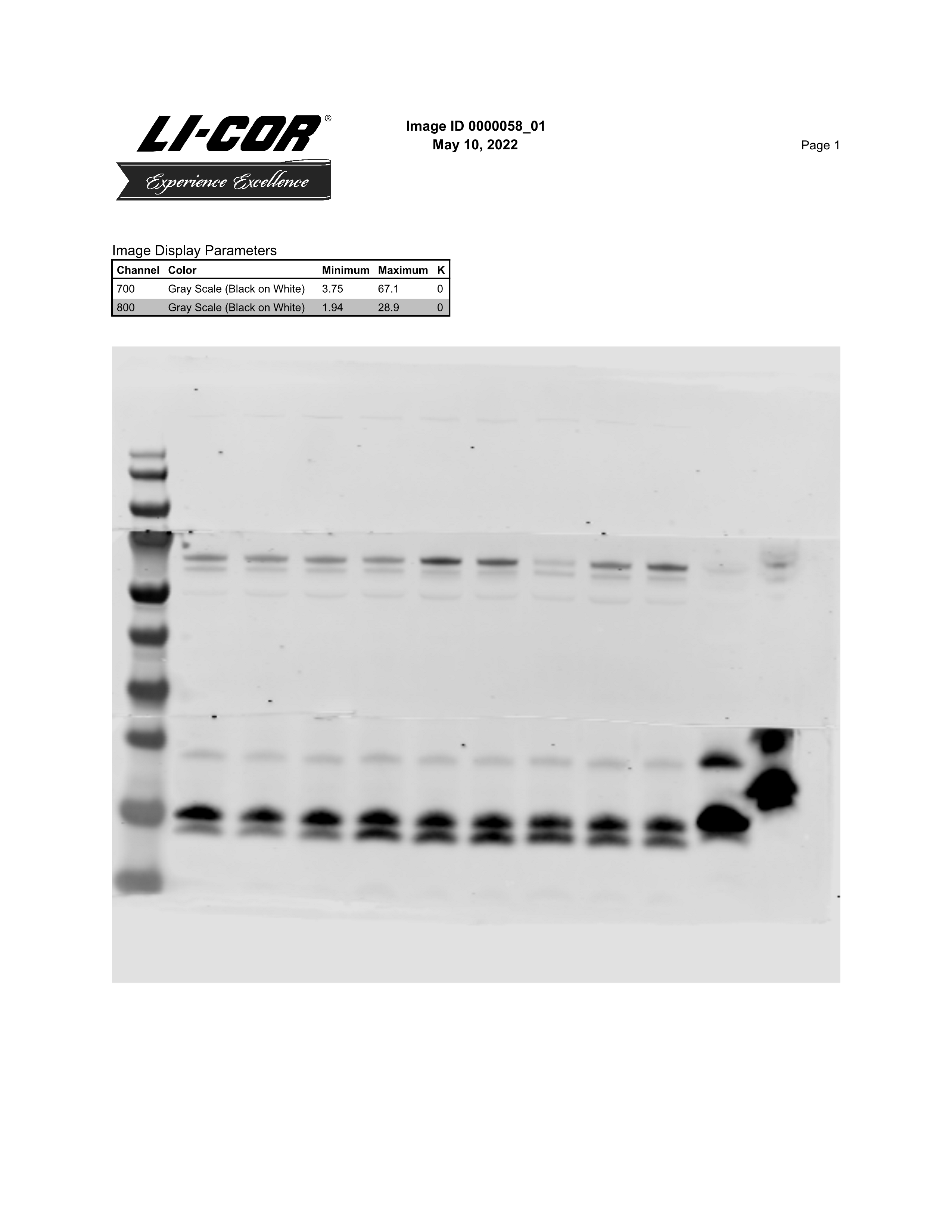

Supplement: Figure 7—source data 1. [file elife-109518-fig7-data1.zip › Figure 7-source data 1/Gel 2-5_LC3_1.tif]
