# Supplementary material for: Patient-specific midbrain organoids with CRISPR correction recapitulate neuronopathic Gaucher disease phenotypes and enable evaluation of novel therapies
Source: eLife. 2026 Jun 23;15:RP109518. doi: 10.7554/eLife.109518 (PMC13290227; doi:10.7554/eLife.109518)
Supplement: Figure 1—source data 2. [file elife-109518-fig1-data2.zip › Figure 1-source data 2.pdf]

Figure 1-source data 2
Figure 1E

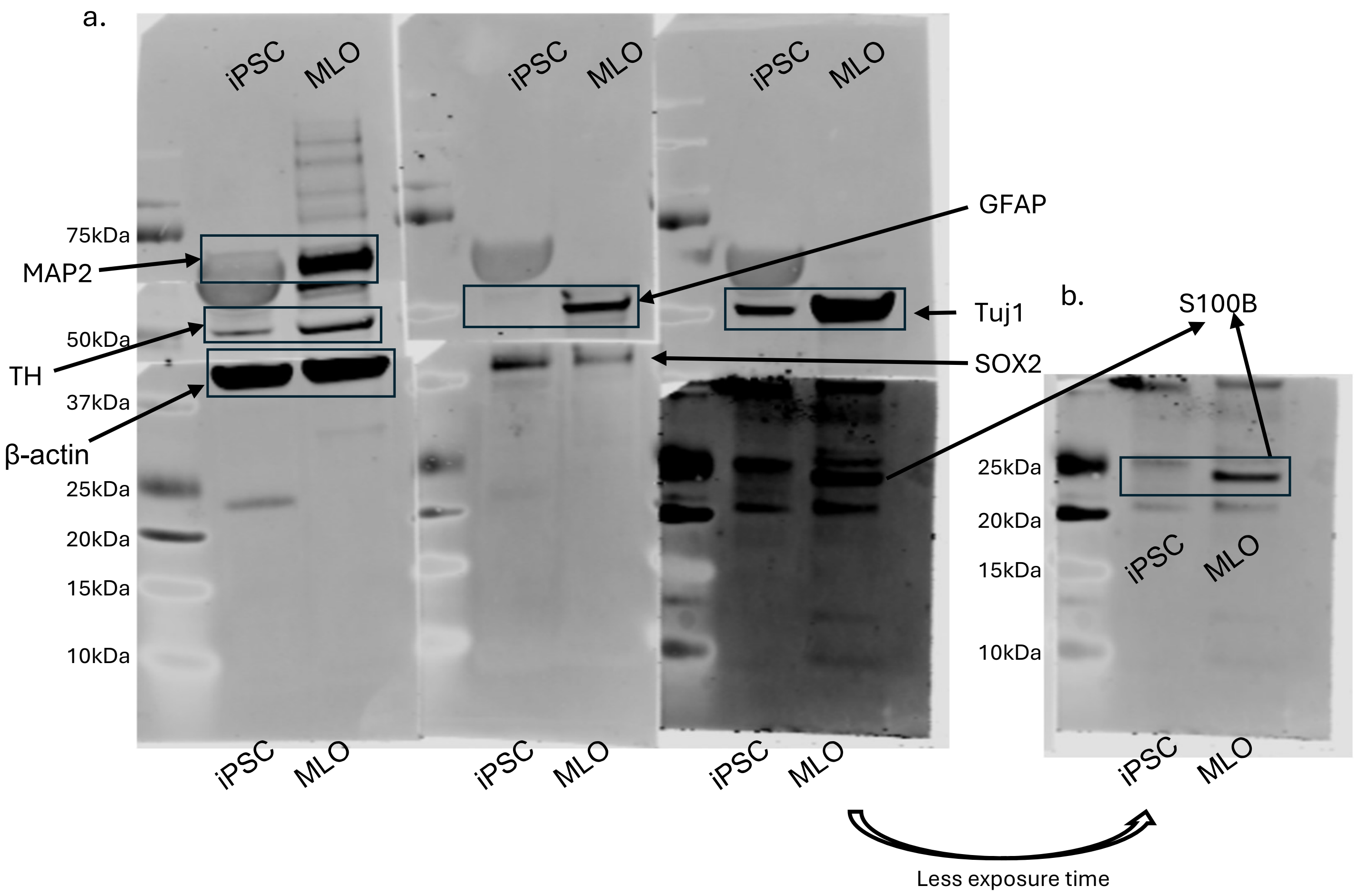

Figure 1-Source Data 2. Original membranes corresponding to Figure 1, panel E.
Panel a: Original blots for SOX2, Tuj1, MAP2, GFAP, S100B and β-actin. Precision Plus Protein Dual Color Standards were used. Panel b: Image with less exposure time is shown for clear S100B band used in Figure 1E.
