# Supplementary material for: Patient-specific midbrain organoids with CRISPR correction recapitulate neuronopathic Gaucher disease phenotypes and enable evaluation of novel therapies
Source: eLife. 2026 Jun 23;15:RP109518. doi: 10.7554/eLife.109518 (PMC13290227; doi:10.7554/eLife.109518)
Supplement: Figure 2—source data 2. [file elife-109518-fig2-data2.zip › Figure 2-source data 2.pdf]

Figure 2-source data 2
Figure 2A

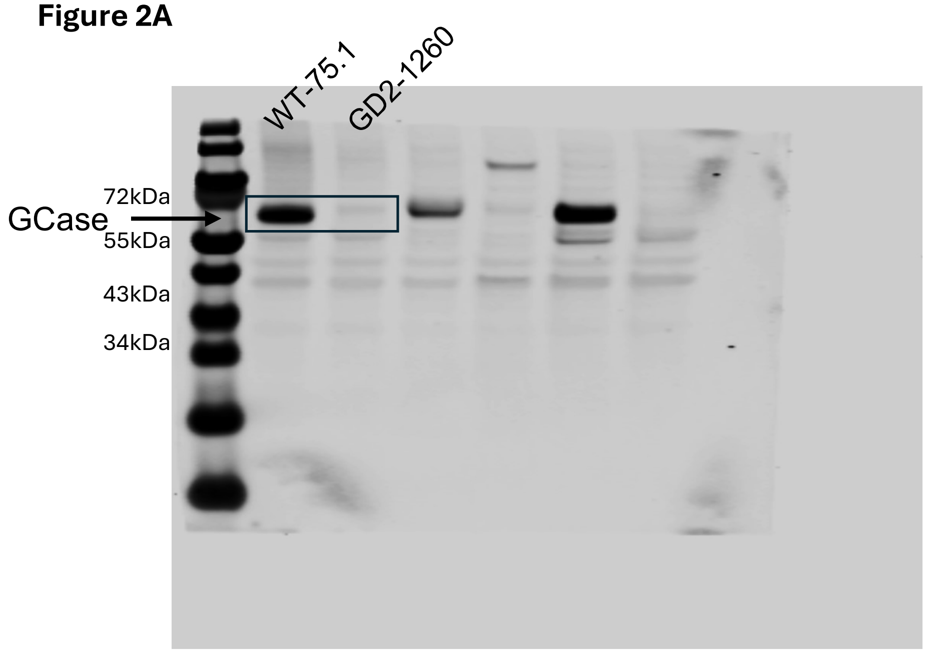

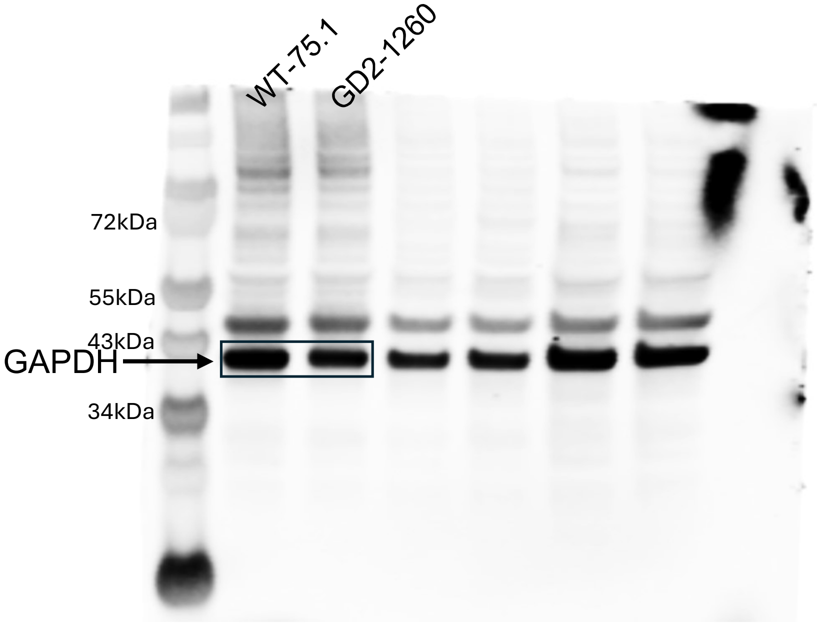

Figure 2-source data 2. Original membranes corresponding to Figure 2, panel A.

EZ-Run™ Prestained Rec Protein Ladder was used. The other lanes correspond to other tested samples (lanes 4-7) are not shown in Figure 2.
