# Supplementary material for: Patient-specific midbrain organoids with CRISPR correction recapitulate neuronopathic Gaucher disease phenotypes and enable evaluation of novel therapies
Source: eLife. 2026 Jun 23;15:RP109518. doi: 10.7554/eLife.109518 (PMC13290227; doi:10.7554/eLife.109518)
Supplement: Figure 3—source data 2. [file elife-109518-fig3-data2.zip › Figure 3-source data 2.pdf]

Figure 3-source data 2

Figure 3G

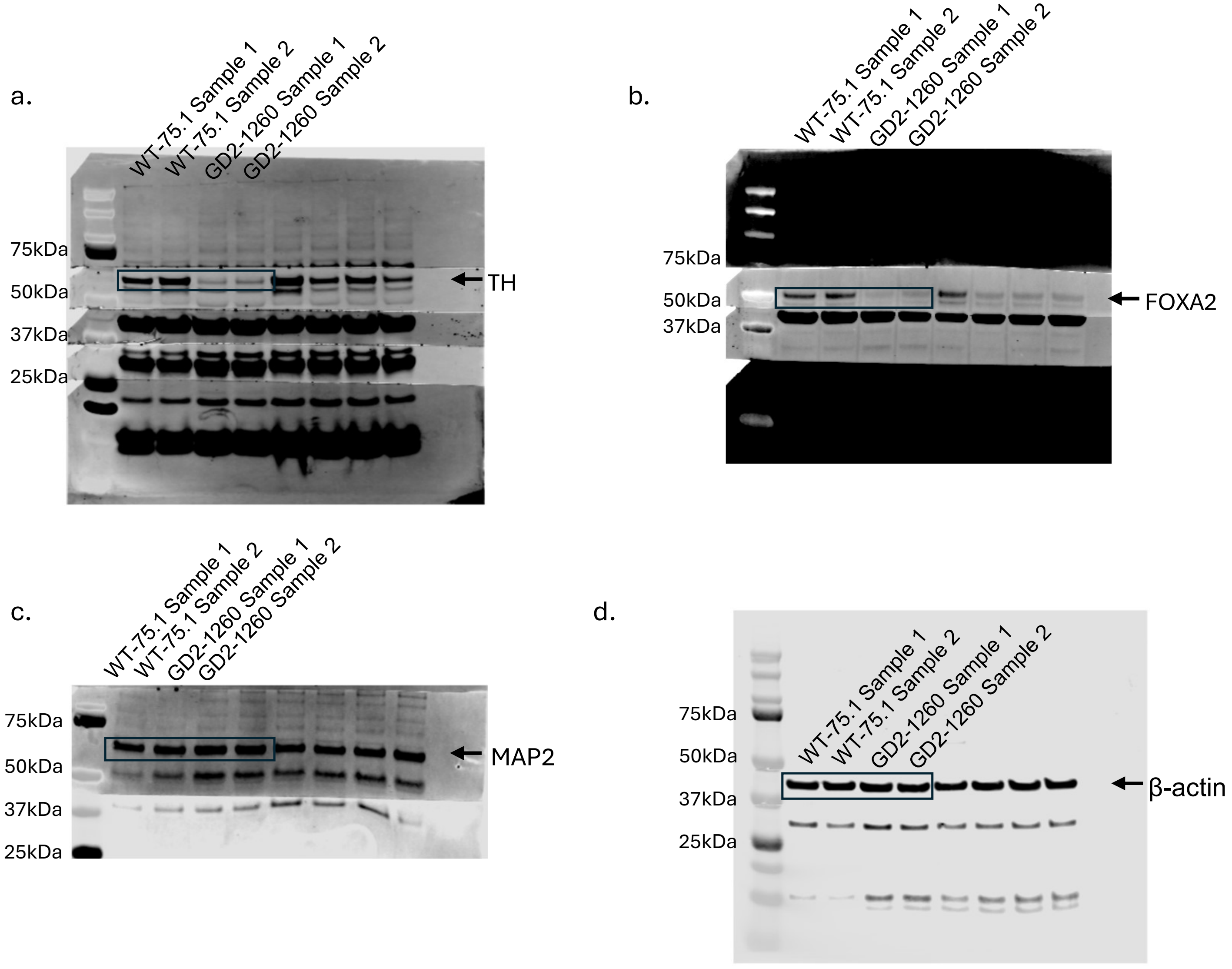

Figure 3-Source Data 2. Original membranes corresponding to Figure 3, panel G.
Panel a: Original blots for TH. First four lanes are WT-75.1 and GD2-1260 samples with 2 biological replicates for each.
Panel b: Original blots for FOXA2. First four lanes are WT-75.1 and GD2-1260 samples with 2 biological replicates for each.
Panel c: Original blots for MAP2. First four lanes are WT-75.1 and GD2-1260 samples with 2 biological replicates for each.
Panel d: Original blots for β-actin. First four lanes are WT-75.1 and GD2-1260 samples with 2 biological replicates for each.
Precision Plus Protein Dual Color Standards were used. Lanes 6, 7, 8 and 9. along with other bands on membranes corresponding to other tested targets are not shown in Figure 3G.
