# Supplementary material for: Patient-specific midbrain organoids with CRISPR correction recapitulate neuronopathic Gaucher disease phenotypes and enable evaluation of novel therapies
Source: eLife. 2026 Jun 23;15:RP109518. doi: 10.7554/eLife.109518 (PMC13290227; doi:10.7554/eLife.109518)
Supplement: Figure 4—source data 2. [file elife-109518-fig4-data2.zip › Figure 4-source data 2.pdf]

Figure 4-source data 2
Figure 4B

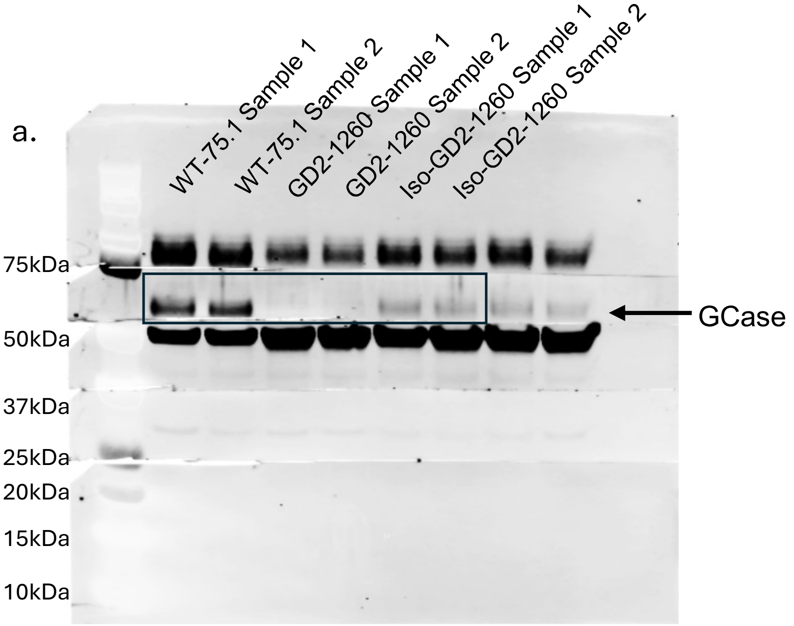

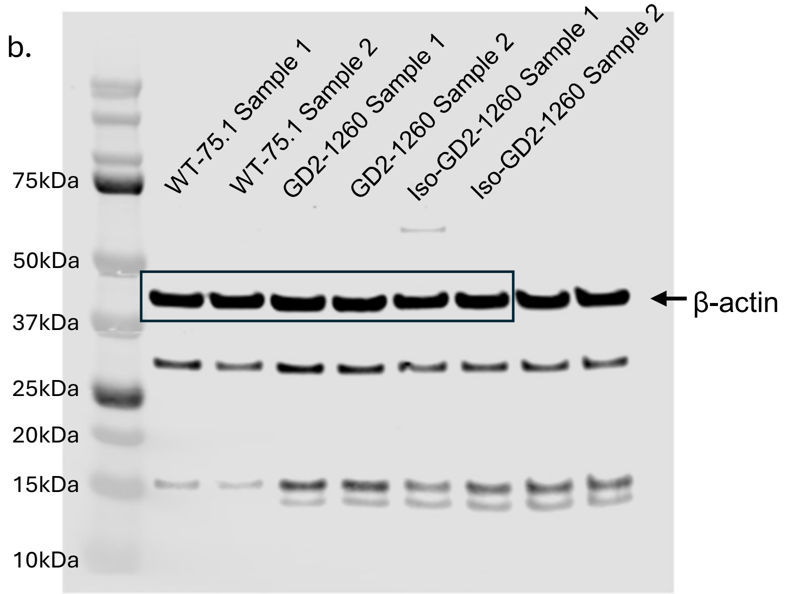

Figure 4-Source Data 2_4B. Original membranes corresponding to Figure 4, panel B. Original blots for GCase (panel a) and β-actin (panel b). Same volume of samples were loaded in gel2 (panel a) and gel1 (panel b) that were blotted parallelly. Precision Plus Protein Dual Color Standards were used. Lane 8 and 9, along with other bands on membranes corresponding to different tested targets, are not shown in Figure 4.

Figure 4-source data 2
Figure 4H

a.

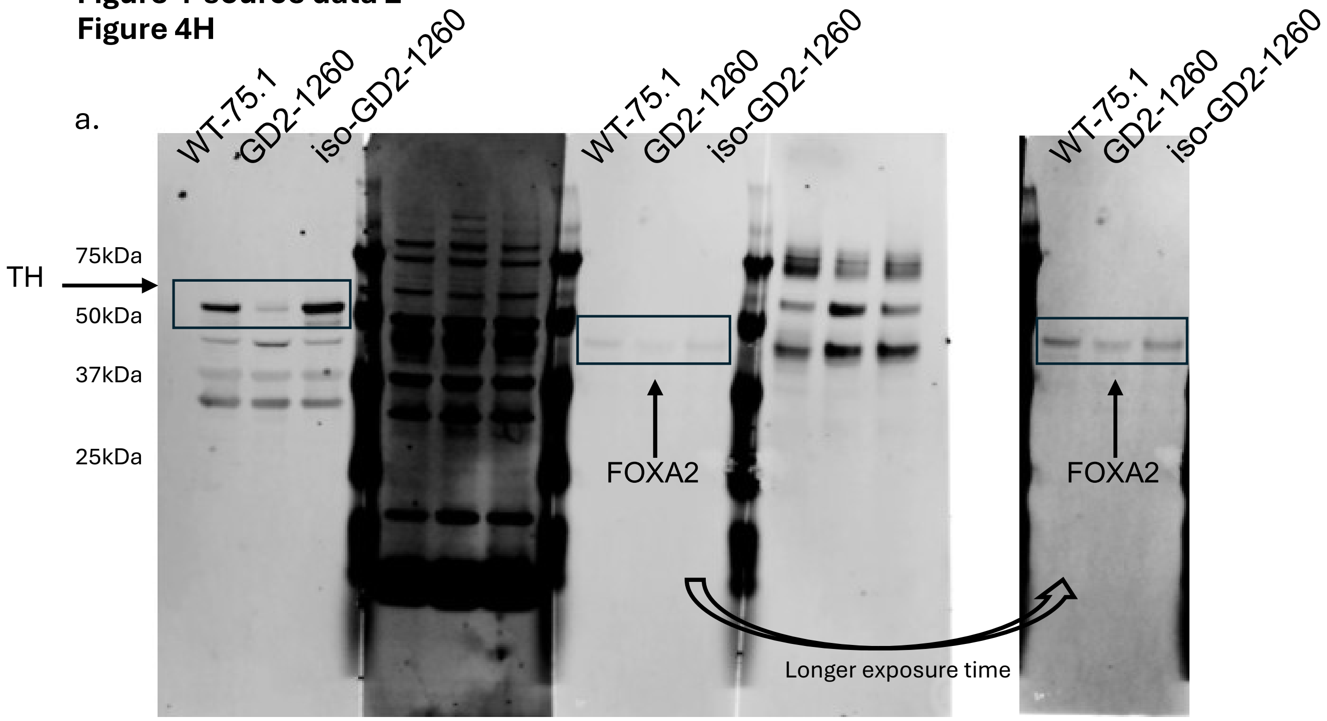

b.

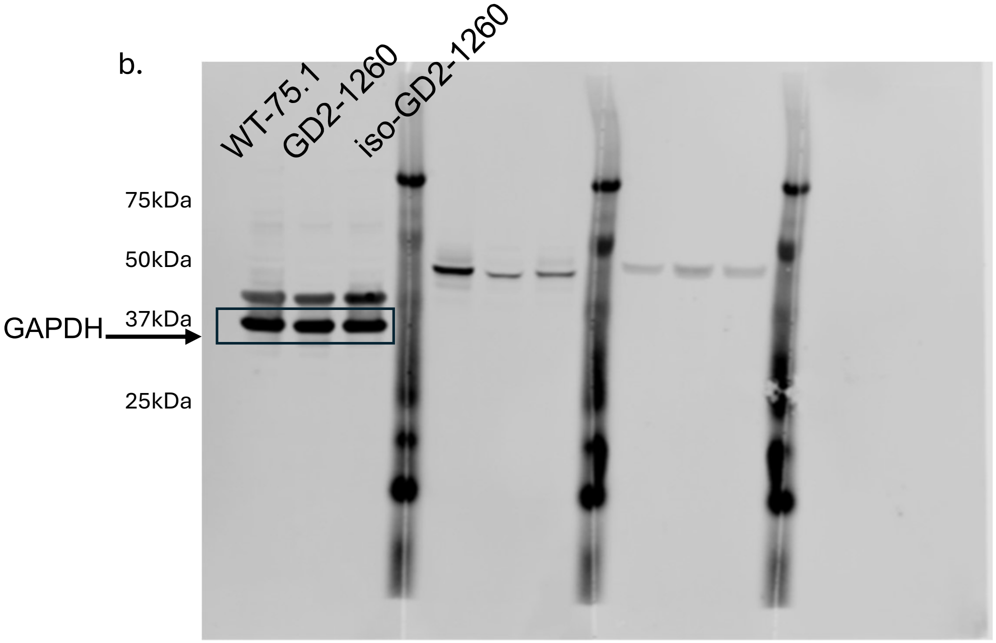

Figure 4-Source Data 2_4H. Original membranes corresponding to Figure 4, panel H. Original blots for TH, FOXA2 and GAPDH. All targets blotted on separate membranes that were from the same gel. Precision Plus Protein Dual Color Standards were used. Other lanes, along with other bands on membranes corresponding to different tested targets, are not shown in Figure 4H.

Figure 4-source data 2
Figure 4K

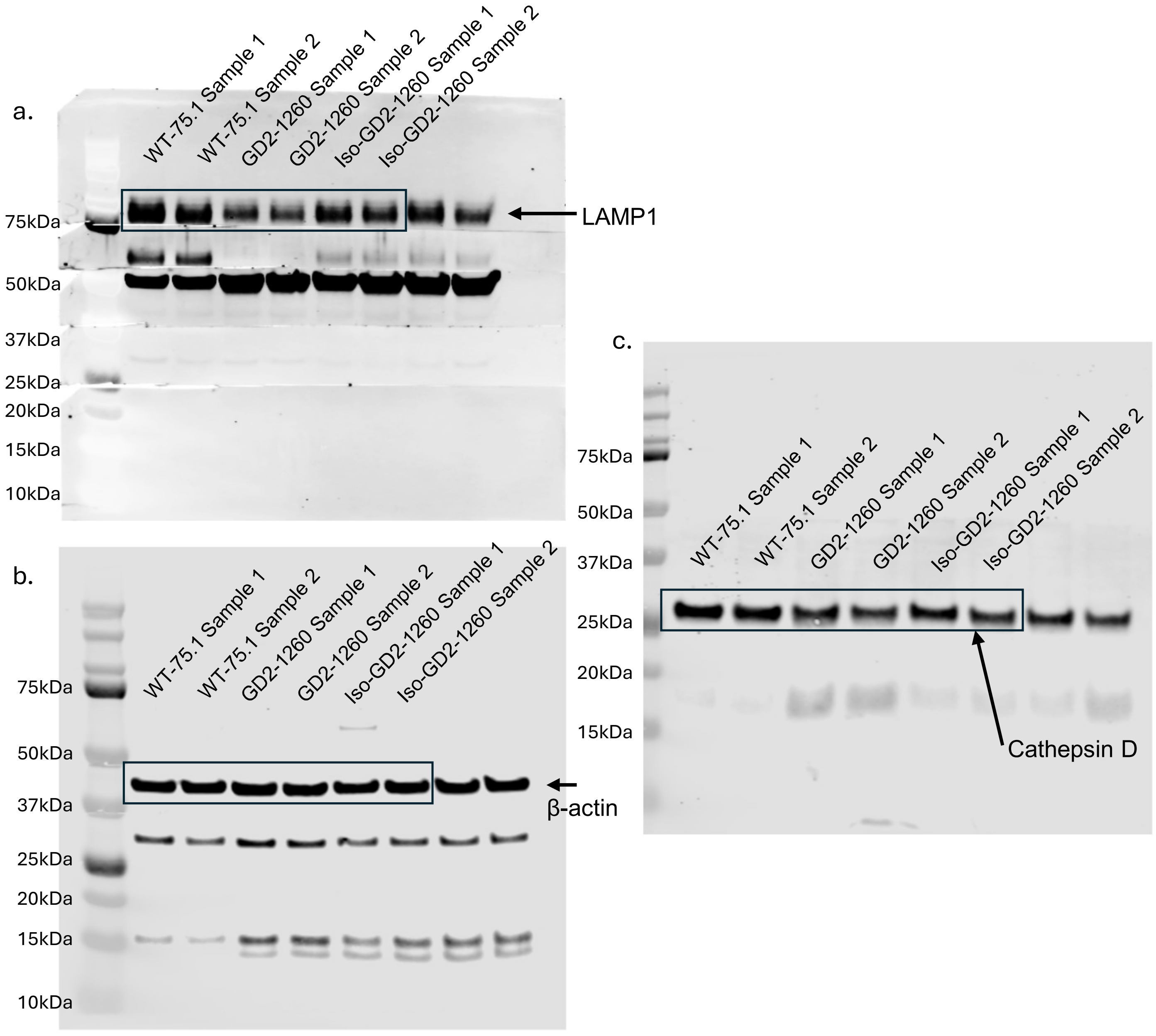

Figure 4-Source Data 2_4K. Original membranes corresponding to Figure 4, panel K. Panels a and b: Original blots for LAMP1 and β-actin (identical to Figure 4-Source Data 1. Same volume of samples were loaded in gel2 (panel a) and gel1 (panel b) that were blotted parallelly. Panel c: Original blots for Cathepsin D. Same volume of samples were loaded as in panel a and b. Precision Plus Protein Dual Color Standards were used. Lane 8 and 9, along with other bands on membranes corresponding to different tested targets, are not shown in Figure 4.

Figure 4-source data 2
Figure 4M

a.

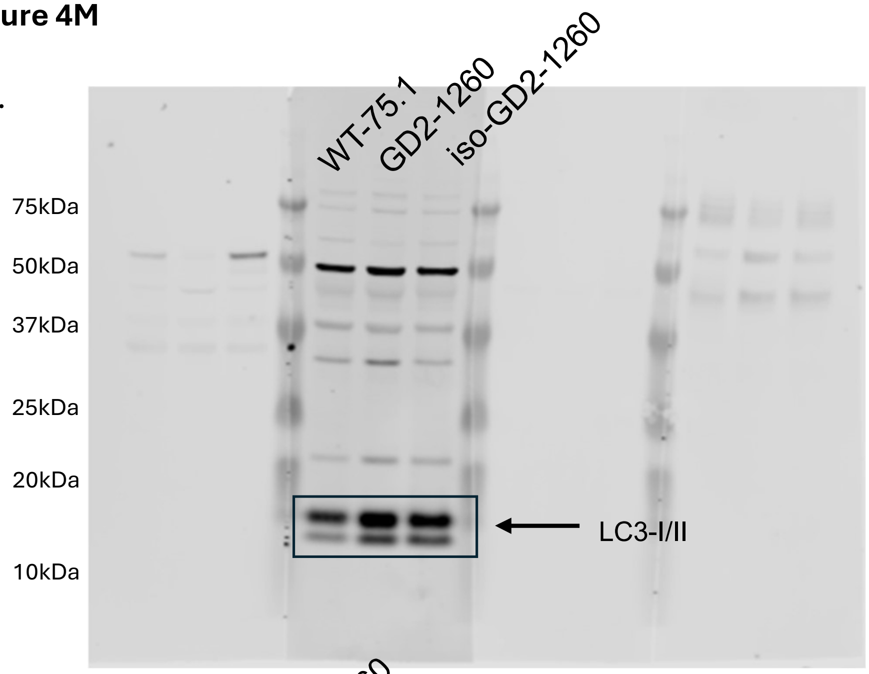

b.

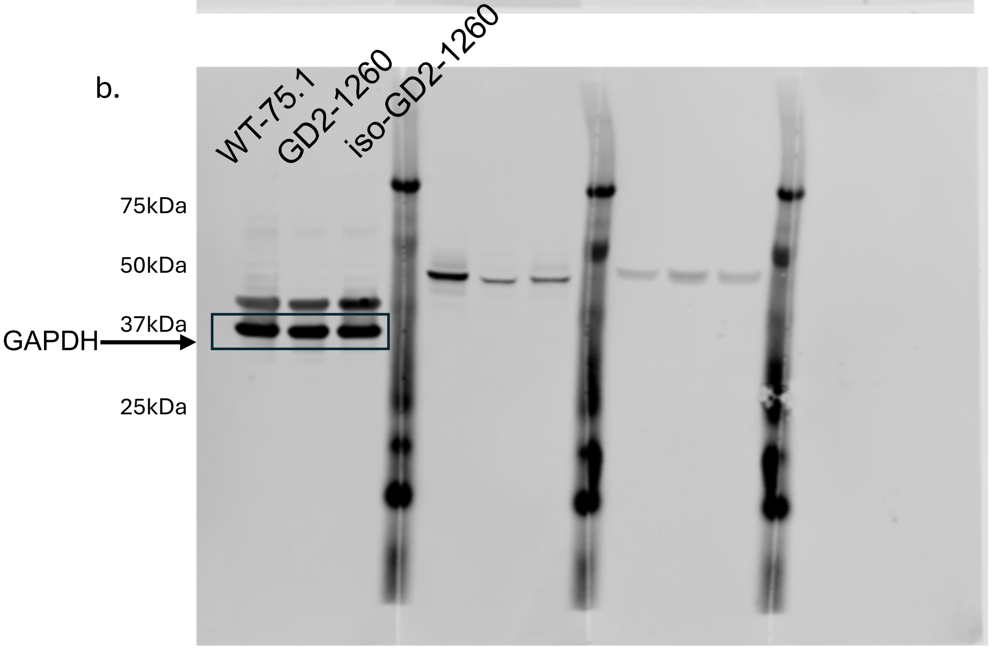

Figure 4-Source Data 2_4M. Original membranes corresponding to Figure 4, panel M. Original blots for LC3-I/II (panel a) and GAPDH (panel b). The same amount of protein from each sample was loaded onto separate gels. Precision Plus Protein Dual Color Standards were used. Other lanes, along with other bands on membranes corresponding to different tested targets, are not shown in Figure 4M.

Figure 4-source data 2
Figure 4O

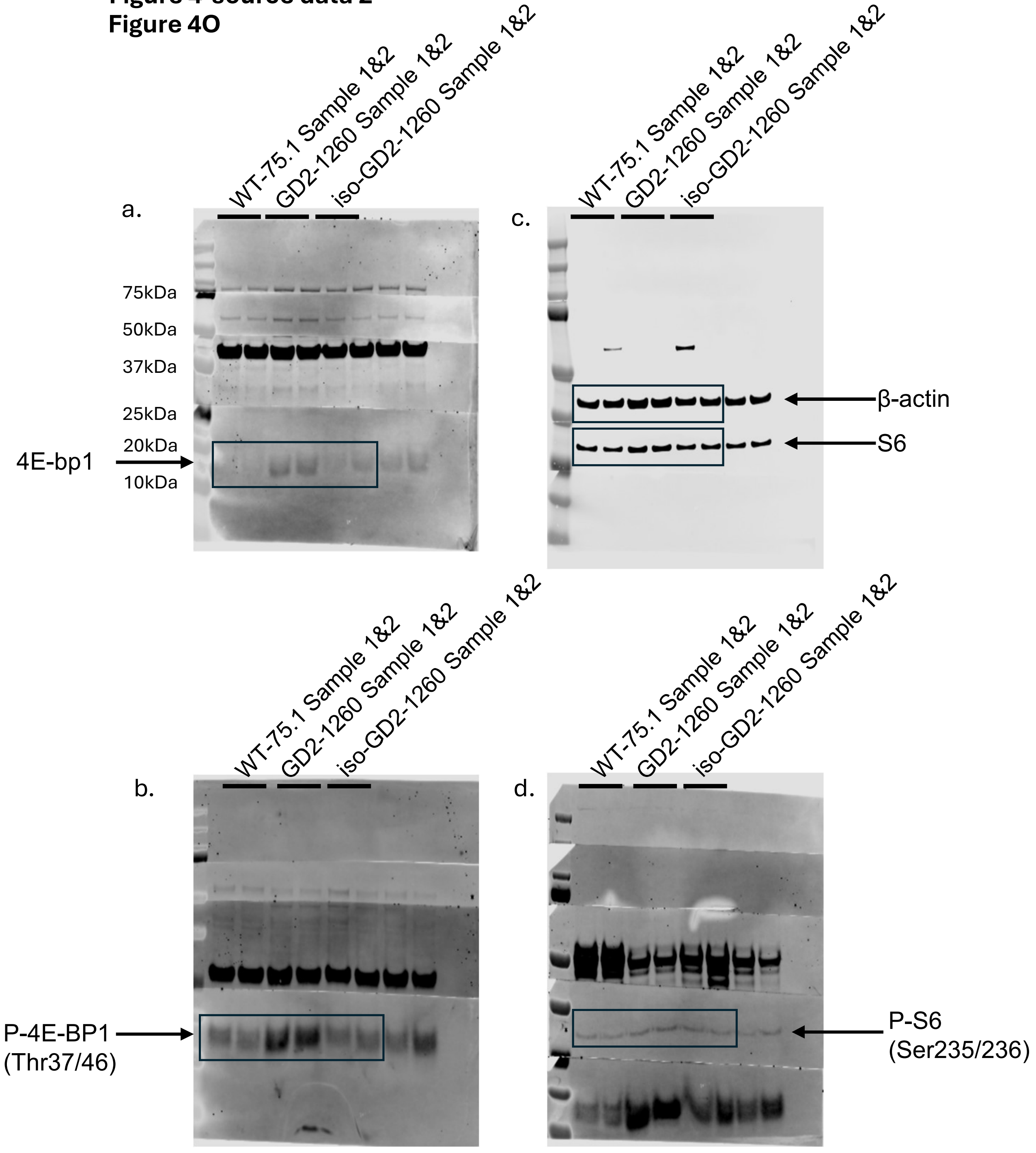

Figure 4-Source Data 2_4O. Original membranes corresponding to Figure 4, panel O. Original blots for 4E-bp1 (panel a), P-4E-BP1 (Thr37/46) (panel b), , S6 (panel c), , P-S6 (Ser235/236) (panel d), and loading control β-actin (panel c). The same sets of samples, each with the same amount of total protein, was loaded on separate gels to detect the indicated targets. Precision Plus Protein Dual Color Standards were used. Other lanes, along with other bands on membranes corresponding to different tested targets, are not shown in Figure 4O.
