# Supplementary material for: Patient-specific midbrain organoids with CRISPR correction recapitulate neuronopathic Gaucher disease phenotypes and enable evaluation of novel therapies
Source: eLife. 2026 Jun 23;15:RP109518. doi: 10.7554/eLife.109518 (PMC13290227; doi:10.7554/eLife.109518)
Supplement: Figure 4—figure supplement 1—source data 2. [file elife-109518-fig4-figsupp1-data2.zip › Figure 4-figure supplement 1-source data 2.pptx]

## Slide 1
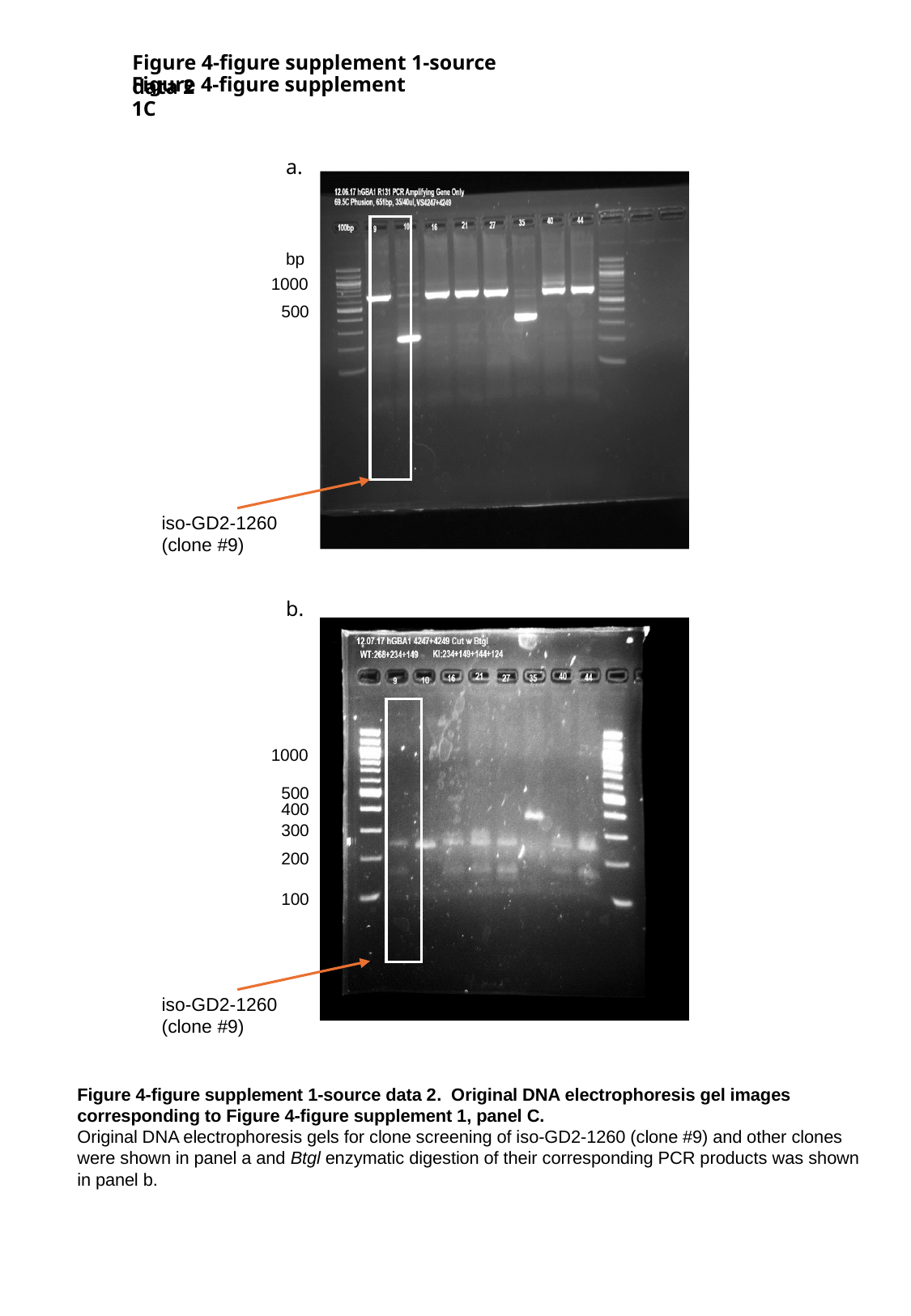

Figure 4-figure supplement 1-source data 2
Figure 4-figure supplement 1C
a.
bp
1000
500
iso-GD2-1260 (clone #9)
b.
1000
500
400
300
200
100
iso-GD2-1260 (clone #9)
Figure 4-figure supplement 1-source data 2. Original DNA electrophoresis gel images corresponding to Figure 4-figure supplement 1, panel C.
Original DNA electrophoresis gels for clone screening of iso-GD2-1260 (clone #9) and other clones were shown in panel a and Btgl enzymatic digestion of their corresponding PCR products was shown in panel b.
