# Supplementary material for: Patient-specific midbrain organoids with CRISPR correction recapitulate neuronopathic Gaucher disease phenotypes and enable evaluation of novel therapies
Source: eLife. 2026 Jun 23;15:RP109518. doi: 10.7554/eLife.109518 (PMC13290227; doi:10.7554/eLife.109518)
Supplement: Figure 5—source data 2. [file elife-109518-fig5-data2.zip › Figure 5-source data 2.pdf]

Figure 5-source data 2
Figure 5E

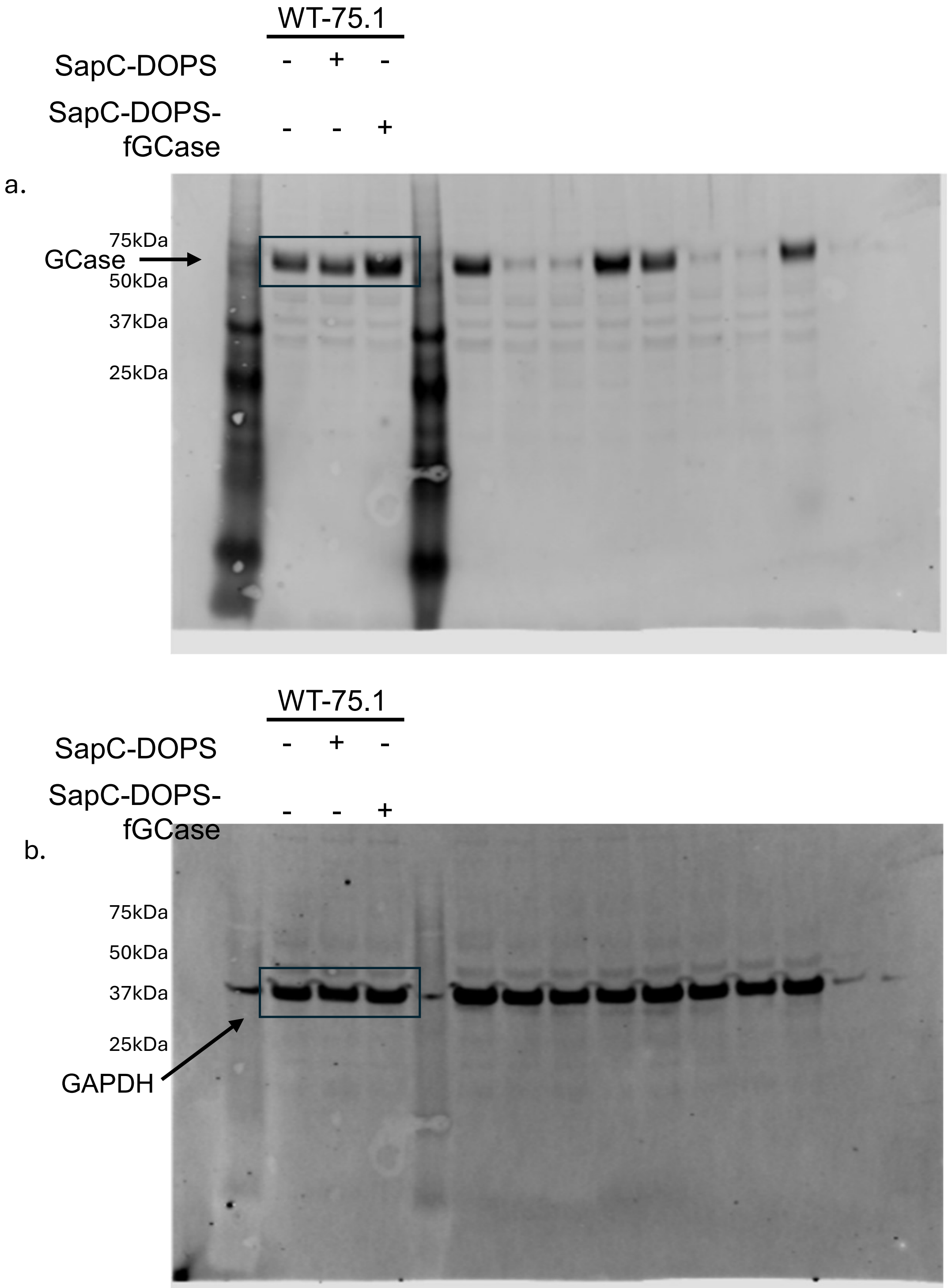

Figure 5-Source Data 2_5E. Original membranes corresponding to Figure 5, panel E. Original blots for Gcase (panel a) and loading control GAPDH (panel b). Precision Plus Protein Dual Color Standards were used. Other lanes are not shown in Figure 5E.

Figure 5-source data 2
Figure 5F

a.

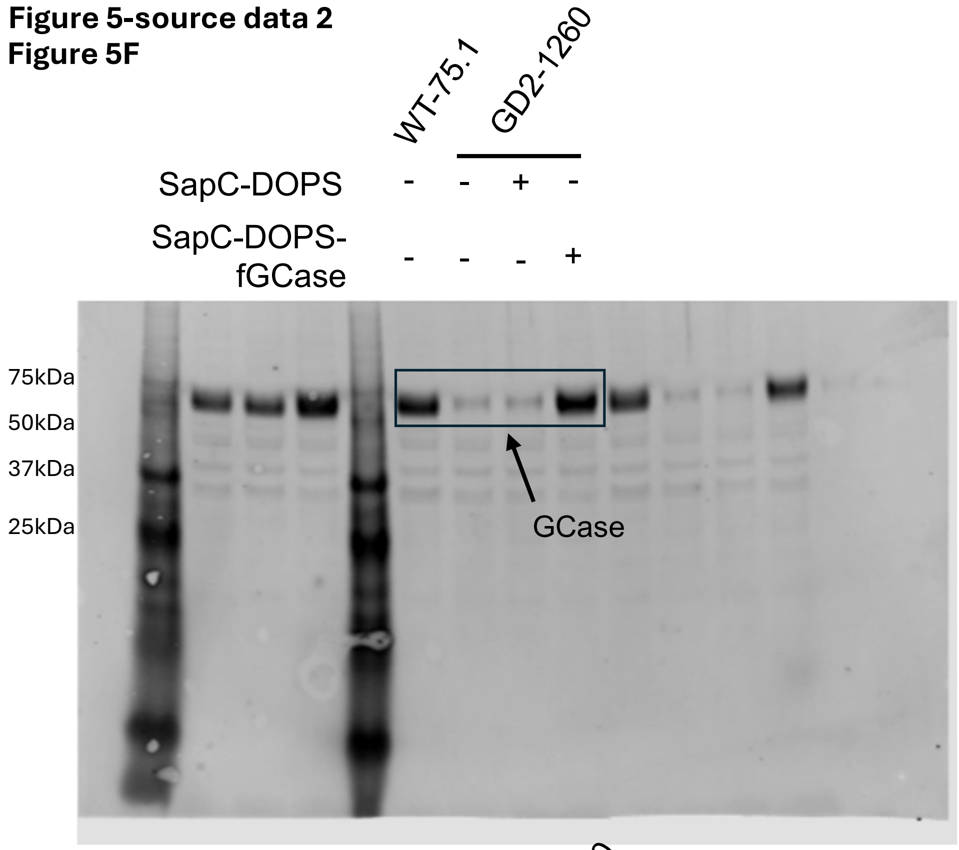

b.

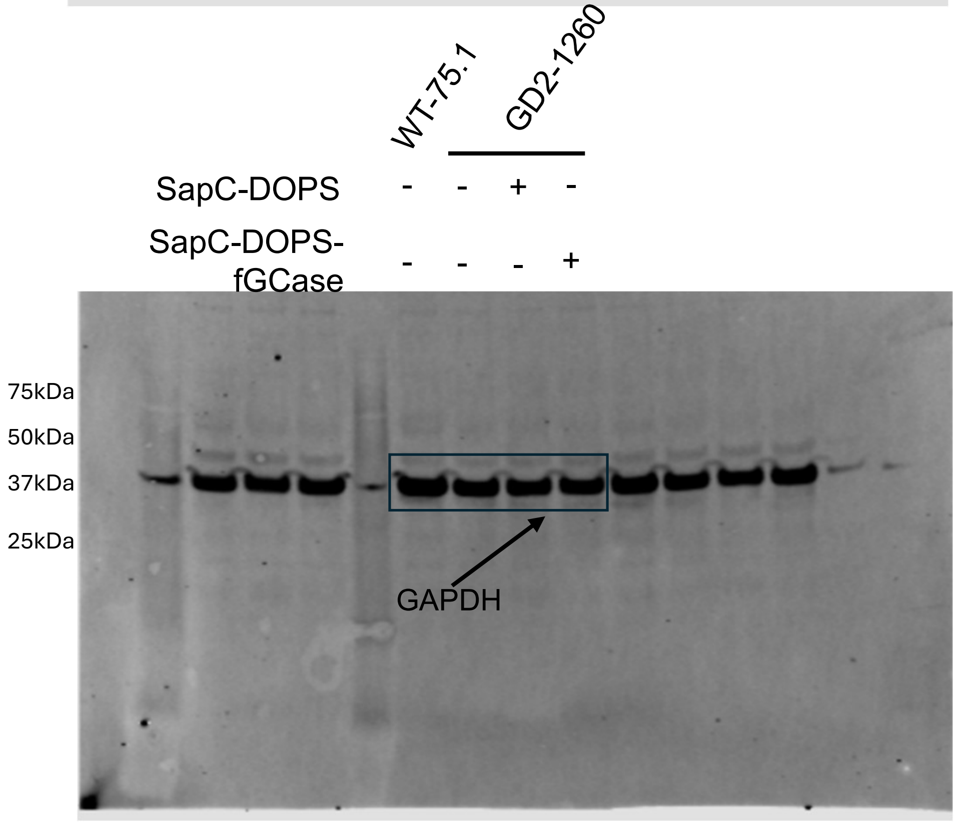

Figure 5-Source Data 2_5F. Original membranes corresponding to Figure 5, panel F. Original blots for GCase and loading control GAPDH. Precision Plus Protein Dual Color Standards were used. Other lanes are not shown in Figure 5F.

Figure 5-source data 2
Figure 5G

a.

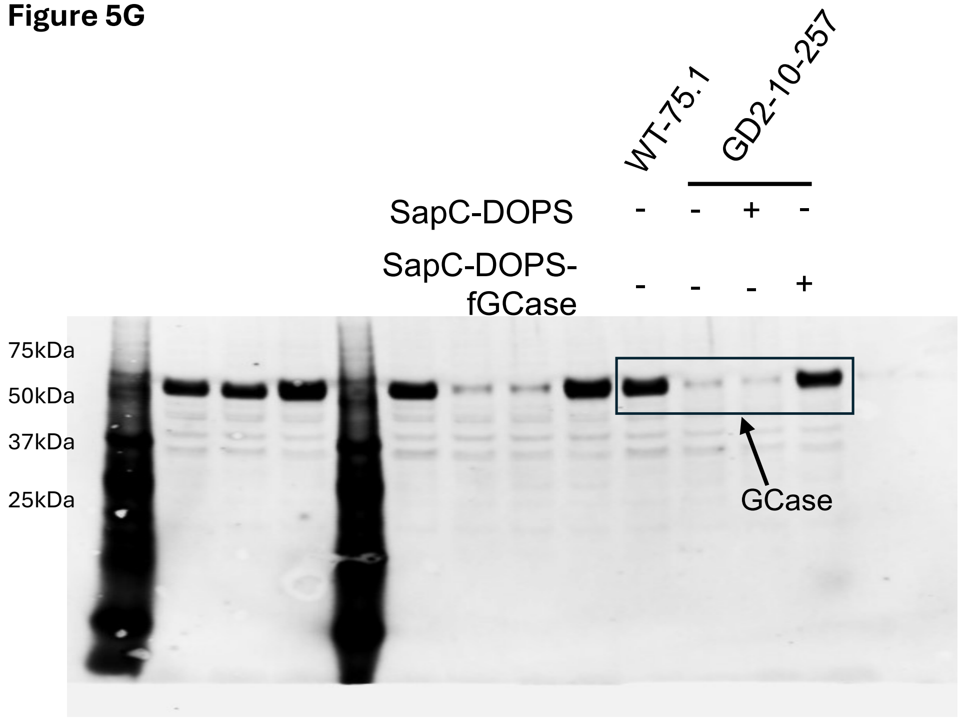

b.

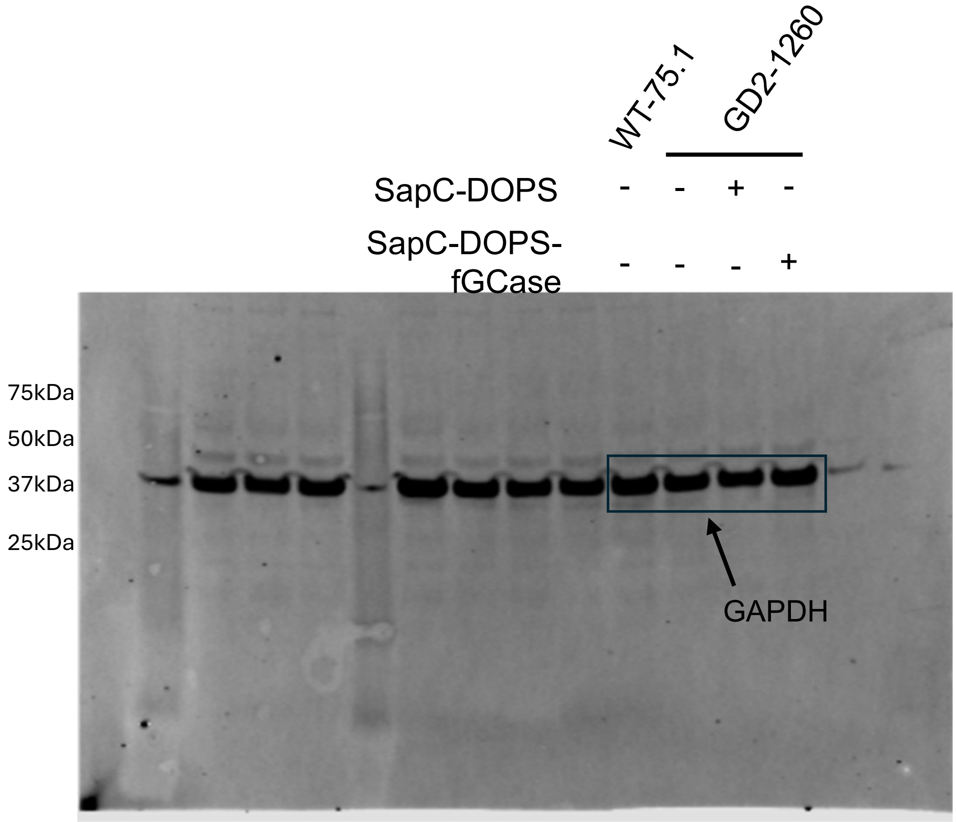

Figure 5-Source Data 2_5G. Original membranes corresponding to Figure 5, panel G. Original blots for GCase and loading control GAPDH. Precision Plus Protein Dual Color Standards were used. Other lanes are not shown in Figure 5G.

Figure 5-source data 2
Figure 5J

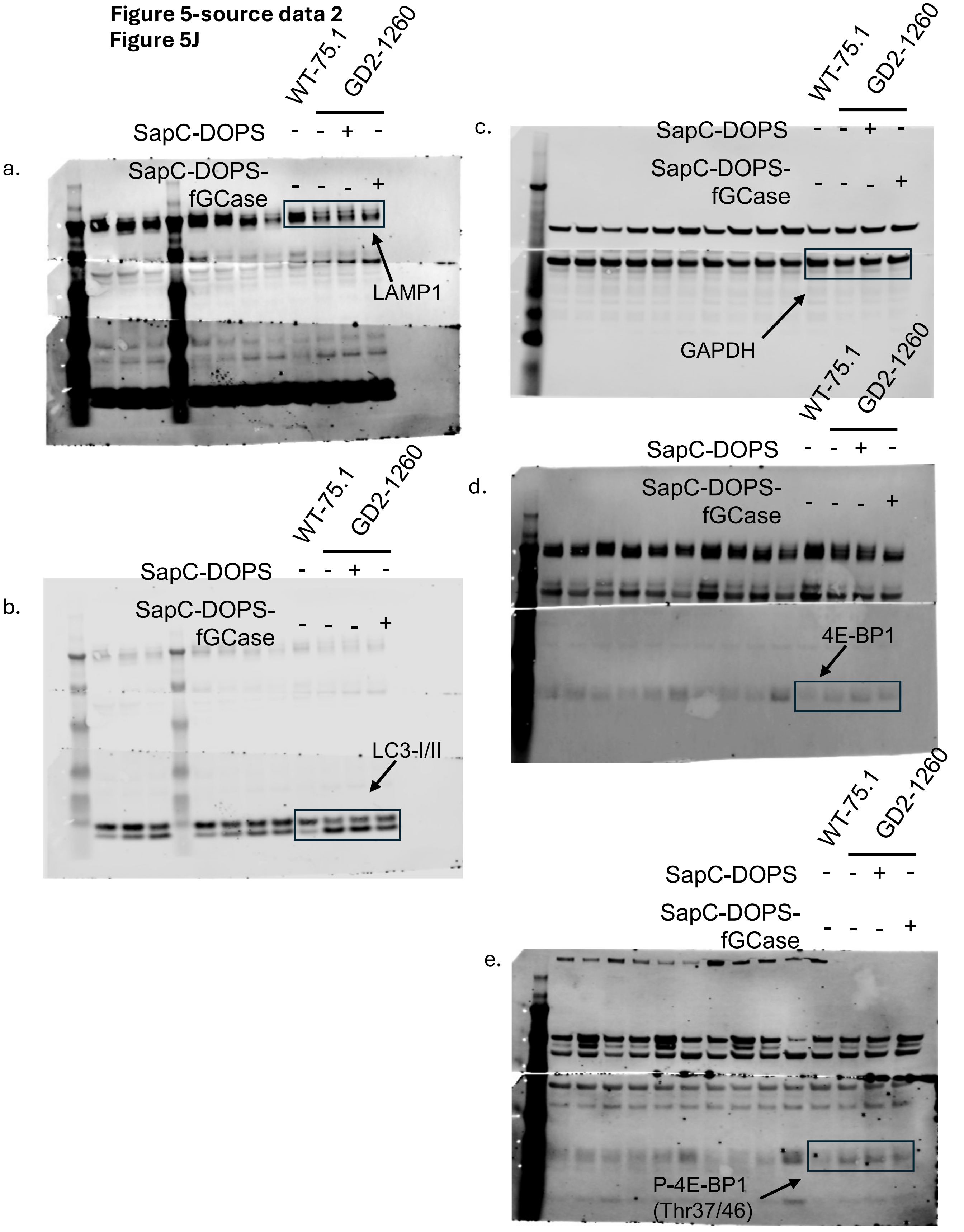

Figure 5-Source Data 2_5J. Original membranes corresponding to Figure 5, panel J. Original blots for LAMP1 (panel a), LC3-I/II (panel b), 4E-bp1 (panel d), P-4E-BP1 (Thr37/46) (panel e), and loading control GAPDH (panel c). Precision Plus Protein Dual Color Standards were used. Other lanes and targets blotted in these membranes are not shown in Figure 5J.
