# Supplementary material for: Patient-specific midbrain organoids with CRISPR correction recapitulate neuronopathic Gaucher disease phenotypes and enable evaluation of novel therapies
Source: eLife. 2026 Jun 23;15:RP109518. doi: 10.7554/eLife.109518 (PMC13290227; doi:10.7554/eLife.109518)
Supplement: Figure 5—figure supplement 1—source data 2. [file elife-109518-fig5-figsupp1-data2.zip › Figure 5-figure supplement 1-source data 2.pdf]

Figure 5-figure supplement 1-source data 2
Figure 5-figure supplement 1E

a.

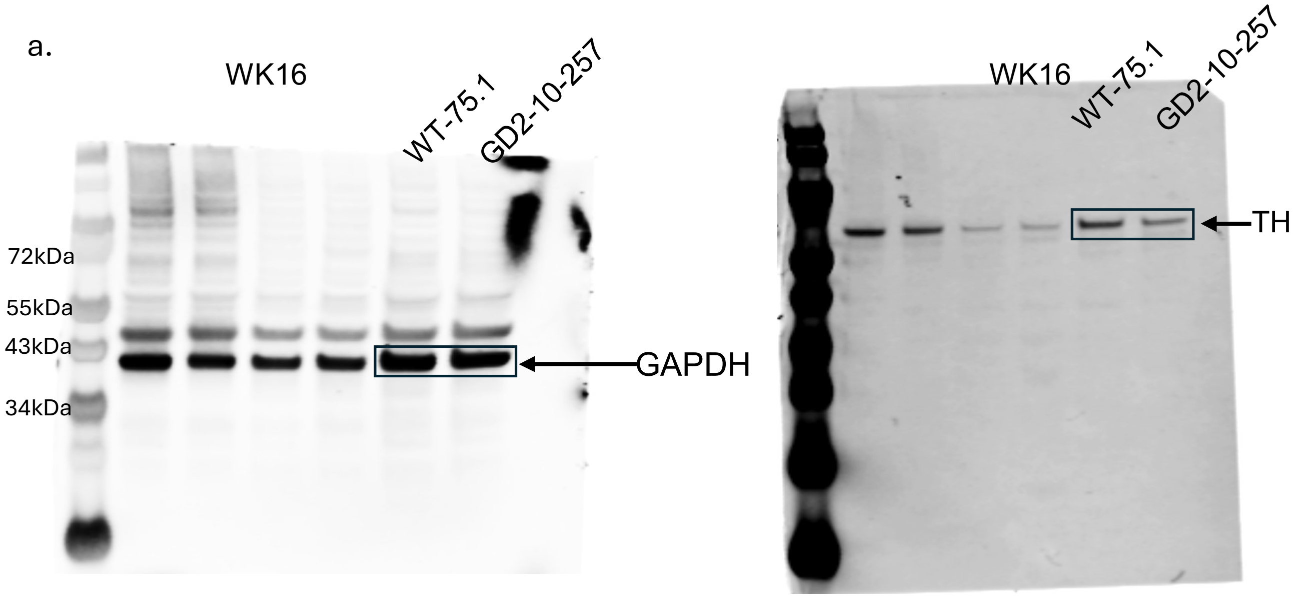

b.

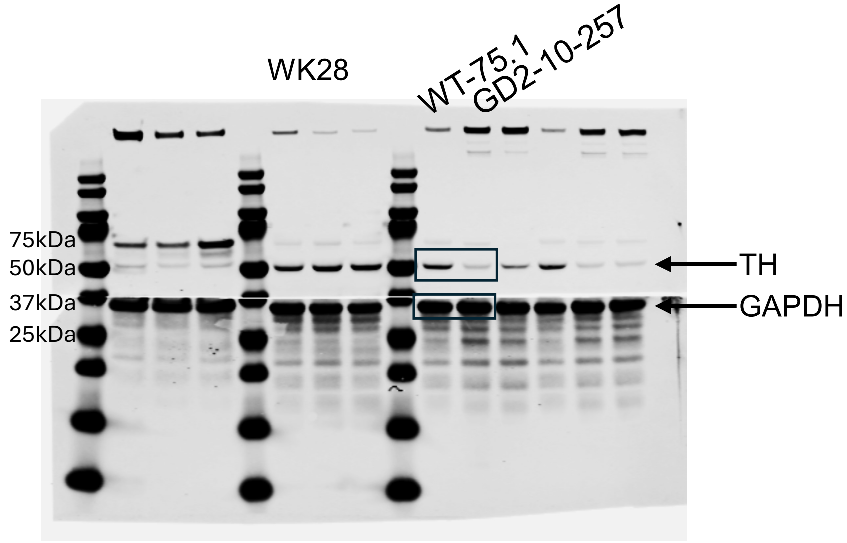

Figure 5-figure supplement 1-source data 2. Original membranes corresponding to Figure 5-figure supplement 1, panel E.
Original blots for TH and loading control GAPDH at WK 16 (panel a) and WK28 (panel b). Precision Plus Protein Dual Color Standards were used. Other lanes are not shown in Figure 5-figure supplement 1, panel E.
