# Supplementary material for: Patient-specific midbrain organoids with CRISPR correction recapitulate neuronopathic Gaucher disease phenotypes and enable evaluation of novel therapies
Source: eLife. 2026 Jun 23;15:RP109518. doi: 10.7554/eLife.109518 (PMC13290227; doi:10.7554/eLife.109518)
Supplement: Figure 6—source data 2. [file elife-109518-fig6-data2.zip › Figure 6-source data 2.pdf]

Figure 6-source data 2

Figure 6B

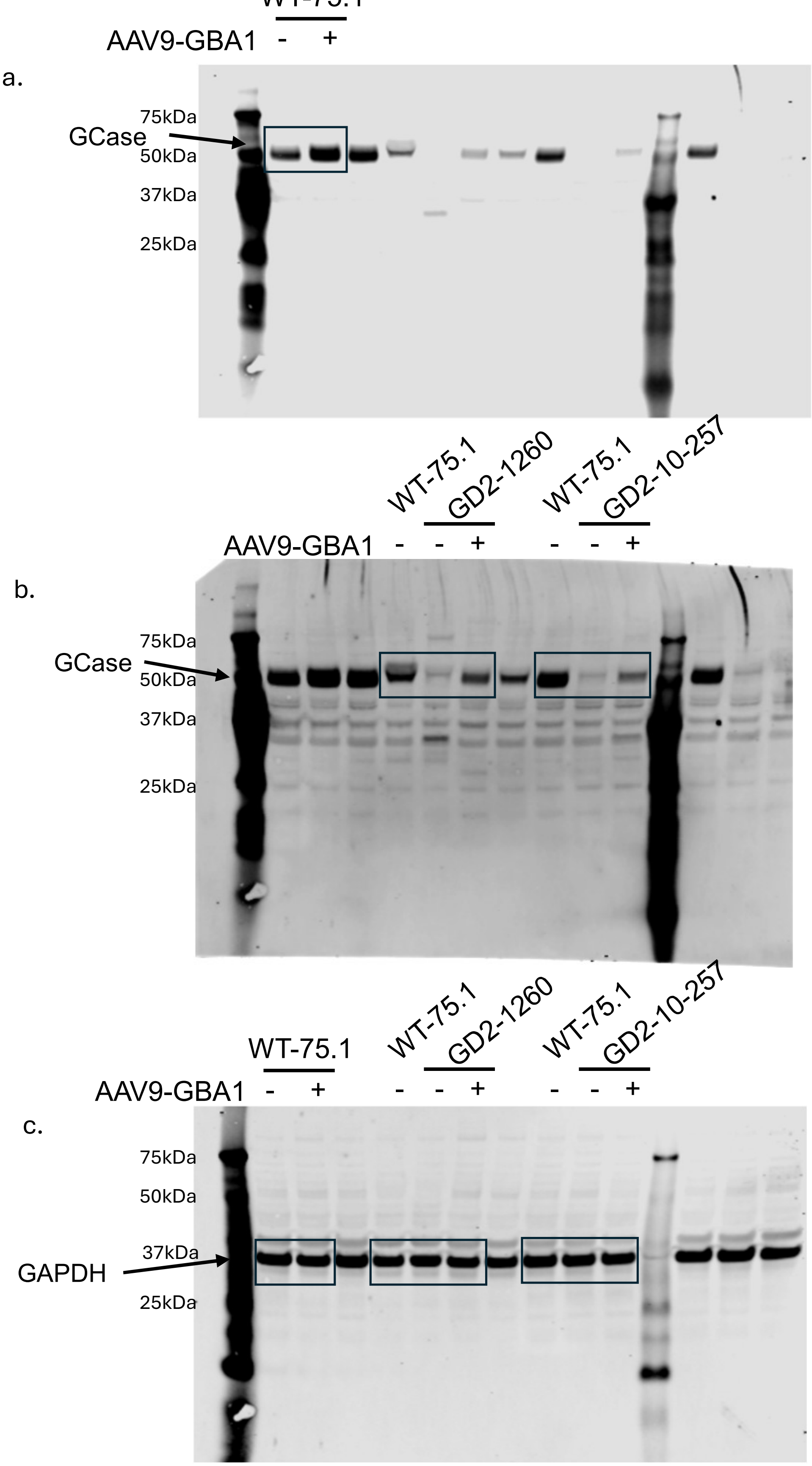

Figure 6-Source Data 2_6B. Original membranes corresponding to Figure 6, panel B. Original blots for GCase (panels a and b) and loading control GAPDH (panel c). Precision Plus Protein Dual Color Standards were used. Other lanes are not shown in Figure 6B.

Figure 6-source data 2
Figure 6D

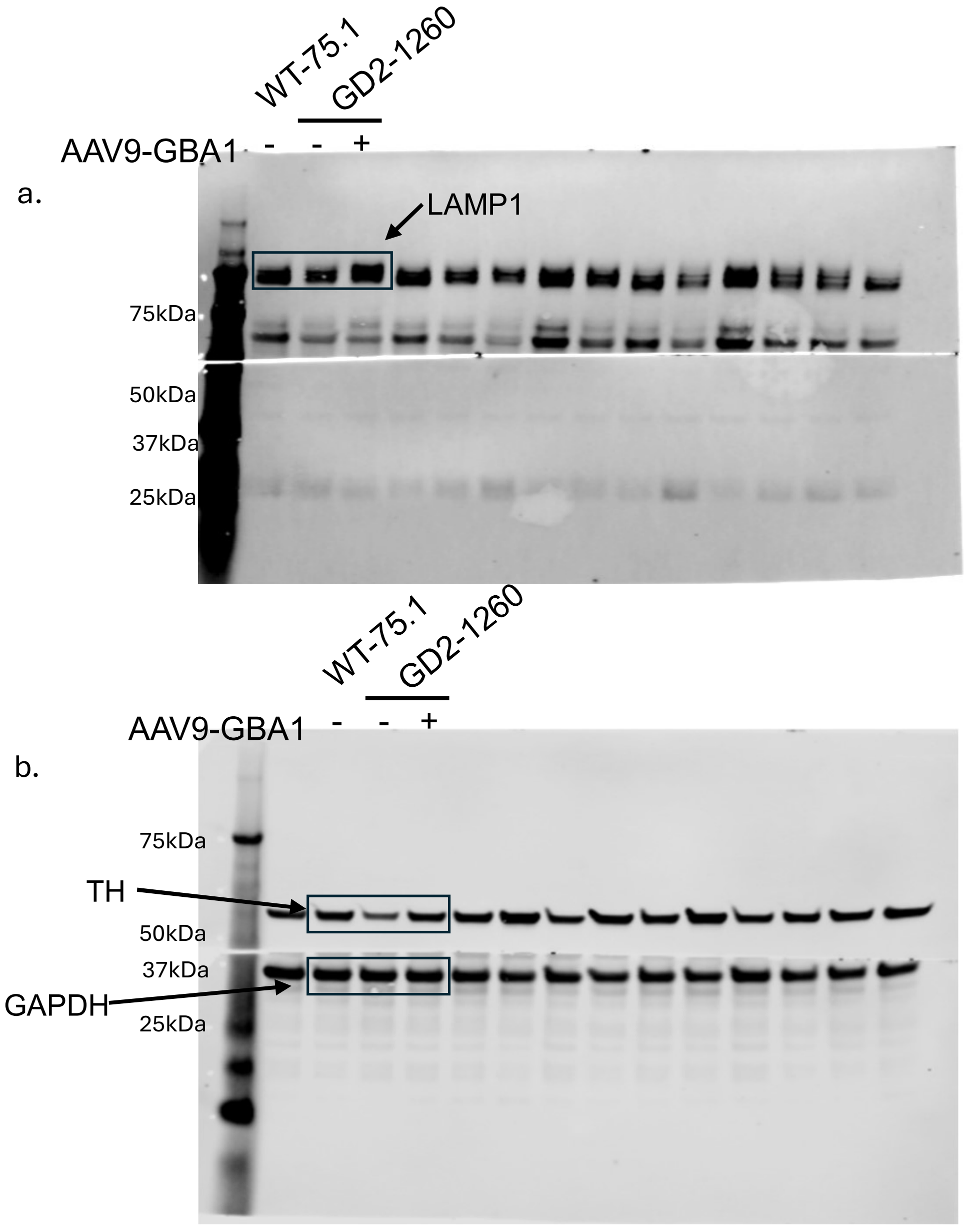

Figure 6-Source Data 2_6D. Original membranes corresponding to Figure 6, panel D. Original blots for LAMP1 (panel a), TH and loading control GAPDH (panel b). Precision Plus Protein Dual Color Standards were used. Other lanes are not shown in Figure 6D.
