# Supplementary material for: Patient-specific midbrain organoids with CRISPR correction recapitulate neuronopathic Gaucher disease phenotypes and enable evaluation of novel therapies
Source: eLife. 2026 Jun 23;15:RP109518. doi: 10.7554/eLife.109518 (PMC13290227; doi:10.7554/eLife.109518)
Supplement: Figure 7—source data 2. [file elife-109518-fig7-data2.zip › Figure 7-source data 2.pdf]

Figure 7-source data 1
Figure 7I

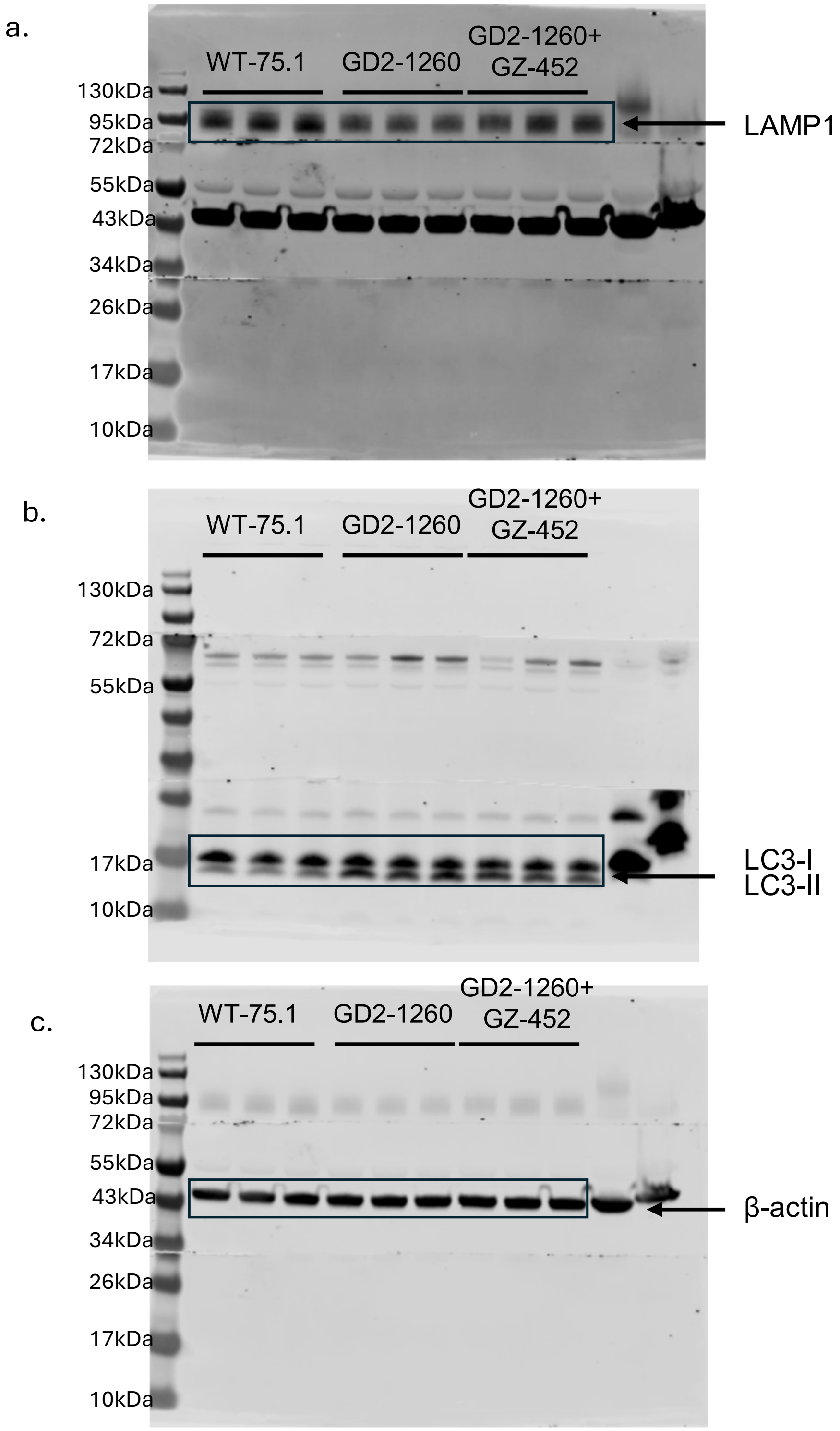

Figure 7-Source Data 2. Original membranes corresponding to Figure 7, panel I.
Original blots for Lamp1 (panel a), LC3-I/II (panel b) and loading control β-actin (panel c). EZ-Run™ Prestained Rec Protein Ladder were used. Other lanes are not shown in Figure 7I.
