# Supplementary material for: Patient-specific midbrain organoids with CRISPR correction recapitulate neuronopathic Gaucher disease phenotypes and enable evaluation of novel therapies
Source: eLife. 2026 Jun 23;15:RP109518. doi: 10.7554/eLife.109518 (PMC13290227; doi:10.7554/eLife.109518)
Supplement: Supplementary file 1. [file elife-109518-supp1.docx]

**Supplementary File 1. Summary of therapeutic modalities on nGD MLOs.**

|  |  | **GCase activity**  **(% of WT, Mean ± SEM)** | | | **Substrate GluSph level**  **(Fold to WT, Mean ± SEM)** | | | **TH expression**  **(Fold to WT, Mean ± SEM)** | | |
| --- | --- | --- | --- | --- | --- | --- | --- | --- | --- | --- |
| **iPSC line derived MLO** | **Therapeutic modalities** | **w/o treatment** | **w/ treatment** | **P value** | **w/o treatment** | **w/ treatment** | **P value** | **w/o treatment** | **w/ treatment** | **P value** |
| **GD2-1260** | CRISPR/Cas9-mediated gene correction | 15.9% ± 2.0%  Wk8 | 46.3% ± 1.8% Wk8 | P<0.001 | 6.6 ± 1.6  Wk28 | 1.4 ± 0.3  Wk28 | P<0.05 | 0.24 ± 0.02  Wk8 | 1.06 ± 0.19  Wk8 | P<0.001 |
|  | SapC-DOPS-fGCase  (2 weeks treatment) | 5.6% ± 0.5% ~Wk15 | 95.2% ± 2.1% ~Wk15 | P<0.001 | 3.9 ± 0.8  Wk16 | 0.7 ± 0.1  Wk16 | P<0.001 | N/A | N/A | N/A |
|  | AAV9-GBA1  (3 weeks treatment) | 6.0% ± 0.6%  Wk16 | 47.8% ± 2.3%  Wk16 | P<0.001 | 3.1± 0.4  Wk16 | 0.3 ± 0.03  Wk16 | P<0.001 | 0.65 | 0.73 | n.s. |
|  | GZ452 | N/A | N/A | N/A | 5.9 ± 0.8  Wk15 | 1.1 ± 0.03  Wk15 | P<0.001 | N/A | N/A | N/A |
| **GD2-10-257** | CRISPR/Cas9-mediated gene correction | 8.8% ± 0.2% ~Wk15 | N/A | N/A | 3.6 ± 0.02  Wk16 | N/A | N/A | 0.20/Wk16  0.17/Wk28 | N/A | N/A |
|  | SapC-DOPS-fGCase  (2 weeks treatment) | 8.8% ± 0.2% ~Wk15 | 88.0% ± 9.1% ~Wk15 | P<0.001 | 7.6± 0.9  Wk16 | 3.3 ± 1.0  Wk16 | P<0.001 | N/A | N/A | N/A |
|  | AAV9-GBA1  (3 weeks treatment) | 8.8% ± 0.2%  Wk16 | 37.7% ± 4.0%  Wk16 | P<0.001 | 8.4 ± 0.5  Wk16 | 0.6 ± 0.1  Wk16 | P<0.001 | N/A | N/A | N/A |
|  | GZ452 | N/A | N/A | N/A | N/A | N/A | N/A | N/A | N/A | N/A |

N/A, not available; n.s., not significant. GluSph, glucosylsphingosine.
