# Supplementary material for: Patient-specific midbrain organoids with CRISPR correction recapitulate neuronopathic Gaucher disease phenotypes and enable evaluation of novel therapies
Source: eLife. 2026 Jun 23;15:RP109518. doi: 10.7554/eLife.109518 (PMC13290227; doi:10.7554/eLife.109518)
Supplement: Supplementary file 2. [file elife-109518-supp2.docx]

**Supplementary File 2. Dysregulated pathways in nGD models.**

| **Model** | **Tissue** | **Key Dysregulated Pathways** |
| --- | --- | --- |
| Human nGD organoid | Midbrain-like organoids | Nervous system development  Axon guidance  Neuron differentiation  Dopaminergic/Glutamatergic/GABAergic synapse  Apoptosis etc. |
| Mouse nGD model^#^ | Brain (Midbrain region) | Neurological disease  Axonal guidance signaling  Dopamine/Glutamate/GABA receptor signaling  Lipid metabolism  Cell death and survival etc. |

#, Data (GEO accession number: GSE67375) from “Dasgupta, N., Xu, Y.H., Li, R., Peng, Y., Pandey, M.K., Tinch, S.L., Liou, B., Inskeep, V., Zhang, W., Setchell, K.D., Keddache, M., et al. (2015). Neuronopathic Gaucher disease: dysregulated mRNAs and miRNAs in brain pathogenesis and effects of pharmacologic chaperone treatment in a mouse model. Human molecular genetics 24, 7031–7048. 10.1093/hmg/ddv404.”
