# Supplementary material for: Patient-specific midbrain organoids with CRISPR correction recapitulate neuronopathic Gaucher disease phenotypes and enable evaluation of novel therapies
Source: eLife. 2026 Jun 23;15:RP109518. doi: 10.7554/eLife.109518 (PMC13290227; doi:10.7554/eLife.109518)
Supplement: Peer review history

# Editor report: eLife Assessment

**DOI:** 10.7554/eLife.109518.3.sa0

**Authors:** Hugo J Bellen

**Affiliations:** Baylor College of Medicine United States

**Keywords:** Convincing, Solid, Important

This study provides potentially **important** insights by establishing a human disease model and exploring therapeutic approaches. The evidence is generally **convincing** for descriptive and comparative findings. The authors present **solid** data, but evidence for proposed biological mechanisms and functional outcomes remains limited.

# Referee report: Reviewer #1 (Public review)

**DOI:** 10.7554/eLife.109518.3.sa1

In this study, the authors set out to develop a human disease model using stem cell-derived systems and to use this platform to investigate disease biology and evaluate potential therapeutic approaches. Their goal is to provide a tractable experimental system that captures key features of the disease and enables testing of candidate interventions.

The work has several important strengths. The authors present a carefully constructed model with improved genetic replication and clearer reporting of biological replicates, which enhances confidence in the reproducibility of the findings. The longitudinal design, spanning early developmental stages to later disease-relevant phenotypes, provides a useful framework for distinguishing temporal aspects of the disease process. The study also includes a comparative evaluation of multiple therapeutic strategies adding practical value to the field. In addition, statistical reporting and transparency have been strengthened, and key limitations of the model-such as the absence of certain cell types-are now clearly acknowledged.

At the same time, notable weaknesses temper the strength of the conclusions. Several central biological claims, particularly those related to specific signaling pathways, are supported primarily by transcriptomic and protein-level observations without direct functional validation. Similarly, measures used to interpret cellular processes do not fully distinguish between alternative biological explanations, leaving some mechanistic interpretations unresolved. The therapeutic findings are supported by biochemical changes, but evidence for functional recovery at the cellular level is limited. These gaps mean that some of the broader conclusions should be interpreted with caution.

Overall, the authors have largely achieved their aim of establishing a useful experimental model and demonstrating its potential for studying disease-related changes and testing interventions. The evidence is convincing for the descriptive and comparative aspects of the work, but more limited for mechanistic and functional claims.

The study is likely to have a meaningful impact by providing a platform that others in the field can build upon. The methods and datasets will be useful to researchers interested in disease modeling and therapeutic development. At the same time, the work is best viewed as an important foundation, with key mechanistic and functional questions remaining to be addressed in future studies.

# Referee report: Reviewer #2 (Public review)

**DOI:** 10.7554/eLife.109518.3.sa2

Sun et al. have developed a midbrain-like organoid (MLO) model for neuronopathic Gaucher disease (nGD). The MLOs recapitulate several features of nGD molecular pathology, including reduced GCase activity, sphingolipid accumulation, and impaired dopaminergic neuron development. They also characterize the transcriptome in the MLO nGD model. CRISPR correction of one of the GBA1 mutant alleles rescues most of the nGD molecular phenotypes. The MLO model was further deployed in proof-of-principle studies of investigational nGD therapies, including SapC-DOPS nanovesicles, AAV9-mediated GBA1 gene delivery, and substrate-reduction therapy (GZ452). This patient-specific 3D model provides a new platform for studying nGD mechanisms and accelerating therapy development. Overall, only modest weaknesses are noted, and these have been adequately addressed in the revision.

Comments on revisions:

I have no further recommendations. The revised manuscript addresses the few questions and concerns that I had initially shared.

# Referee report: Reviewer #3 (Public review)

**DOI:** 10.7554/eLife.109518.3.sa3

Summary:

In this study, the authors describe modeling of neuronopathic Gaucher disease (nGD) using midbrain-like organoids (MLOs) derived from hiPSCs carrying GBA1 L444P/P415R or L444P/RecNciI variants. These MLOs recapitulate several disease features, including GCase deficiency, reduced enzymatic activity, lipid substrate accumulation, and impaired dopaminergic neuron differentiation. Correction of the GBA1 L444P variant restored GCase activity, normalized lipid metabolism, and rescued dopaminergic neuronal defects, confirming its pathogenic role in the MLO model. The authors further leveraged this system to evaluate therapeutic strategies, including: (i) SapC-DOPS nanovesicles for GCase delivery, (ii) AAV9-mediated GBA1 gene therapy, and (iii) GZ452, a glucosylceramide synthase inhibitor. These treatments reduced lipid accumulation and ameliorated autophagic, lysosomal, and neurodevelopmental abnormalities.

Strengths:

This manuscript demonstrates that nGD patient-derived MLOs can serve as an additional platform for investigating nGD mechanisms and advancing therapeutic development.

Comments on revisions:

I have no further concerns regarding this manuscript.

# Author comment: Author response

**DOI:** 10.7554/eLife.109518.3.sa4

**Authors:** Yi Lin, Benjamin Liou, Venette Fannin, Stuart Adler, Christopher N Mayhew, Jason E Hammonds, Yueh-Chiang Hu, Jason Tchieu, Wujuan Zhang, Xueheng Zhao, Rebecca L Beres, Kenneth DR Setchell, Ahmet Kaynak, Xiaoyang Qi, Ricardo A Feldman, Ying Sun

**Affiliations:** Cincinnati Children's Hospital Medical Center Cincinnati United States; Cincinnati Children's Hospital Medical Center Cincinnati United States; Cincinnati Children's Hospital Medical Center Cincinnati United States; Cincinnati Children's Hospital Medical Center Cincinnati United States; Cincinnati Children's Hospital Medical Center Cincinnati United States; Cincinnati Children's Hospital Medical Center Cincinnati United States; Cincinnati Children's Hospital Medical Center Cincinnati United States; Cincinnati Children's Hospital Medical Center Cincinnati United States; Cincinnati Children's Hospital Medical Center Cincinnati United States; Cincinnati Children's Hospital Medical Center Cincinnati United States; Cincinnati Children's Hospital Medical Center Cincinnati United States; Cincinnati Children's Hospital Medical Center Cincinnati United States; University of Cincinnati Cincinnati United States; University of Cincinnati Cincinnati United States; University of Maryland, School of Medicine Baltimore United States; Cincinnati Children's Hospital Medical Center Cincinnati United States

The following is the authors’ response to the original reviews

**Public Reviews:**

**Reviewer #1 (Public review):**

Summary:

This manuscript by Lin et al. presents a timely, technically strong study that builds patient-specific midbrain-like organoids (MLOs) from hiPSCs carrying clinically relevant GBA1 mutations (L444P/P415R and L444P/RecNcil). The authors comprehensively characterize nGD phenotypes (GCase deficiency, GluCer/GluSph accumulation, altered transcriptome, impaired dopaminergic differentiation), perform CRISPR correction to produce an isogenic line, and test three therapeutic modalities (SapC-DOPS-fGCase nanoparticles, AAV9GBA1, and SRT with GZ452). The model and multi-arm therapeutic evaluation are important advances with clear translational value.

My overall recommendation is that the work undergo a major revision to address the experimental and interpretive gaps listed below.

Strengths:

(1) Human, patient-specific midbrain model: Use of clinically relevant compound heterozygous GBA1 alleles (L444P/P415R and L444P/RecNcil) makes the model highly relevant to human nGD and captures patient genetic context that mouse models often miss.

(2) Robust multi-level phenotyping: Biochemical (GCase activity), lipidomic (GluCer/GluSph by UHPLC-MS/MS), molecular (bulk RNA-seq), and histological (TH/FOXA2, LAMP1, LC3) characterization are thorough and complementary.

(3) Use of isogenic CRISPR correction: Generating an isogenic line (WT/P415R) and demonstrating partial rescue strengthens causal inference that the GBA1 mutation drives many observed phenotypes.

(4) Parallel therapeutic testing in the same human platform: Comparing enzyme delivery (SapC-DOPS-fGCase), gene therapy (AAV9-GBA1), and substrate reduction (GZ452) within the same MLO system is an elegant demonstration of the platform's utility for preclinical evaluation.

(5) Good methodological transparency: Detailed protocols for MLO generation, editing, lipidomics, and assays allow reproducibility

Weaknesses:

(1) Limited genetic and biological replication

(a) Single primary disease line for core mechanistic claims. Most mechanistic data derive from GD2-1260 (L444P/P415R); GD2-10-257 (L444P/RecNcil) appears mainly in therapeutic experiments. Relying primarily on one patient line risks conflating patient-specific variation with general nGD mechanisms.

We thank the reviewer for highlighting the importance of genetic and biological replication. An additional patient-derived iPSC line was included in the manuscript, therefore, our study includes two independent nGD patient-derived iPSC lines, GD2-1260 (GBA1^L444P/P415R^) and GD2-10-257 (GBA1^L444P/RecNcil^), both of which carry the severe mutations associated with nGD. These two lines represent distinct genetic backgrounds and were used to demonstrate the consistency of key disease phenotypes (reduced GCase activity, elevated substrate, impaired dopaminergic neuron differentiation etc.) across different patient’s MLOs. Major experiments (e.g., GCase activity assays, substrate, immunoblotting for DA marker TH, and therapeutic testing with SapC-DOPS-fGCase, AAV9-GBA1) were performed using both patient lines, with results showing consistent phenotypes and therapeutic responses (see Figs. 2-6, and Supplementary Figs. 4-5). To ensure clarity and transparency, a new Supplementary Table 2 summarizes the characterization of both, the GD2-1260 and GD2-10-257 lines.

(b) Unclear biological replicate strategy. It is not always explicit how many independent differentiations and organoid batches were used (biological replicates vs. technical fields of view).

Biological replication was ensured in our study by conducting experiments in at least 3 independent differentiations per line, and technical replicates (multiple organoids/fields per batch) were averaged accordingly. We have clarified biological replicates and differentiation in the figure legends.

(c) A significant disadvantage of employing brain organoids is the heterogeneity during induction and potential low reproducibility. In this study, it is unclear how many independent differentiation batches were evaluated and, for each test (for example, immunofluorescent stain and bulk RNA-seq), how many organoids from each group were used. Please add a statement accordingly and show replicates to verify consistency in the supplementary data.

In the revision, we have clarified biological replicates and differentiation in the figure legend in Fig.1E; Fig.2B,2G; Fig.3F, 3G; Fig.4B-C,E,H-J, M-N; Fig.6D; and Fig.7A-C, I.

(d) Isogenic correction is partial. The corrected line is WT/P415R (single-allele correction); residual P415R complicates the interpretation of "full" rescue and leaves open whether the remaining pathology is due to incomplete correction or clonal/epigenetic effects.

We attempted to generate an isogenic iPSC line by correcting both GBA1 mutations (L444P and P415R). However, this was not feasible because GBA1 overlaps with a highly homologous pseudogene (PGBA), which makes precise editing technically challenging. Consequently, only the L444P mutation was successfully corrected, and the resulting isogenic line retains the P415R mutation in a heterozygous state. Because Gaucher disease is an autosomal recessive disorder, individuals carrying a single GBA1 mutation (heterozygous carriers) do not develop clinical symptoms. Therefore, the partially corrected isogenic line, which retains only the P415R allele, represents a clinically relevant carrier model. Consistent with this, our results show that GCase activity was restored to approximately 50% of wild-type levels (Fig.4B-C), supporting the expected heterozygous state. These findings also make it unlikely that the remaining differences observed are due to clonal variation or epigenetic effects.

(e) The authors tested week 3, 4, 8, 15, and 28 old organoids in different settings. However, systematic markers of maturation should be analyzed, and different maturation stages should be compared, for example, comparing week 8 organoids to week 28 organoids, with immunofluorescent marker staining and bulk RNAseq.

We agree that a systematic analysis of maturation stages is essential for validating the MLO model. Our data integrated a longitudinal comparison across multiple developmental windows (Weeks 3 to 28) to characterize the transition from progenitors to mature/functional states for nGD phenotyping and evaluation of therapeutic modalities: (1) DA differentiation (Wks 3 and 8 in Fig. 3): qPCR analysis demonstrated the progression of DA-specific programs. We observed a steady increase in the mature DA neuron marker *TH* and *ASCL1*. This was accompanied by a gradual decrease in early floor plate/progenitor markers *FOXA2* and *PLZF*, indicating a successful differentiation path from progenitors to differentiated/mature DA neurons. (2) Glycosphingolipid substrates accumulation (Wks 15 and 28 in Fig 2): To assess late-stage nGD phenotyping, we compared GluCer and GluSph at Week 15 and Week 28. This comparison highlights the progressive accumulation of substrates in nGD MLOs, reflecting the metabolic consequences of the disease at different mature stage. (3) Organoid growth dynamics (Wks 4, 8, and 15 in new Fig. 4): The new Fig. 4 tracks physical maturation through organoid size and growth rates across three key time points, providing a macro-scale verification of consistent development between WT and nGD groups. By comparing these early (Wk 3-8) and late (Wk 15-28) stages, we confirmed that our MLOs transition from a proliferative state to a post-mitotic, specialized neuronal state, satisfied the requirement for comparing distinct maturation stages.

(f) The manuscript frequently refers to Wnt signaling dysregulation as a major finding. However, experimental validation is limited to transcriptomic data. Functional tests, such as the use of Wnt agonist/inhibitor, are needed to support this claim (see below).

We agree that the suggested experiments could provide additional mechanistic insights into this study and will consider them in future work.

(g) Suggested fixes / experiments

Add at least one more independent disease hiPSC line (or show expanded analysis from GD2-10-257) for key mechanistic endpoints (lipid accumulation, transcriptomics, DA markers).

Additional line iPSC GD2-10-257 derived MLO was included in the manuscript. This was addressed above [see response to Weaknesses (1)-a].

Generate and analyze a fully corrected isogenic WT/WT clone (or a P415R-only line) if feasible; at minimum, acknowledge this limitation more explicitly and soften claims.

We attempted to generate an isogenic iPSC line by correcting both GBA1 mutations (L444P and P415R). However, this was unsuccessful because the GBA1 gene overlaps with a pseudogene (PGBA) located16kd downstream of GBA1, which shares 9698% sequence similarity with GBA1 (Ref#1, #2), which complicates precise editing. GBA1 is shorter (~5.7 kb) than PGBA (~7.6 kb). The primary exonic difference between GBA1 and PGBA is a 55-bp deletion in exon 9 of the pseudogene. As a result, the isogenic line we obtained carries only the P415R mutation, and L444P was corrected to normal sequence. We have included this limitation in the Methods as “This gene editing strategy is expected to also target the GBA1 pseudogene due to the identical target sequence, which limits the gene correction on certain mutations (e.g., P415R)”.

References:

(1) Horowitz M., Wilder S., Horowitz Z., Reiner O., Gelbart T., Beutler E. The human glucocerebrosidase gene and pseudogene: structure and evolution. Genomics (1989). 4, 87–96. doi:10.1016/0888-7543(89)90319-4

(2) Woo EG, Tayebi N, Sidransky E. Next-Generation Sequencing Analysis of GBA1: The Challenge of Detecting Complex Recombinant Alleles. Front Genet. (2021). 12:684067. doi: 10.3389/fgene.2021.684067. PMCID: PMC8255797.

Report and increase independent differentiations (N = biological replicates) and present per-differentiation summary statistics.

This was addressed above [see response to Weaknesses (1)-b, (1)-c].

(2) Mechanistic validation is insufficient

(a) RNA-seq pathways (Wnt, mTOR, lysosome) are not functionally probed. The manuscript shows pathway enrichment and some protein markers (p-4E-BP1) but lacks perturbation/rescue experiments to link these pathways causally to the DA phenotype.

(b) Autophagy analysis lacks flux assays. LC3-II and LAMP1 are informative, but without flux assays (e.g., bafilomycin A1 or chloroquine), one cannot distinguish increased autophagosome formation from decreased clearance.

(c) Dopaminergic dysfunction is superficially assessed. Dopamine in the medium and TH protein are shown, but no neuronal electrophysiology, synaptic marker co-localization, or viability measures are provided to demonstrate functional recovery after therapy.

(d) Suggested fixes / experiments - Perform targeted functional assays:

(i) Wnt reporter assays (TOP/FOP flash) and/or treat organoids with Wnt agonists/antagonists to test whether Wnt modulation rescues DA differentiation.

(ii) Test mTOR pathway causality using mTOR inhibitors (e.g., rapamycin) or 4E-BP1 perturbation and assay effects on DA markers and autophagy.

Include autophagy flux assessment (LC3 turnover with bafilomycin), and measure cathepsin activity where relevant.

Add at least one functional neuronal readout: calcium imaging, MEA recordings, or synaptic marker quantification (e.g., SYN1, PSD95) together with TH colocalization.

We thank the reviewer for these valuable suggestions. We agree that the suggested experiments could provide additional mechanistic insights into this study and will consider them in future work. Importantly, the primary conclusions of our manuscript, that GBA1 mutations in nGD MLOs resulted in nGD pathologies such as diminished enzymatic function, accumulation of lipid substrates, widespread transcriptomic changes, and impaired dopaminergic neuron differentiation, which can be corrected by several therapeutic strategies in this study, are supported by the evidence presented. The suggested experiments represent an important direction for future research using brain organoids.

(3) Therapeutic evaluation needs greater depth and standardization

(a) Short windows and limited durability data. SapC-DOPS and AAV9 experiments range from 48 hours to 3 weeks; longer follow-up is needed to assess durability and whether biochemical rescue translates into restored neuronal function.

We agree with the reviewer. Because this is a proof-of-principle study, the treatment was designed within a short time window. Long-term studies with more comprehensive outcome assessments will be conducted in future work.

(b) Dose-response and biodistribution are under-characterized. AAV injection sites/volumes are described, but transduction efficiency, vg copies per organoid, cell-type tropism quantification, and SapC-DOPS penetration/distribution are not rigorously quantified.

We appreciate the reviewer’s concerns. This study was intended to demonstrate the feasibility and initial response of MLOs to AAV therapy. A comprehensive evaluation of AAV biodistribution will be considered in future studies.

The penetration and distribution of SapC-DOPS have been extensively characterized in prior studies. In vivo biodistribution of SapC–DOPS coupled CellVue Maroon, a fluorescent cargo, was examined in mice bearing human tumor xenografts using real-time fluorescence imaging, where CellVue Maroon fluorescence in tumor remained for 48 hours (Ref. #3: Fig. 4B, mouse 1), 100 hours (Ref. #4: Fig. 5), up to 216 hours (Ref. #5: Fig. 3). Uptake kinetics were also demonstrated in cells, with flow cytometry quantification showing that fluorescent cargo coupled SapC-DOPS nanovesicles, were incorporated into human brain tumor cell membranes within minutes and remained stably incorporated into the cells for up to one hour (Ref. # 6: Fig. 1a and Fig. 1b). Building on these findings, the present study focuses on evaluating the restoration of GCase function rather than reexamining biodistribution and uptake kinetics.

References:

(3) X. Qi, Z. Chu, Y.Y. Mahller, K.F. Stringer, D.P. Witte, T.P. Cripe. Cancer-selective targeting and cytotoxicity by liposomal-coupled lysosomal saposin C protein. Clin. Cancer Res. (2009) 15, 5840-5851. PMID: 19737950.

(4) Z. Chu, S. Abu-Baker, M.B. Palascak, S.A. Ahmad, R.S. Franco, and X. Qi. Targeting and cytotoxicity of SapC-DOPS nanovesicles in pancreatic cancer. PLOS ONE (2013) 8, e75507. PMID: 24124494.

(5) Z. Chu, K. LaSance, V.M. Blanco, C-H. Kwon, B. Kaur, M. Frederick, S. Thornton, L. Lemen, and X. Qi. Multi-angle rotational optical imaging of brain tumors and arthritis using fluorescent SapC-DOPS nanovesicles. J. Vis. Exp. (2014) 87, e51187, 1-7. PMID: 24837630.

(6) J. Wojton, Z. Chu, C-H. Kwon, L.M.L. Chow, M. Palascak, R. Franco, T. Bourdeau, S. Thornton, B. Kaur, and X. Qi. Systemic delivery of SapC-DOPS has antiangiogenic and antitumor effects against glioblastoma. Mol. Ther. (2013) 21, 1517-1525. PMID: 23732993.

(c) Specificity controls are missing. For SapC-DOPS, inclusion of a non-functional enzyme control (or heat-inactivated fGCase) would rule out non-specific nanoparticle effects. For AAV, assessment of off-target expression and potential cytotoxicity is needed.

Including inactive fGCase would confound the assessment of fGCase in MLOs by immunoblot and immunofluorescence; therefore, saposin C–DOPS was used as the control instead.

We agree that assessment of off-target expression and potential cytotoxicity for AAV is important, this will be included in future studies.

(d) Comparative efficacy lacking. It remains unclear which modality is most effective in the long term and in which cellular compartments.

To address this comment, we have added a new table (Supplementary Table 2) comparing the four therapeutic modalities and summarizing their respective outcomes. While this study focused on short-term responses as a proof-of-principle, future work will explore long-term therapeutic effects.

(e) Suggested fixes/experiments

Extend follow-up (e.g., 6+ weeks) after AAV/SapC dosing and evaluate DA markers, electrophysiology, and lipid levels over time.

We appreciate the reviewer’s suggestions. The therapeutic testing in patient-derived MLOs was designed as a proof-of-principle study to demonstrate feasibility and the primary response (rescue of GCase function) to the treatment. A comprehensive, long-term therapeutic evaluation of AAV and SapC-DOPS-fGCase is indeed important for a complete assessment; however, this represents a separate therapeutic study and is beyond the scope of the current work.

Quantify AAV transduction by qPCR for vector genomes and by cell-type quantification of GFP+ cells (neurons vs astrocytes vs progenitors).

For the AAV-treated experiments, we agree that measuring AAV copy number and GFP expression would provide additional information. However, the primary goal of this study was to demonstrate the key therapeutic outcome, rescue of GCase function by AAV-delivered normal GCase, which is directly relevant to the treatment objective.

Include SapC-DOPS control nanoparticles loaded with an inert protein and/or fluorescent cargo quantitation to show distribution and uptake kinetics.

As noted above [see response to Weakness (3)-c], using inert GCase would confound the assessment of fGCase uptake in MLOs; therefore, it was not suitable for this study. See response above for the distribution and uptake kinetics of SapC-DOPS [see response to Weaknesses (3)-b].

Provide head-to-head comparative graphs (activity, lipid clearance, DA restoration, and durability) with statistical tests.

We have added a new table (Supplementary Table 2) providing a head-to-head comparison of the treatment effects.

(4) Model limitations not fully accounted for in interpretation

(a) Absence of microglia and vasculature limits recapitulation of neuroinflammatory responses and drug penetration, both of which are important in nGD. These absences could explain incomplete phenotypic rescues and must be emphasized when drawing conclusions about therapeutic translation.

We agree that the absence of microglia and vasculature in midbrain-like organoids represents a limitation, as we have discussed in the manuscript. In this revision, we highlighted this limitation in the Discussion section and clarified that it may contribute to incomplete phenotyping and phenotypic rescue observed in our therapeutic experiments. Additionally, we have outlined future directions to incorporate microglia and vascularization into the organoid system to better recapitulate the in vivo environment and improve translational relevance (see 7th paragraph in the Discussion).

(b) Developmental vs degenerative phenotype conflation. Many phenotypes appear during differentiation (patterning defects). The manuscript sometimes interprets these as degenerative mechanisms; the distinction must be clarified.

We appreciate the reviewer’s comments. In the revised manuscript, we have clarified that certain abnormalities, such as patterning defects observed during early differentiation, likely reflect developmental consequences of GBA1 mutations rather than degenerative processes. Conversely, phenotypes such as substrate accumulation, lysosomal dysfunction, and impaired dopaminergic maturation at later stages are interpreted as degenerative features. We have updated the Results and Discussion sections to avoid conflating developmental defects with neurodegenerative mechanisms.

(c) Suggested fixes

Tone down the language throughout (Abstract/Results/Discussion) to avoid overstatement that MLOs fully recapitulate nGD neuropathology.

The manuscript has been revised to avoid overstatements.

Add plans or pilot data (if available) for microglia incorporation or vascularization to indicate how future work will address these gaps.

The manuscript now includes further plans to address the incorporation of microglia and vascularization, described in the last two paragraphs in the Discussion. Pilot study of microglia incorporation will be reported when it is completed.

(5) Statistical and presentation issues

(a) Missing or unclear sample sizes (n). For organoid-level assays, report the number of organoids and the number of independent differentiations.

We have clarified biological replicates and differentiation in the figure legend [see response to Weaknesses (1)-b, (1)-c].

(b) Statistical assumptions not justified. Tests assume normality; where sample sizes are small, consider non-parametric tests and report exact p-values.

We have updated Statistical analysis in methods as described below:

For comparisons between two groups, data were analyzed using unpaired two-tailed Student’s t-tests when the sample size was ≥6 per group and normality was confirmed by the Shapiro-Wilk test. When the normality assumption was not met or when sample sizes were small (n < 6), the non-parametric Mann-Whitney U test was used instead. For comparisons involving three or more groups, one-way ANOVA followed by Tukey’s multiple comparison test was applied when data were normally distributed; otherwise, the nonparametric Dunn’s multiple comparison test was used. Exclusion of outliers was made based on cutoffs of the mean ±2 standard deviations. All statistical analyses were performed using GraphPad Prism 10 software. Exact p-values are reported throughout the manuscript and figures where feasible. A p-value < 0.05 was considered statistically significant.

(c) Quantification scope. Many image quantifications appear to be from selected fields of view, which are then averaged across organoids and differentiations.

In this work, quantitative immunofluorescence analyses (e.g., cell counts for FOXP1+, FOXG1+, SOX2+ and Ki67+ cells, as well as marker colocalization) were performed on at least 3–5 randomly selected non-overlapping fields of view (FOVs) per organoid section, with a minimum of 3 organoids per differentiation batch. Each FOV was imaged at consistent magnification (60x) and z-stack depth to ensure comparable sampling across conditions. Data from individual FOVs were first averaged within each organoid to obtain an organoid-level mean, and then biological replicates (independent differentiations, n ≥ 3) were averaged to generate the final group mean ± SEM. This multilevel averaging approach minimizes bias from regional heterogeneity within organoids and accounts for variability across differentiations. Representative confocal images shown in the figures were selected to accurately reflect the quantified data. We believe this standardized quantification strategy ensures robust and reproducible results while appropriately representing the 3D architecture of the organoids.

In the revision, we have clarified the method used for image analysis of sectioned MLOs as below:

Quantitative immunofluorescence analyses (e.g., cell counts for FOXP1+, FOXG1+, SOX2+ and Ki67+ cells, as well as marker colocalization) were performed using ImageJ (NIH) on at least 3–5 randomly selected non-overlapping fields of view (FOVs) per organoid section, with a minimum of 3 organoids per differentiation batch. Each FOV was imaged at consistent magnification (60x) and z-stack depth to ensure comparable sampling across conditions. Data from individual FOVs were first averaged within each organoid to obtain an organoid-level mean, and then biological replicates (independent differentiations, n ≥ 3) were averaged to generate the final group mean ± SEM.

(d) RNA-seq QC and deposition. Provide mapping rates, batch correction details, and ensure the GEO accession is active. Include these in Methods/Supplement.

RNA-seq data are from same batch. The mapping rate is >90%. GEO accession will be active upon publication. These were included in the Methods.

(e) Suggested fixes

Add a table summarizing biological replicates, technical replicates, and statistical tests used for each figure panel.

We have revised the figure legends to include replicates for each figure and statistical tests [see response in weaknesses (1)-b, (1)-c].

Recompute statistics where appropriate (non-parametric if N is small) and report effect sizes and confidence intervals.

Statistical analysis method is provided in the revision [see response in Weaknesses (5)-b].

(6) Minor comments and clarifications

(a) The authors should validate midbrain identity further with additional regional markers (EN1, OTX2) and show absence/low expression of forebrain markers (FOXG1) across replicates.

We validated the MLO identity by (1) FOXG1 and (2) EN1. FOXG1 was barely detectable in Wk8 75.1_MLO but highly present in ‘age-matched’ cerebral organoid (CO), suggesting our culturing method is midbrain region-oriented. In nGD MLO, FOXG1 expression is significantly higher than 75.1_MLO, indicating that there was aberrant anterior-posterior brain specification, consistent with the transcriptomic dysregulation observed in our RNA-seq data.

To further confirm midbrain identity, we examined the expression of EN1, an established midbrain-specific marker. Quantitative RT-PCR analysis demonstrated that EN1 expression increased progressively during differentiation in both WT-75.1 and nGD2-1260 MLOs at weeks 3 and 8 (Author response image 1). EN1 reached 34-fold and 373-fold higher levels than in WT-75.1 iPSCs at weeks 3 and 8, respectively, in WT-75.1 MLOs. In nGD MLOs, although EN1 expression showed a modest reduction at week 8, the levels were not significantly different from those observed in age-matched WT-75.1 MLOs (p > 0.05, ns).

**Author response image 1.** qRT-PCR quantification of midbrain progenitor marker *EN1* expression in WT-75. 1 and GD2-1260 MLOs at Wk3 and Wk8. Data was normalized to WT-75.1 hiPSC cells and presented as mean ± SEM (n = 3-4 MLOs per group). ns, not significant.

(b) Extracellular dopamine ELISA should be complemented with intracellular dopamine or TH+ neuron counts normalized per organoid or per total neurons.

We quantified TH expression at both the mRNA level (Fig. 3F) and the protein level (Fig. 3G/H) from whole-organoid lysates, which provides a more consistent and integrative measure across samples. These TH expression levels correlated well with the corresponding extracellular (medium) dopamine concentrations for each genotype. In contrast, TH^+^ neuron counts may not reliably reflect total cellular dopamine levels because the number of cells captured on each organoid section varies substantially, making normalization difficult. Measuring intracellular dopamine is an alternative approach that will be considered in future studies.

(c) For CRISPR editing: the authors should report off-target analysis (GUIDE-seq or targeted sequencing of predicted off-targets) or at least in-silico off-target score and sequencing coverage of the edited locus. (off-target analysis (GUIDE-seq or targeted sequencing of predicted off-targets) or at least in-silico off-target score and sequencing coverage of the edited locus).

The off-target effect was analyzed during gene editing and the chance to target other off-targets is low due to low off-target scores ranked based on the MIT Specificity Score analysis. The related method was also updated as stated below:

“The chance to target other off-targets is low due to low off-target scores ranked based on the MIT Specificity Score analysis (Hsu, P., Scott, D., Weinstein, J. et al. DNA targeting specificity of RNA-guided Cas9 nucleases. Nat Biotechnol 31, 827–832 (2013). https://doi.org/10.1038/nbt.2647).”

(d) It should be clarified as to whether lipidomics normalization is to total protein per organoid or per cell, and include representative LC-MS chromatograms or method QC.

The normalization was to the protein of organoid lysate. This was clarified in the Methods section in the revision as stated below:

“The GluCer and GluSph levels in MLO were normalized to total MLO protein (mg) that were used for glycosphingolipids analyses. Protein mass was determined by BCA assay and glycosphingolipid was expressed as pmol/mg protein. Additionally, GluSph levels in the culture medium were quantified and normalized to the medium volume (pmol/mL).”

Representative LC-MS chromatograms for both normal and GD MLOs have been included in a new figure, Supplementary Figure 2.

(e) Figure legends should be improved in order to state the number of organoids, the number of differentiations, and the exact statistical tests used (including multiplecomparison corrections).

This was addressed above [see response to Weaknesses (1)-b and (5)-b].

(f) In the title, the authors state "reveal disease mechanisms", but the studies mainly exhibit functional changes. They should consider toning down the statement.

The title was revised to: Patient-Specific Midbrain Organoids with CRISPR Correction Recapitulate Neuronopathic Gaucher Disease Phenotypes and Enable Evaluation of Novel Therapies

(7) Recommendations

This reviewer recommends a major revision. The manuscript presents substantial novelty and strong potential impact but requires additional experimental validation and clearer, more conservative interpretation. Key items to address are:

(a) Strengthening genetic and biological replication (additional lines or replicate differentiations).

This was addressed above [see response to Weaknesses (1)-a, (1)-b, (1)-c].

(b) Adding functional mechanistic validation for major pathways (Wnt/mTOR/autophagy) and providing autophagy flux data.

(c) Including at least one neuronal functional readout (calcium imaging/MEA/patch) to demonstrate functional rescue.

As addressed above [see response to Weaknesses (2)], the suggested experiments in (b) and (c) would provide additional insights into this study and we will consider them in future work.

(d) Deepening therapeutic characterization (dose, biodistribution, durability) and including specificity controls.

This was addressed above [see response to Weaknesses (3)-a to e].

(e) Improving statistical reporting and explicitly stating biological replicate structure.

This was addressed above [see response to Weaknesses (1)-b, (5)-b].

**Reviewer #2 (Public review):**

Sun et al. have developed a midbrain-like organoid (MLO) model for neuronopathic Gaucher disease (nGD). The MLOs recapitulate several features of nGD molecular pathology, including reduced GCase activity, sphingolipid accumulation, and impaired dopaminergic neuron development. They also characterize the transcriptome in the MLO nGD model. CRISPR correction of one of the GBA1 mutant alleles rescues most of the nGD molecular phenotypes. The MLO model was further deployed in proof-of-principle studies of investigational nGD therapies, including SapC-DOPS nanovesicles, AAV9-mediated GBA1 gene delivery, and substrate-reduction therapy (GZ452). This patient-specific 3D model provides a new platform for studying nGD mechanisms and accelerating therapy development. Overall, only modest weaknesses are noted.

We thank the reviewer for the supportive remarks.

**Reviewer #3 (Public review):**

Summary:

In this study, the authors describe modeling of neuronopathic Gaucher disease (nGD) using midbrain-like organoids (MLOs) derived from hiPSCs carrying GBA1 L444P/P415R or L444P/RecNciI variants. These MLOs recapitulate several disease features, including GCase deficiency, reduced enzymatic activity, lipid substrate accumulation, and impaired dopaminergic neuron differentiation. Correction of the GBA1 L444P variant restored GCase activity, normalized lipid metabolism, and rescued dopaminergic neuronal defects, confirming its pathogenic role in the MLO model. The authors further leveraged this system to evaluate therapeutic strategies, including: (i) SapC-DOPS nanovesicles for GCase delivery, (ii) AAV9-mediated GBA1 gene therapy, and (iii) GZ452, a glucosylceramide synthase inhibitor. These treatments reduced lipid accumulation and ameliorated autophagic, lysosomal, and neurodevelopmental abnormalities.

Strengths:

This manuscript demonstrates that nGD patient-derived MLOs can serve as an additional platform for investigating nGD mechanisms and advancing therapeutic development.

Comments:

(1) It is interesting that GBA1 L444P/P415R MLOs show defects in midbrain patterning and dopaminergic neuron differentiation (Figure 3). One might wonder whether these abnormalities are specific to the combination of L444P and P415R variants or represent a general consequence of GBA1 loss. Do GBA1 L444P/RecNciI (GD2-10-257) MLOs also exhibit similar defects?

We observed reduced dopaminergic neuron marker TH expression in GBA1 L444P/RecNciI (GD2-10-257) MLOs, suggesting that this line also exhibits defects in dopaminergic neuron differentiation. These data are provided in a new Supplementary Fig. 4E, and are summarized in new Supplementary Table 2 in the revision.

(2) In Supplementary Figure 3, the authors examined GCase localization in SapC-DOPSfGCase-treated nGD MLOs. These data indicate that GCase is delivered to TH^+^ neurons, GFAP^+^ glia, and various other unidentified cell types. In fruit flies, the GBA1 ortholog, Gba1b, is only expressed in glia (PMID: 35857503; 35961319). Neuronally produced GluCer is transferred to glia for GBA1-mediated degradation. These findings raise an important question: in wild-type MLOs, which cell type(s) normally express GBA1? Are they dopaminergic neurons, astrocytes, or other cell types?

All cell types in wild-type MLOs are expected to express GBA1, as it is a housekeeping gene broadly expressed across neurons, astrocytes, and other brain cell types. Its lysosomal function is essential for cellular homeostasis and is therefore not restricted to any specific lineage. (https://www.proteinatlas.org/ENSG00000177628GBA1/brain/midbrain).

(3) The authors may consider switching Figures 2 and 3 so that the differentiation defects observed in nGD MLOs (Figure 3) are presented before the analysis of other phenotypic abnormalities, including the various transcriptional changes (Figure 2).

We appreciate the reviewer’s suggestion; however, we respectfully prefer to retain the current order of Figures 2 and 3, as we believe this structure provides the clearest narrative flow. Figure 2 establishes the core biochemical hallmarks: reduced GCase activity, substrate accumulation, and global transcriptomic dysregulation (1,429 DEGs enriched in neural development, WNT signaling, and lysosomal pathways), which together provide essential molecular context for studying the specific cellular differentiation defects presented in Figure 3. Presenting the broader disease landscape first creates a coherent mechanistic link to the subsequent analyses of midbrain patterning and dopaminergic neuron impairment.

To enhance readability, we have added a brief transitional sentence at the start of the Figure 3 paragraph: “Building on the molecular and transcriptomic hallmarks of GCase deficiency observed in nGD MLOs (Figure 2), we next investigated the impact on midbrain patterning and dopaminergic neuron differentiation (Figure 3).”

**Recommendations for the authors:**

**Reviewing Editor Comments:**

Your paper has been reviewed by three expert reviewers in the GBA field. Although they appreciate the work and its novelty, they raise several concerns. We suggest that you to address these concerns in the next version.

**Reviewer #1 (Recommendations for the authors):**

Statistical and presentation issues

(1) Missing or unclear sample sizes (n). For organoid-level assays, report the number of organoids and the number of independent differentiations.

This was addressed above [see response to Reviewer 1 Weaknesses (1)- b].

(2) Statistical assumptions not justified. Tests assume normality; where sample sizes are small, consider non-parametric tests and report exact p-values.

We have updated methods to describe the Statistical analysis details [see response to Reviewer 1 Weaknesses (5)-b].

(3) Quantification scope. Many image quantifications appear to be from selected fields of view, which are then averaged across organoids and differentiations.

This was addressed above [see response to Reviewer 1 Weaknesses (5)- c].

(4) RNA-seq QC and deposition. Provide mapping rates, batch correction details, and ensure the GEO accession is active. Include these in Methods/Supplement.

Our RNA-seq data were generated from a single batch of MLOs, with mapping rates exceeding 90%. The GEO accession will be made publicly available upon publication.

**Reviewer #2 (Recommendations for the authors):**

Please consider the following suggestions for revisions:

(1) Line 86: A bit more explanation/justification for the focus on midbrain-like organoids would be helpful, including introducing the nature of the midbrain pathology to better put some of the MLO findings in context. Is the nGD pathology for the midbrain significantly different / out of proportion to other affected brain regions?

nGD Patients often display impaired vertical gaze and movement disorders. These symptoms correlate with midbrain involvement due to the sensitivity of this region to neuroinflammatory and degenerative processes (Ref #7, #8). Both human and mouse studies indicate that the midbrain exhibits prominent substrate accumulation compared to other brain regions, suggesting a predisposition for greater pathological involvement in GD midbrain (Ref #8, #9, #10, #11). This rationale was added to Line 86 in the revision.

References:

(7) Goker-Alpan O, Ivanova MM. Neuronopathic Gaucher disease: Rare in the West, common in the East. J Inherit Metab Dis.(2024) 47(5):917-934. PMID: 38768609.

(8) Burrow TA, Sun Y, Prada CE, Bailey L, Zhang W, Brewer A, Wu SW, Setchell KDR, Witte D, Cohen MB, Grabowski GA. CNS, lung, and lymph node involvement in Gaucher disease type 3 after 11 years of therapy: clinical, histopathologic, and biochemical findings. Mol Genet Metab. (2015) 114(2):233-241. PMID: 25219293.

(9) Tamar Farfel-Becker, Einat B. Vitner, Samuel L. Kelly, Jessica R. Bame, Jingjing Duan, Vera Shinder, Alfred H. Merrill, Kostantin Dobrenis, Anthony H. Futerman. Neuronal accumulation of glucosylceramide in a mouse model of neuronopathic Gaucher disease leads to neurodegeneration, Human Molecular Genetics, (2014). Volume 23, Issue 4, Pages 843–854.

(10) E. Ellen Jones, Wujuan Zhang, Xueheng Zhao, Cristine Quiason , Stephanie Dale, Sheerin Shahidi-Latham, Gregory A. Grabowski, Kenneth D. R. Setchell, Richard R. Drake, and Ying Sun. High-Resolution MALDI Imaging Mass Spectrometry. SLAS Discovery (2017). Vol. 22(10) 1218–1228

(11) Xu YH, Xu K, Sun Y, Liou B, Quinn B, Li RH, Xue L, Zhang W, Setchell KD, Witte D, Grabowski GA. Multiple pathogenic proteins implicated in neuronopathic Gaucher disease mice. Hum Mol Genet. (2014) 23(15):3943-57. PMID: 24599400.

(2) Lines 359-360: Please specify the carbon-chain length of the sphingoid base of the GluCer species analyzed. Also, is there a citation for the statement that 18:0 and 16:0 are "brain-enriched species"?

The carbon-chain length analyzed ranges from 14:0 to 24:0. The sphingoid base for all GluCer species analyzed is d18:1. For example, the species referred to as GluCer 18:0 corresponds to GluCer(d18:1/18:0). Although both, 16:0 and 18:0 are enriched in the brain, 18:0 is the most abundant species in the brain (Ref #12, #13). We revised "brain-enriched species” to “brain-predominant species (18:0)”.

References:

(12) Nilsson, O., and Svennerholm, L. Accumulation of Glucosylceramide and Glucosylsphingosine (Psychosine) in Cerebrum and Cerebellum in Infantile and Juvenile Gaucher Disease. Journal of Neurochemistry (1982) 39, 709–718.

(13) Sun, Y., Zhang, W., Xu, Y.H., Quinn, B., Dasgupta, N., Liou, B., Setchell, K.D., and Grabowski, G.A. Substrate compositional variation with tissue/region and Gba1 mutations in mouse models--implications for Gaucher disease. PLoS One (2013). 8, e57560.10.1371/journal.pone.0057560.

(3) Figure 2: It would be interesting to compare the MLO findings to prior gene expression data. Are there previously published transcriptome analyses from nGD brain tissue (or other tissues) that the transcriptome data obtained from MLOs may be compared with? What about transcriptome analyses of mouse GD models?

We thank the reviewer for this valuable suggestion. To strengthen the biological context of our transcriptomic findings, we have added a new comparative table (new Supplementary Table 3) in the revised manuscript that summarizes key dysregulated pathways in our human nGD MLOs alongside previously published data from nGD mouse midbrain (Ref#14). The table highlights substantial overlap, including axon guidance, neuron differentiation, dopaminergic/glutamatergic/GABAergic synaptic signaling, lipid metabolism, apoptosis/cell death, and nervous system development, emphasizing the translational relevance of our model. We also note that our dataset uniquely reveals pronounced dysregulation of WNT signaling and anterior-posterior patterning (Fig. 2L and 2M), potentially reflecting human-specific early midbrain defects.

We added the following sentence to Discussion: “Comparative analysis with prior transcriptomic data from nGD mouse midbrain showed consistent dysregulation in axon guidance, synaptic signaling, lipid metabolism, and nervous system development (new Supplementary Table 3), supporting the fidelity of our human MLO model.”

Reference:

(14) Dasgupta N, Xu YH, Li R, Peng Y, Pandey MK, Tinch SL, Liou B, Inskeep V, Zhang W, Setchell KD, Keddache M, Grabowski GA, Sun Y. Neuronopathic Gaucher disease: dysregulated mRNAs and miRNAs in brain pathogenesis and effects of pharmacologic chaperone treatment in a mouse model. Hum Mol Genet. (2015) 24(24):7031-48. PMID: 26420838.

(4) Lines 402-405 & Figure 3D: Is it possible to include a merged image to better visualize the TH and FOXA2 co-staining / potential colocalization?

The merged images of TH (red) and FOXA2 (green) are shown in Fig. 3E. Yellow arrows indicate TH and FOXA2 co-stained cells, which appear yellow in the merged images. The results demonstrate that the number of co-stained cells is reduced in GD2-1260 MLOs compared with WT-75.1 MLOs at both, week 6 and week 8.

(5) Lines 447-448 & Figure 4F, G, J: It would be helpful to provide a direct analysis/visualization of MLO size between the WT-75.1, GD2-1260, and iso-GD2-1260 genotypes (allowing direct comparison of WT and iso). Similarly, the same 3-way analysis would be valuable for assessing dopamine levels.

We have included WT-75.1 in Fig. 4 F/G/J in the revision. All three genotypes, WT-75.1, GD2-1260, and iso-GD2-1260, are presented for analysis compared to WT-75.1. In new Figure 4F, MLO growth is presented by representative MLO images taken under wide field microscopy at day 2, Wk4 and Wk8 of differentiation. In new Fig. 4G, MLOs size was analyzed by NIS elements and presented as the area (µm^2^) of MLO in image (mean ± SEM). N≥10 MLOs were analyzed for each genotype. In new Fig. 4J. Dopamine levels in MLO culture medium from WT-75.1, GD2-1260 and iso- GD2-1260 MLOs at Wk12 cultured in 3 mL BGM medium for 72 hours were analyzed. Data are presented as mean ± SEM (n = 5 per group). Statistical analysis applied was described in the legend.

(6) Figure 4: What is the explanation/interpretation of the residual autophagy pathway dysfunction in CRISPR-corrected MLOs? nGD requires near-complete loss of GCase activity, so it is a bit curious that autophagic dysfunction would be observed with only ~50% GCase reduction? There is some discussion, but it doesn't fully capture the unexpected nature and implications of this result.

This phenomenon may be explained by a threshold effect in lysosomal function. Gaucher disease is an autosomal recessive disorder. The carriers with heterozygous GBA1 mutation, who retain approximately 50% of normal GCase activity, do not develop disease. This suggests that even partial restoration of GCase activity can reduce glucosylceramide accumulation below a pathological threshold, thereby restoring lysosomal integrity and autophagic flux. In addition, improved GCase activity may help normalize the lipid composition of lysosomal membranes, facilitating the fusion events required for effective autophagy.

(7) Lines 512-516 & Figure 5J: The data shown are inconclusive. Can these Western blot data be quantified, noting the number of replicates for each measurement? Without quantification and statistics, it is difficult to assess the claim that levels of LAMP1, LC3-I, LC3-II, 4E-BP1, and p-4E-BP1 in GD2-1260 treated with SapC-DOPS-fGCase are more similar to GD2-1260 treated following SapC-DOPS than to WT-75.1.

We performed quantitative analysis by comparing WT-75.1 and included the data in new Fig. 5J. The result was revised as:

Analysis of protein levels showed that decreased LAMP1 expression in GD2 1260 MLOs was not altered following SapC DOPS fGCase treatment (Figure 5J). The elevated LC3-II levels, an indicator of impaired autophagic flux, were reduced upon treatment, suggesting enhanced autophagic activity (Figure 5J). Moreover, phosphorylated 4E-BP1 (Thr37/46), but not total 4E-BP1, was improved in SapC-DOPS-fGCase–treated MLOs, reflecting a decrease in mTOR hyperactivation (Figure 5J). We anticipate that a longer duration of SapC-DOPS-fGCase exposure in nGD MLOs may produce a more robust therapeutic effect in rescuing nGD-associated phenotypes, which will be evaluated in future studies.

(8) Lines 518-520: The presented data support "effective restoration of GCase activity," but clarification is needed regarding "correction of GD-related disease phenotypes." Perhaps "selected molecular and biochemical phenotypes" would be more accurate. Data are not shown for several other phenotypes, including TH, FOXA2, and dopamine levels.

This was revised to “selected molecular and biochemical phenotypes “.

(9) Figure 5D-J: Please clarify whether all experiments were conducted 48 hours after treatment, as indicated for Figure 5C. If so, does this suggest that SapC-DOPS treatment exhibits only short-term effects? Were any data collected to evaluate the persistence of the treatment effect?

The treatment duration is specified in the Fig. 5 legend. Fig. 5D–J represent experiments conducted after two weeks of treatment, whereas Fig. 5C reflects a 48-hour treatment. In both Gaucher disease lines, two-week treatment restored GCase activity to wild-type levels and reduced GluSph substrate accumulation. These findings were intended as proof-of-principle to demonstrate therapeutic feasibility; evaluation of treatment persistence beyond two weeks was beyond the scope of this study.

Minor suggestions

(1) Line 80: "A brain organoid derived from hiPSCs of a healthy individual with GBA1 knockout and α-synuclein overexpression exhibited some PD features23." I would suggest enumerating what "PD features" are to distinguish from "clinical features", which I don't think is the intended meaning.

This was revised as “exhibited characteristic PD markers”.

(2) Figure 2I: The reported number of downregulated DEGs is incorrect. It should be 765, not 1429.

This was corrected in Figure 2I.

(3) Line 359: change "enrich" to "enriched".

This word was corrected.
